# Homochiral oligomers with highly flexible backbones form stable H-bonded duplexes†
†Electronic supplementary information (ESI) available: Detailed experimental procedures and ^1^H, ^13^C and ^31^P NMR spectra of all compounds, NMR titration spectra, thermal denaturation spectra and molecular modelling methods. See DOI: 10.1039/c6sc02995g



**DOI:** 10.1039/c6sc02995g

**Published:** 2016-08-19

**Authors:** Diego Núñez-Villanueva, Christopher A. Hunter

**Affiliations:** a Department of Chemistry , University of Cambridge , Lensfield Road , Cambridge CB2 1EW , UK . Email: herchelsmith.orgchem@ch.cam.ac.uk

## Abstract

Two homochiral building blocks featuring a protected thiol, an alkene and a H-bond recognition unit (phenol or phosphine oxide) have been prepared. Iterative photochemical thiol–ene coupling reactions were used to synthesize oligomers containing 1-4 phosphine oxide and 1-4 phenol recognition sites. Length-complementary H-bond donor and H-bond acceptor oligomers were found to form stable duplexes in toluene. NMR titrations and thermal denaturation experiments show that the association constant and the enthalpy of duplex formation increase significantly for every additional H-bonding unit added to the chain. There is an order of magnitude increase in stability for each additional H-bonding interaction at room temperature indicating that all of the H-bonding sites are fully bound to their complements in the duplexes. The backbone of the thiol–ene duplexes is a highly flexible alkane chain, but this conformational flexibility does not have a negative impact on binding affinity. The average effective molarity for the intramolecular H-bonding interactions that zip up the duplexes is 18 mM. This value is somewhat higher than the EM of 14 mM found for a related family of duplexes, which have the same recognition units but a more rigid backbone prepared using reductive amination chemistry. The flexible thiol–ene AAAA·DDDD duplex is an order of magnitude more stable than the rigid reductive amination AAAA·DDDD duplex. The backbone of the thiol–ene system retains much of its conformational flexibility in the duplex, and these results show that highly flexible molecules can make very stable complexes, provided there is no significant restriction of degrees of freedom on complexation.

## Abstract 2

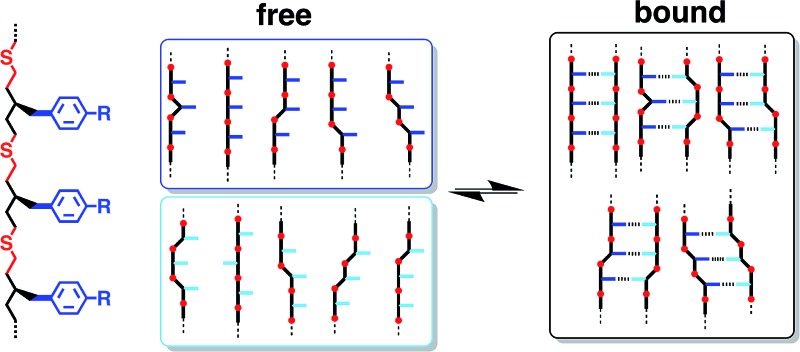
Highly flexible oligomers make stable duplexes, when conformational mobility is not significantly restricted in the bound state.

## Introduction

Biomolecules encode information about supramolecular structures and function as a sequence of different monomer units assembled into a linear polymer. Of particular importance is the role of nucleic acids in storing and reproducing genetic information, through the formation of sequence-selective duplexes and self-replication *via* template-directed synthesis.^1^ These specific properties have led to the design, study and application of synthetic structures based on nucleic acids as engineering materials for nanotechnology, such as molecular switches, aptamers and multi-component nanometer-scale assemblies.^2^ However, the properties that allow nucleic acids to encode genetic information are not currently available in any other material.

**Fig. 1:**
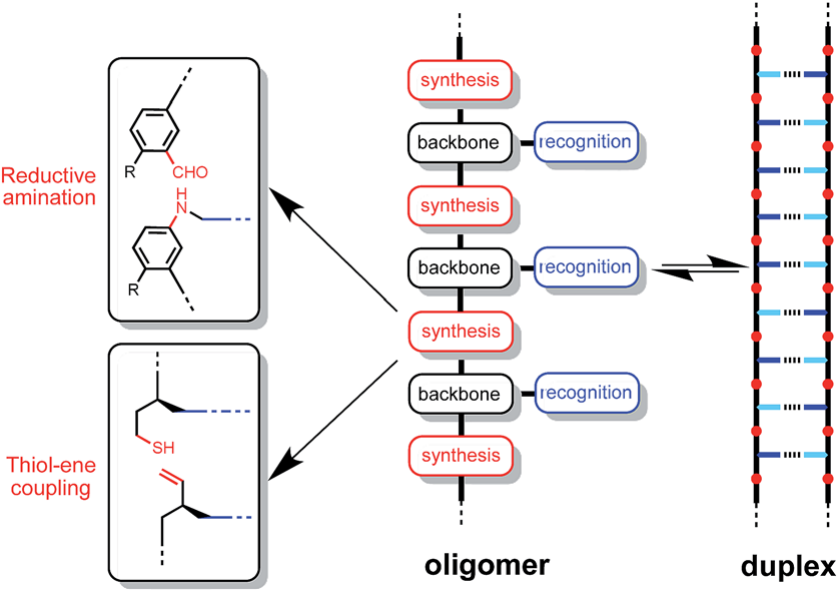
A blueprint for duplex forming molecules. There are three key design elements: the coupling chemistry used for the synthesis of oligomers (red), the recognition module which controls intermolecular binding (blue) and the backbone module which links these components together (black). Two different synthesis modules are highlighted: reductive amination and thiol–ene coupling. Adapted from ref. 10.

Significant effort has gone into making synthetic oligomers that can form bimolecular duplexes through different types of non-covalent interactions, such as metal–ligand coordination,^3^ aromatic stacking,^4^ salt bridges^5^ and H-bonding.^6^ The suitability of these oligomers as prototypes for synthetic information molecules is limited by the fact that the recognition sites are usually built into the backbone so that duplex formation requires precise matching of molecular geometries. The introduction of sequence variation into such systems is therefore difficult without disrupting the duplex. In contrast, the modular structure of nucleic acids allows modification of the base-pairing system independently of the backbone.^7–9^ We recently reported a new class of synthetic oligomers based on this blueprint (Fig. 1), which allows independent optimisation of the backbone (black), recognition sites (blue) and coupling chemistry used for synthesis of the oligomers (red).^10^ A variety of different oligomers have been prepared using the reductive amination chemistry illustrated in Fig. 1. These systems form stable H-bonded duplexes in toluene, and we have shown that it is possible to mix and match different backbone modules and different recognition modules without disrupting duplex formation.^11,12^

Here we explore the potential of using a different synthesis module, photochemical thiol–ene coupling (Fig. 1). Thiol–ene coupling appears to be an ideal reaction for oligomer synthesis: it is high yielding with excellent regioselectivity; the rates of reaction are fast under mild conditions; the radical reaction is compatible with a wide variety of functional groups that might be used as the recognition modules; the reaction can be carried out in the non-polar solvents required to promote H-bonding interactions between the recognition modules, and the resulting thioether linkage is non-polar and will not compete with H-bonding sites on the recognition modules.^13^ Moreover, thiol–ene polymerization has proven to be a useful reaction for materials synthesis,^14^ and nucleobase-containing homopolymers have been prepared using this reaction.^15^

**Fig. 2:**
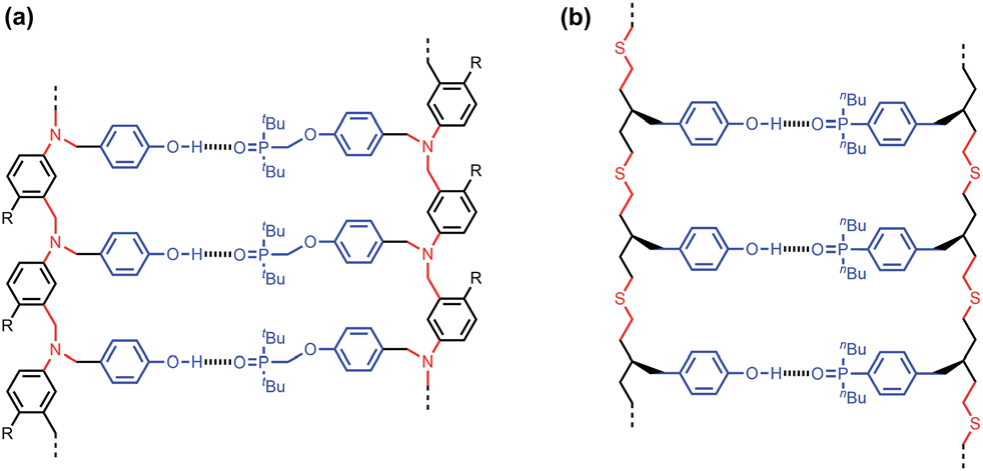
Duplex formed from a phenol oligomer and a phosphine oxide oligomer synthesized (a) using reductive amination chemistry and (b) using thiol–ene chemistry (R are 2-ethylhexoxy solubilising groups).^10^

Fig. 2a shows the structure of a duplex formed between a phenol oligomer and a phosphine oxide oligomer, which we have previously prepared using reductive amination chemistry. Fig. 2b shows the design of a similar duplex accessible *via* thiol–ene coupling. Phenol and phosphine oxide represent an ideal recognition module, because phenol is a very good H-bond donor and a very weak acceptor (H-bond acceptor parameter *β* ≈ 3), and phosphine oxide is one of the best H-bond acceptors known (*β* ≈ 10). The H-bond between these two functional groups is exceptionally strong in toluene (*K* ≈ 300 M^–1^).^16^ The thiol–ene synthesis module leads to thioether linkages (Fig. 2b), which are relatively non-polar (*β* ≈ 3), so there will be no competition with the phosphine oxide H-bond acceptors on the recognition modules.^17^ The backbone module shown in the design in Fig. 2b is chiral and therefore requires enantioselective synthesis of the monomer units. The influence of monomer chirality on duplex assembly is an interesting feature of this system that could be exploited at a future date. Here we describe the synthesis of homochiral phenol and phosphine oxide monomer units, the use of these building blocks to prepare oligomers, and NMR studies of duplex formation between length-complementary oligomers. One major difference between the reductive amination oligomers in Fig. 2a and the thiol–ene oligomers in Fig. 2b is the conformational flexibility of the backbone, and the consequences for duplex stability are quantified below.

## Results and discussion

### Synthesis

**Scheme 1:**
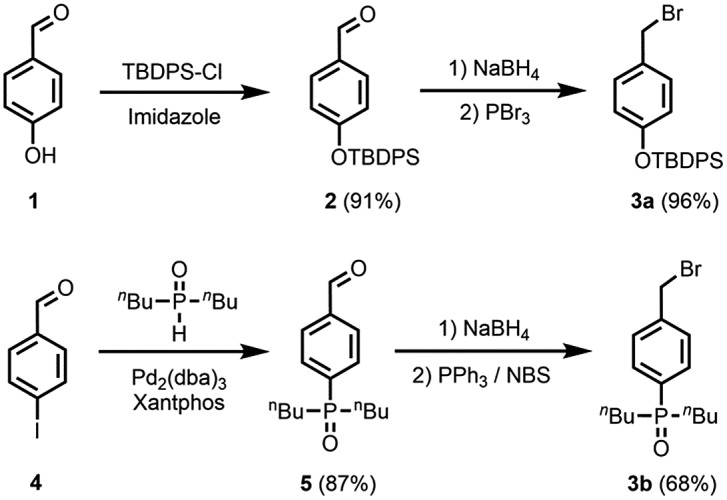


**Scheme 2:**
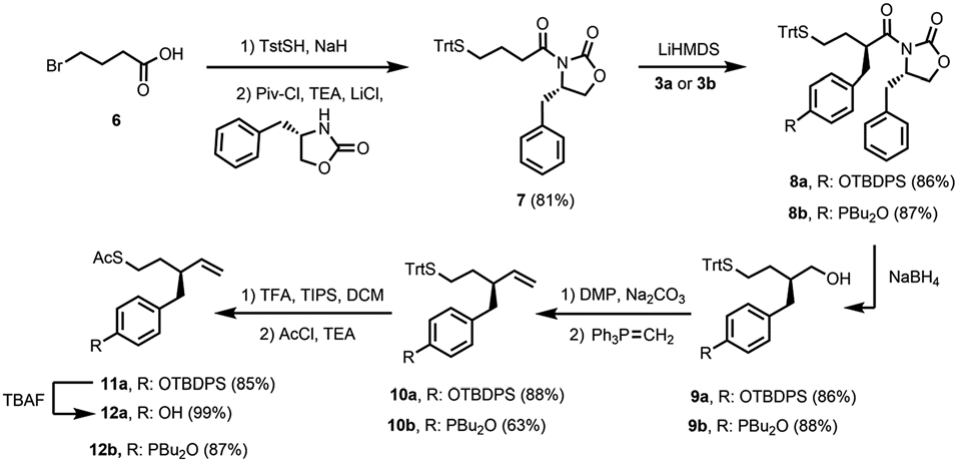


The synthesis of suitable monomers equipped with a thiol, an alkene and a recognition module is outlined in Schemes 1 and 2. The benzyl bromide derivatives of the recognition modules were prepared first (Scheme 1). The phenol group of 4-hydroxybenzaldehyde (**1**) was protected as a silyl ether, and the aldehyde was reduced with NaBH_4_. Bromination of the resulting alcohol with PBr_3_ yielded benzyl bromide **3a**. Palladium-mediated P-arylation of 4-iodobenzaldehyde (**4**) with di-*n*-butylphosphine oxide gave **5**. Reduction of **5** with NaBH_4_ and bromination of the resulting alcohol with *N*-bromosuccinimide (NBS) and triphenylphosphine provided benzyl bromide **3b**.

Scheme 2 shows the synthetic route to the monomer required for oligomer synthesis. The first step involved formation of imide **7** from commercially available 4-bromobutyric acid **6** using published procedures for incorporation of the *S*-trityl group and introduction of the 2-oxazolidinone chiral auxiliary.^18^ Lithium bis(trimethylsilyl)amide (LiHMDS) was used as the base to form the enolate of **7**, and alkylation with benzyl bromide **3a** or **3b** afforded **8a** and **8b**, respectively, as single diastereoisomers in good yields (dr > 95 : 5). Reduction of **8a** and **8b** removed the chiral auxiliary under mild conditions yielding alcohols **9a** and **9b**. Oxidation of these alcohols using Dess–Martin periodinane (DMP) followed by Wittig reactions of the aldehyde products with the ylide derived from methyltriphenylphosphonium bromide afforded **10a** and **10b**. The *S*-trityl group is not stable to radical conditions,^19^ so this protecting group was exchanged for an *S*-acetyl group: treatment of **10a** or **10b** with TFA in the presence of triisopropylsilane (TIPS) followed by acetylation with acetyl chloride yielded protected monomer **11a** and monomer **12b**, respectively. Removal of the silyl protecting group from **11a** afforded monomer **12a**. The synthetic route involves 6–7 steps and gives homochiral monomers **12a** and **12b** in overall yields of about 40%.

**Scheme 3:**
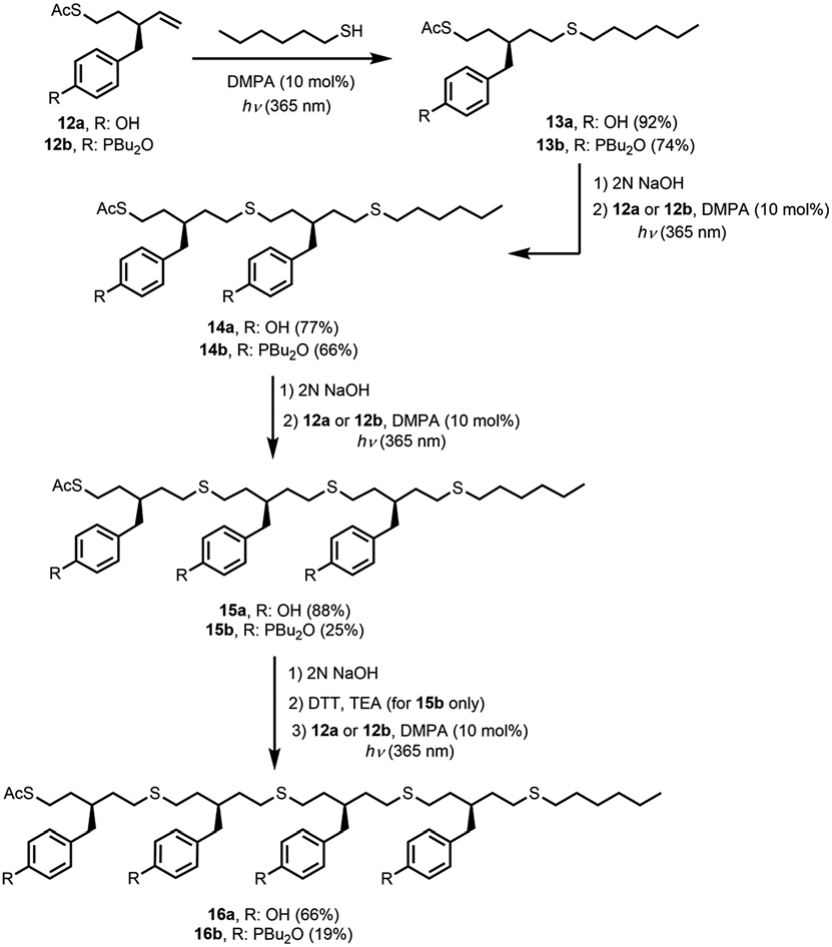


Oligomers were prepared in a stepwise manner from the monomer units using photochemically initiated thiol–ene coupling reactions (Scheme 3). Reaction of **12a** or **12b** with 1-hexanethiol under 365 nm irradiation in the presence of 2,2-dimethoxy-2-phenylacetophenone (DMPA) gave end-capped monomers **13a** and **13b**, respectively. Removal of the *S*-acetyl protecting group of **13a** and **13b** under basic conditions and subsequent thiol–ene coupling with monomer **12a** or **12b** provided 2-mers **14a** (DD) and **14b** (AA). The same sequential deprotection and thiol–ene coupling procedure yielded 3-mers **15a** (DDD) and **15b** (AAA), and 4-mer **16a** (DDDD). Deprotection of the *S*-acetyl group of **15b** resulted in the corresponding disulfide, but reduction with dithiothreitol (DTT) gave the thiol, which was coupled with **12b** to give 4-mer **16b** (AAAA). No epimerization was observed in any of the oligomers.

### Binding studies

^31^P and ^1^H NMR titration experiments in toluene-*d*_8_ were carried out to measure the association constants for length-complementary oligomers. The patterns of chemical shift observed for the oligomers in the free state in toluene are similar, and there is no evidence of any intramolecular folding. The donor oligomers were titrated into the acceptor oligomers, and the titration data fit well to 1 : 1 binding isotherms for all four systems (see ESI for details†). The association constants for the 1-mer and 2-mer complexes (A·D and AA·DD) were determined using both ^1^H and ^31^P NMR titrations, which gave the same results. The ^31^P signals for the 3-mer and 4-mer complexes (AAA·DDD and AAAA·DDDD) were too broad to monitor reliably, so these association constants were determined using ^1^H NMR titrations. The association constants for duplex formation between two oligomers with *N* recognition units (*K*_*N*_) are provided in Table 1 together with the limiting complexation-induced changes in chemical shift, Δ*δ*. The association constant increases by an order of magnitude for every recognition module added to the oligomer, which suggests that all of the recognition modules are involved in cooperative H-bonding interactions in the duplexes. The patterns of the ^31^P and ^1^H NMR complexation-induced change in chemical shift are the same for all four duplexes suggesting that they adopt similar supramolecular motifs. In particular, the large downfield complexation-induced changes in chemical shift observed for the ^31^P NMR signals (+6 ppm) indicate that all of the phosphine oxide groups form H-bonds in the duplexes.^20^

**Table 1:** Association constants (*K*_*N*_), effective molarities (EM) and complexation-induced changes in ^31^P and ^1^H NMR chemical shift (Δ*δ*) for the formation of duplexes in toluene-*d*_8_ at 298 K^*a*^

Complex		log *K*_*N*_/M^–1^	EM/mM	*K* EM	Δ*δ*_^31^P_/ppm	Δ*δ*_^1^H_/ppm^*b*^
A·D	(**12a**·**12b**)	2.7 ± 0.2	—	—	6.4	0.09
AA·DD	(**14a**·**14b**)	4.0 ± 0.2	14 ± 12	8 ± 5	6.4	0.08
AAA·DDD	(**15a**·**15b**)	5.4 ± 0.1	28 ± 16	16 ± 3	6.3	0.09
AAAA·DDDD	(**16a**·**16b**)	6.3 ± 0.1	22 ± 11	12 ± 2	5.9	0.10

^*a*^Each titration was repeated twice and the average value is reported with errors at the 95% confidence limit.

^*b*^Values for the signal due to the protons *ortho* to the phosphine oxide groups.

**Fig. 3:**
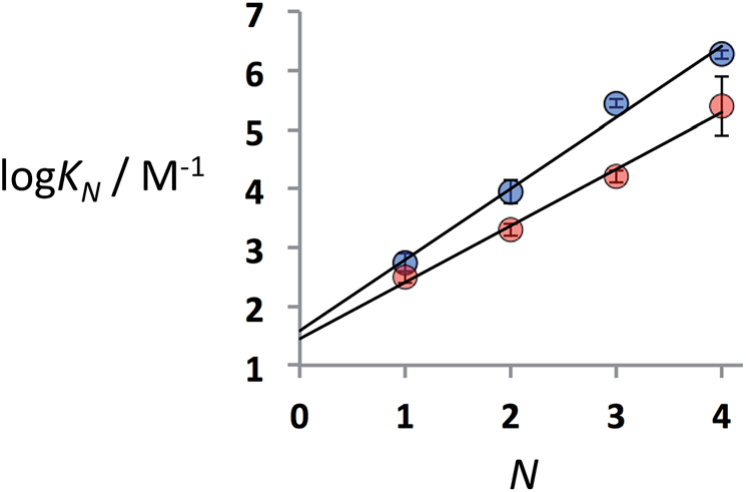
Association constants for duplex formation in toluene-*d*_8_ at 298 K (*K*_*N*_) plotted as a function of the number of recognition modules in the oligomer, *N*. Data shown are for the duplexes obtained using thiol–ene coupling in blue, and in red for the duplexes obtained using reductive amination chemistry (the best fit straight lines are shown, *R* ^2^ = 0.99 and 0.99, respectively).

Fig. 3 shows the logarithm of the association constant for duplex formation (log *K*_*N*_) plotted as a function of the number of H-bonding interactions (*N*). The data fit well to a straight line with a slope of 1.2 (eqn (1)), *i.e.* the association constant increases by an order of magnitude for each additional H-bond formed.

1 log *K*_*N*_ = 1.2*N* + 1.6

Fig. 3 also shows the corresponding data for the duplexes obtained using reductive amination chemistry (Fig. 2a). The reductive amination oligomers have similar phosphine oxide–phenol recognition modules to the thiol–ene system, but the backbone is more rigid. Surprisingly, the thiol–ene duplexes are significantly more stable than the reductive amination duplexes. There are two factors that contribute to the overall stability of a duplex: the intrinsic strength of the H-bonding interactions, which is a property of the recognition modules, and the effective molarity (EM) for formation of intramolecular H-bonds in zipping up of the duplex, which is a property of the backbone. The association constant for formation of a duplex, which is *N* recognition modules long (*K*_*N*_), can be expressed using eqn (2).

2 *K*_*N*_ = 2*K*^*N*^EM^*N*–1^

where *K* is the association constant for the formation of the corresponding intermolecular H-bond in the A·D complex (**12a**·**12b**) and EM is the average effective molarity for the formation of intramolecular H-bonds.

The values of EM for the thiol–ene duplexes are reported in Table 1. The results are similar for all three duplexes that form intramolecular interactions, which implies that the backbone is sufficiently flexible to optimise the H-bond geometries in all of these oligomers. Once the first intermolecular H-bond between the two strands is formed, all subsequent intramolecular H-bonds have an average EM of 18 mM. The equilibrium constant for forming an intramolecular H-bond in a partially assembled duplex is given by the product *K* EM, which provides a measure of the probability of zipping up the duplex as opposed to forming intermolecular interactions that would lead to polymeric aggregates.^21^ For the thiol–ene duplexes, the values of *K* EM in Table 1 are all significantly greater than one (the average value is 12), implying that intramolecular H-bonding is highly favoured in these systems. For the reductive amination duplexes shown in Fig. 2a, the average EM is 14 mM and the average value of *K* EM is 5. Surprisingly, the flexible thiol–ene backbone leads to a slightly higher EM than was found for the more rigid reductive amination backbone. The differences between the properties of the thiol–ene and reductive amination duplexes are small (*K* = 560 and 350 M^–1^, and EM = 18 and 14 mM, respectively), but these subtle differences are multiplied along the length of an oligomer, so that the stability of the thiol–ene 4-mer duplex is an order of magnitude larger than the stability of the reductive amination 4-mer duplex.

### Thermal denaturation studies

**Fig. 4:**
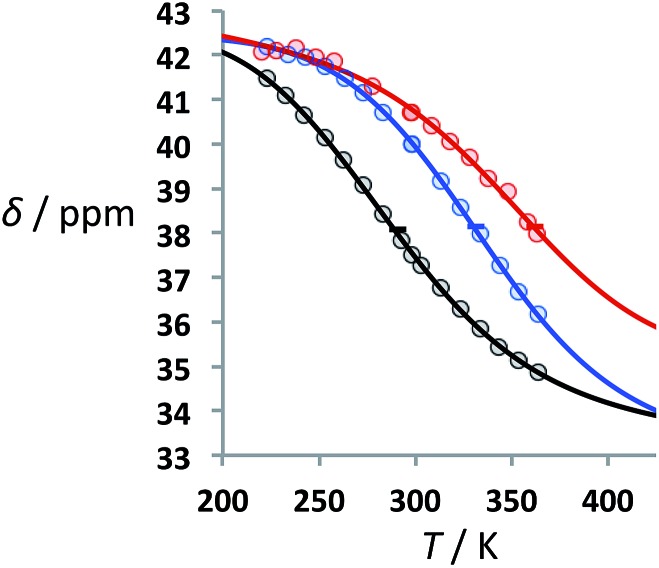
Experimental ^31^P NMR chemical shift plotted as a function of temperature for 1 : 1 mixtures (1 mM) of A·D (black), AA·DD (blue), and AAA·DDD (red) in toluene-*d*_8_. The lines are the best fit to eqn (4) (total rmsd < 0.2 ppm). The horizontal bars show the transition melting temperatures, *T*_m,*N*_.

Thermal denaturation experiments were carried out to extract the thermodynamic parameters for duplex assembly. ^31^P NMR spectra of 1 : 1 solutions of length-complementary oligomers at 1 mM concentrations in toluene-*d*_8_ were recorded at different temperatures between 223 and 363 K. The ^31^P NMR signals shifted progressively downfield at low temperatures, indicative of an increase in the population of H-bonded complexes. At high temperatures, an upfield shift in the ^31^P NMR signals was observed consistent with disruption of the H-bonding interactions between oligomers (Fig. 4).

The data obtained for the AAAA·DDDD duplex (**16a**·**16b**) was qualitatively consistent with a duplex that melts at a higher temperature than the AAA·DDD duplex (**15a**·**15b**), but it was not possible to extract an accurate melting profile for the 4-mer because the signals were broad and overlapped. Thermodynamic parameters for the other three duplexes were obtained from the melting profiles by fitting the data to a two-state model, assuming that only duplex and denatured single strands are present, that the enthalpy and entropy changes for the formation of the *N*-mer duplex (
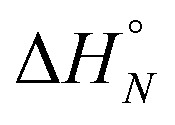
 and 
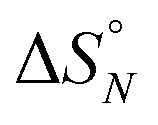
) are temperature independent, and that the change in heat capacity between free and bound states is zero.^22^ The equilibrium constant for duplex formation at a given temperature *T*, *K*_*N*_(*T*), is given by eqn (3), which can be derived from the van't Hoff equation.^10^
3
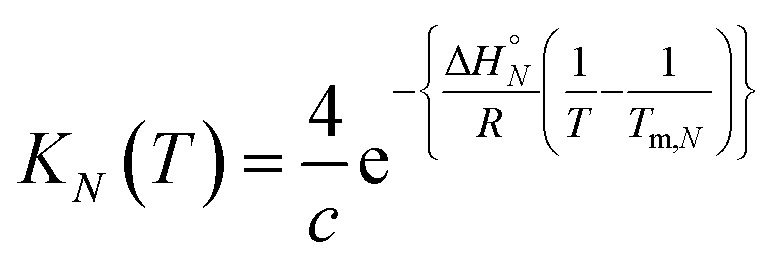
where *T*_m,*N*_ is the transition melting temperature for the *N*-mer duplex, and *c* is the total concentration of the two oligomers, which are present in equal concentrations (*c*/2).

The observed chemical shift (*δ*) in the NMR denaturation experiment can be expressed as a function of *T* using eqn (4).^10^
4
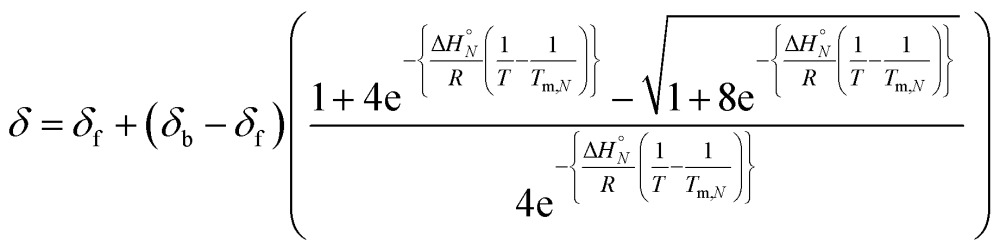
where *δ*_f_ and *δ*_b_ correspond to the chemical shifts of the single strand and duplex states, respectively.

Fig. 4 shows the best fit of eqn (4) to the experimental melting data. The optimised values of *δ*_f_ and *δ*_b_ obtained from fitting the melting data agree with the values determined in the NMR titration experiments carried out at 298 K (*δ*_f_ ≈ 35–36 ppm and *δ*_b_ ≈ 41–42 ppm).

Table 2 shows the values of the parameters 
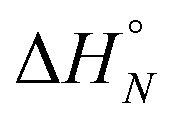
 and *T*_m,*N*_ obtained from the thermal denaturation experiments, along with the values of log *K*_*N*_ calculated at 298 K using eqn (3). The values of log *K*_*N*_ at 298 K determined from the thermal denaturation experiments are similar to the corresponding values determined in the 298 K titration experiments (Table 1), indicating that the assumptions used in the treatment of the thermal denaturation data are valid. At room temperature, an increase of one order of magnitude in *K*_*N*_ is observed for each additional H-bond formed. There is a corresponding increase in the transition melting temperature and the enthalpy change on duplex formation with increasing numbers of H-bonds. These observations are indicative of cooperative H-bonding interactions along the duplex. We also note that the values of *T*_m,*N*_ are consistently 10–20 degrees higher than observed for the corresponding reductive amination duplexes, confirming the enhanced stability of the more flexible thiol–ene systems.

**Table 2:** Thermodynamic parameters for the formation of H-bonded duplexes in toluene-*d*_8_ determined using ^31^P NMR thermal denaturation experiments

Complex		*T*_m,*N*_/K	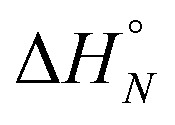 /kJ mol^–1^	log *K*_*N*_(298)/M^–1^	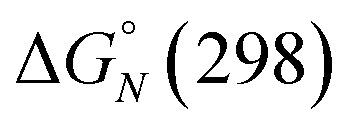 /kJ mol^–1^	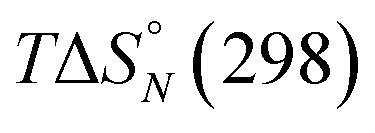 /kJ mol^–1^
A·D	(**12a**·**12b**)	285	–26	3.4	–19	–7
AA·DD	(**14a**·**14b**)	334	–39	4.3	–25	–14
AAA·DDD	(**15a**·**15b**)	365	–46	5.1	–29	–17

### Molecular modelling

**Fig. 5:**
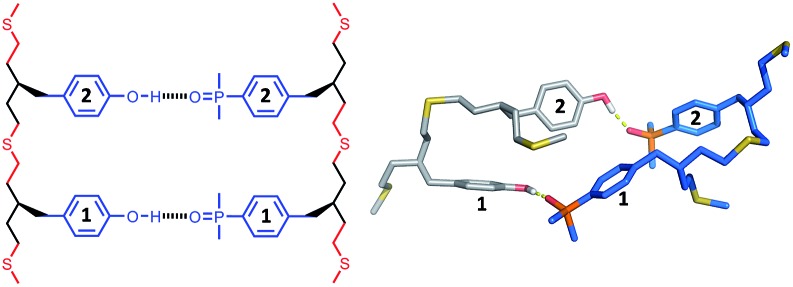
Lowest energy conformation of the 2-mer duplex AA·DD from a conformational search (MMFFs force-field and CHCl_3_ solvation implemented in Macromodel).^23^ The H-bond donor oligomer is shown in grey and the H-bond acceptor oligomer in blue. Hydrogens are not shown for clarity, and the recognition modules are numbered.

**Fig. 6:**
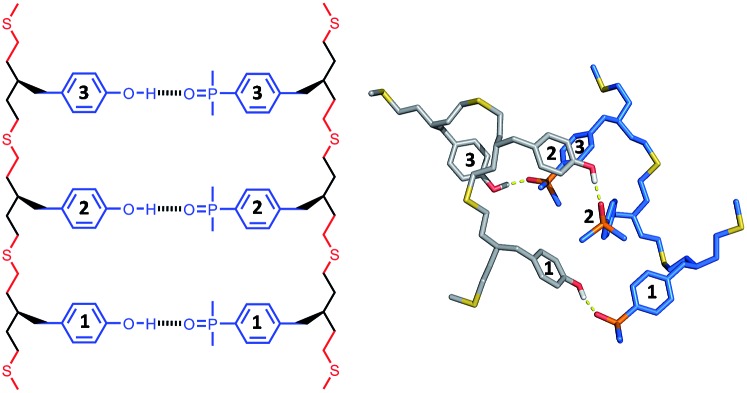
Lowest energy conformation of the 3-mer duplex AAA·DDD from a conformational search (MMFFs force-field and CHCl_3_ solvation implemented in Macromodel).^23^ The H-bond donor oligomer is shown in grey and the H-bond acceptor oligomer in blue. Hydrogens are not shown for clarity, and the recognition modules are numbered.

**Fig. 7:**
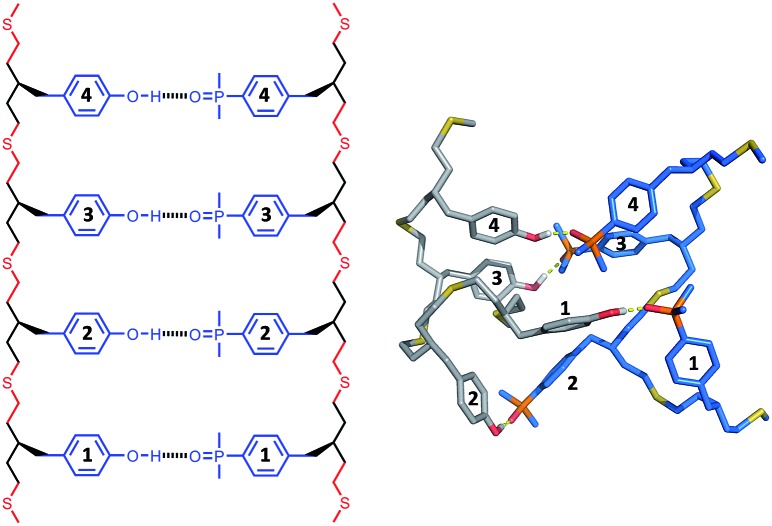
Lowest energy conformation of the 4-mer duplex AAAA·DDDD from a conformational search (MMFFs force-field and CHCl_3_ solvation implemented in Macromodel).^23^ The H-bond donor oligomer is shown in grey and the H-bond acceptor oligomer in blue. Hydrogens are not shown for clarity, and the recognition modules are numbered.

The structures of the duplexes were investigated using molecular mechanic calculations. The first H-bond in the duplex was fixed by constraining the distance between the phenol hydrogen and the phosphine oxide oxygen to 2 ± 1 Å. A conformational search was used to find low energy conformations compatible with this single point constraint (see ESI† for details). Fig. 5–7 show the lowest energy structures obtained from conformational searches for AA·DD, AAA·DDD and AAAA·DDDD, respectively. The fully assembled duplex was found in all cases with all of the recognition modules on one oligomer H-bonded to the complementary sites on the other oligomer. It is clear from these calculations that the thiol–ene oligomers are very flexible: the calculated structures shown in Fig. 5–7 are collapsed, unlike the idealised ChemDraw structures. Nevertheless, it appears that there is good fidelity in the pairing of the recognition modules, because complexes with out of register H-bonding interactions were not observed in the calculations: for example for the 3-mer duplex, out of register would be phenols 2 and 3 paired with phosphine oxides 3 and 2, as opposed to the in register arrangement shown in Fig. 6, which has phenols 2 and 3 paired with phosphine oxides 2 and 3.

**Fig. 8:**
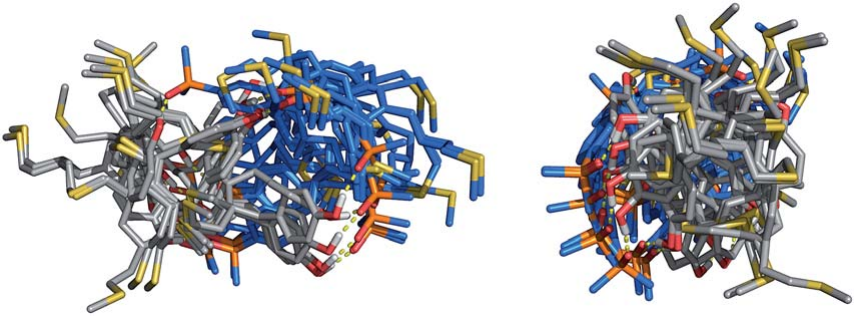
Superposition of 11 different conformations that were found within 5 kJ mol^–1^ of the global minimum for the 2-mer AA·DD duplex. Two different points of views are shown. The H-bond donor oligomers are shown in grey and the H-bond acceptor oligomers in blue, and hydrogens are not shown.

**Fig. 9:**
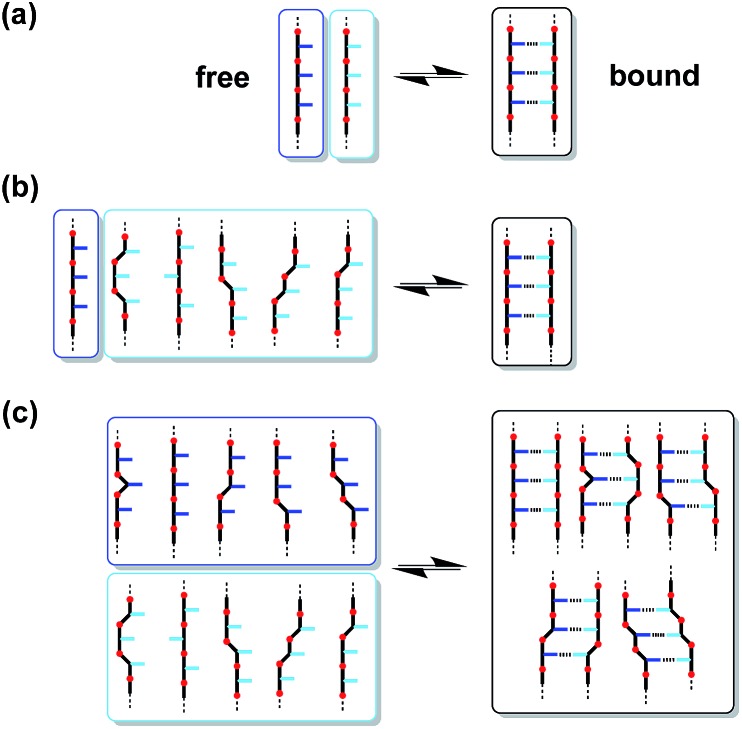
The effect of conformational flexibility on duplex stability. (a) For two rigid oligomers, the duplex has similar conformational properties to the free state. (b) For a flexible oligomer (pale blue) binding to a rigid oligomer (dark blue), duplex formation significantly reduces the number of conformational states. (c) For two flexible oligomers, the duplex retains much of the conformational flexibility present in the free state.

The conformational flexibility of the thiol–ene duplexes provides a possible explanation for the unusually high stability exhibited by these systems. Fig. 8 shows an overlay of low energy duplex structures that were found for the 2-mer AA·DD: a wide range of different conformations is compatible with duplex formation. If most of the conformational states accessible to the single stranded oligomers are also available to the assembled duplex then the entropic cost restricting of degrees of freedom will be small. Fig. 9 illustrates how the population of conformations in the free and bound states contributes to the free energy of duplex formation in rigid and flexible systems. A large entropic penalty is expected for duplexes formed between a rigid and a flexible backbone, but if both of the backbones are rigid or if both of the backbones are flexible, the duplex should be relatively stable, because the change in the number of accessible conformations is relatively small. In other words, conformational flexibility is only detrimental to binding if conformational degrees of freedom are lost on complexation.

## Conclusions

A new class of oligomeric molecules bearing phosphine oxide and phenol recognition modules is reported. These systems were prepared using iterative thiol–ene coupling chemistry to produce the all H-bond donor and the all H-bond acceptor oligomers up to four repeats long. The monomers for oligomer synthesis were prepared in homochiral form with a terminal alkene and a protected thiol, and no epimerisation was observed in subsequent reactions. ^1^H and ^31^P NMR titrations and thermal denaturation experiments demonstrate that length-complementary donor and acceptor oligomers form H-bonded duplexes in toluene. The association constant for duplex formation increases uniformly by an order of magnitude for each additional recognition module added to the chain, indicating that all of the phosphine oxide and phenol groups are involved in H-bonding interactions in the duplexes. Thermal denaturation experiments also show that there is an increase in the enthalpy change for duplex formation as the length of the oligomer increases, demonstrating cooperative H-bonding interactions along the duplex. The properties of the thiol–ene oligomers can be compared with a different set of oligomers that we have previously reported which were assembled using reductive amination chemistry. Both systems use phenol–phosphine oxide H-bonding interactions as the recognition motif, but they differ significantly in the flexibility of the backbone. The thiol–ene oligomers have a highly flexible alkyl chain for the backbone, whereas the reductive amination oligomers have a more rigid backbone featuring aromatic units. Surprisingly, the more flexible thiol–ene oligomers form the more stable duplexes: the thiol–ene AAAA·DDDD duplex is an order of magnitude more stable than the corresponding reductive amination duplex. Molecular modelling studies suggest that the thiol–ene duplexes can access a large number of different conformations in the bound state, and so the flexibility of the backbone is not significantly restricted on binding in this system. We have previously reported that small differences in backbone flexibility have a minimal effect on the EM for duplex formation using three different reductive amination oligomers (7–20 mM). The values of EM for duplex formation with the thiol–ene oligomers are 14–28 mM, which shows that much more dramatic increases in backbone flexibility have almost no impact on the EM for intramolecular H-bond formation. The results demonstrate that conformationally flexible molecules can form high affinity complexes provided that conformational mobility is retained in the bound state.
