# Supplementary material for: Homochiral oligomers with highly flexible backbones form stable H-bonded duplexes
Source: Chem Sci. 2016 Aug 19;8(1):206–13. doi: 10.1039/c6sc02995g (PMC5308278; doi:10.1039/c6sc02995g)

Electronic Supplementary Material (ESI) for Chemical Science.
This journal is © The Royal Society of Chemistry 2016

Supporting information

HOMOCHIRAL OLIGOMERS WITH HIGHLY FLEXIBLE BACKBONES FORM STABLE H-BONDED DUPLEXES

Diego Núñez-Villanueva[a], Christopher A. Hunter*[a]

[a]*Department of Chemistry, University of Cambridge, Lensfield Road, Cambridge CB2 1EW, UK. E-mail: herchelsmith.orgchem@ch.cam.ac.uk*

TABLE OF CONTENTS **Page**

NMR titrations S2

Thermal denaturation experiments S5

Molecular mechanic calculations S7

Synthesis of compound 1-16 and NMR spectra S8

General experimental details S8

Synthesis of **2** S9

Synthesis of **3a** S12

Synthesis of **5** S15

Synthesis of **3b** S18

Synthesis of **7** S22

General procedure for the synthesis of compounds **8a** and **8b** S25

General procedure for the synthesis of compounds **9a** and **9b** S32

General procedure for the synthesis of **10a** and **10b** S39

General procedure for the synthesis of **11a** and **12b** S47

Synthesis of compound **12a** S54

General procedure for the synthesis of compounds **13a** and **13b** S57

General procedure for the synthesis of oligomers **14-16** S64

NMR titrations

All binding constants were measured by ^{31}P and ^{1}H NMR titrations in a Bruker 500 MHz AVIII HD Smart Probe or TCI Cryoprobe spectrometers. The host (phosphine oxide derivatives **12b**, **14b**, **15b** or **16b**) was dissolved in toluene-d_8 at a known concentration. The guest (phenol derivatives **12a**, **14a**, **15a** or **16a**) was dissolved in the host solution and made to a known concentration. A known volume of host was added to an NMR tube and the spectrum was recorded. Known volumes of guest in host solution were added to the NMR tube, and the spectra were recorded after each addition. The chemical shifts of the host spectra were monitored as a function of guest concentration and analysed using a purpose written software in Microsoft Excel. Errors were calculated as two times the standard deviation from the average value (95% confidence limit).

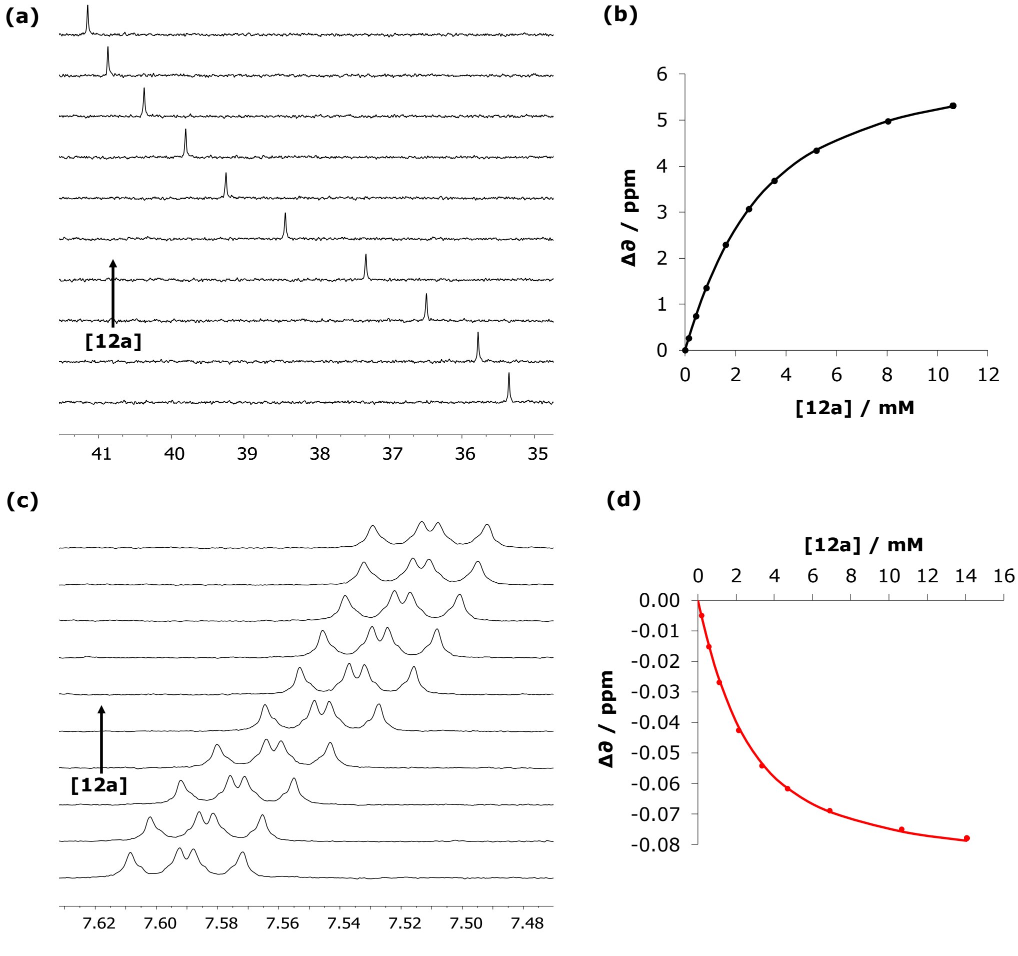

Figure S1. NMR data for titration of **12a** into **12b** (1.57 mM) at 298 K in toluene-d_8. (a) and (c) 202 MHz ^{31}P NMR spectra and 500 MHz ^{1}H NMR spectra of the phosphine oxide aromatic α-protons, respectively. (b) and (d) Plot of the change in chemical shift of the ^{31}P and ^{1}H signal, respectively, as a function of guest concentration (the line represents the best fit to a 1:1 binding isotherm).

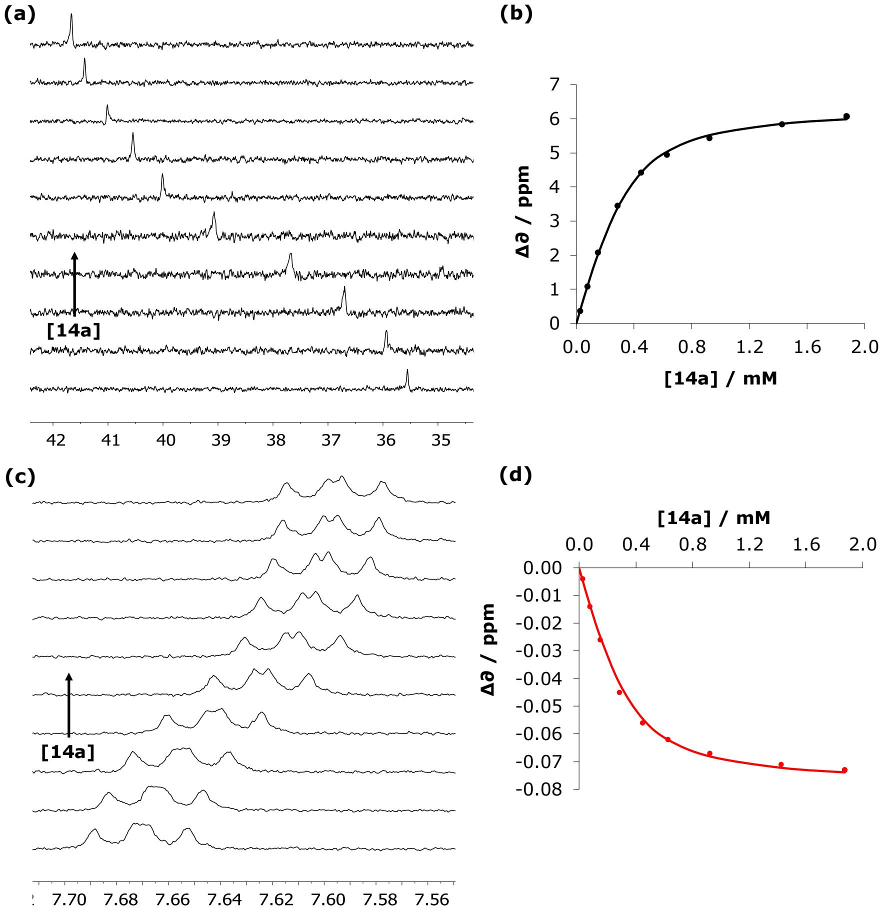

Figure S2. NMR data for titration of **14a** into **14b** (0.33 mM) at 298 K in toluene-d_8. (a) and (c) 202 MHz ^{31}P NMR spectra and 500 MHz ^{1}H NMR spectra of the phosphine oxide aromatic α-protons, respectively. (b) and (d) Plot of the change in chemical shift of the ^{31}P and ^{1}H signal, respectively, as a function of guest concentration (the line represents the best fit to a 1:1 binding isotherm).

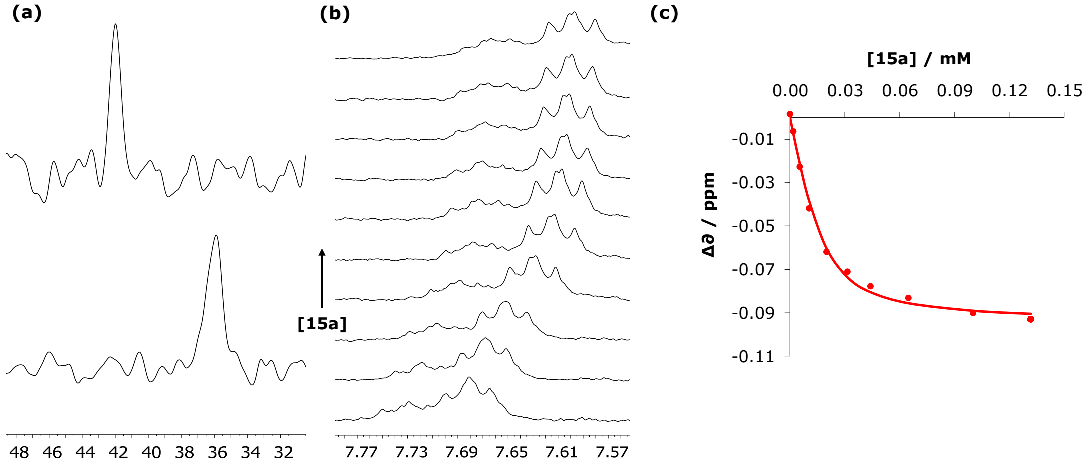

Figure S3. NMR data for titration of **15a** into **15b** (0.02 mM) at 298 K in toluene-d_8. (a) 202 MHz ^{31}P NMR of the free (bottom) and fully bound (top) host. (b) 500 MHz ^{1}H NMR spectra of the phosphine oxide aromatic α-protons. (c) Plot of the change in chemical shift of the ^{1}H signal as a function of guest concentration (the line represents the best fit to a 1:1 binding isotherm). A 10 Hz line broadening was applied to the spectra shown in (b) in order to extract chemical shift information.

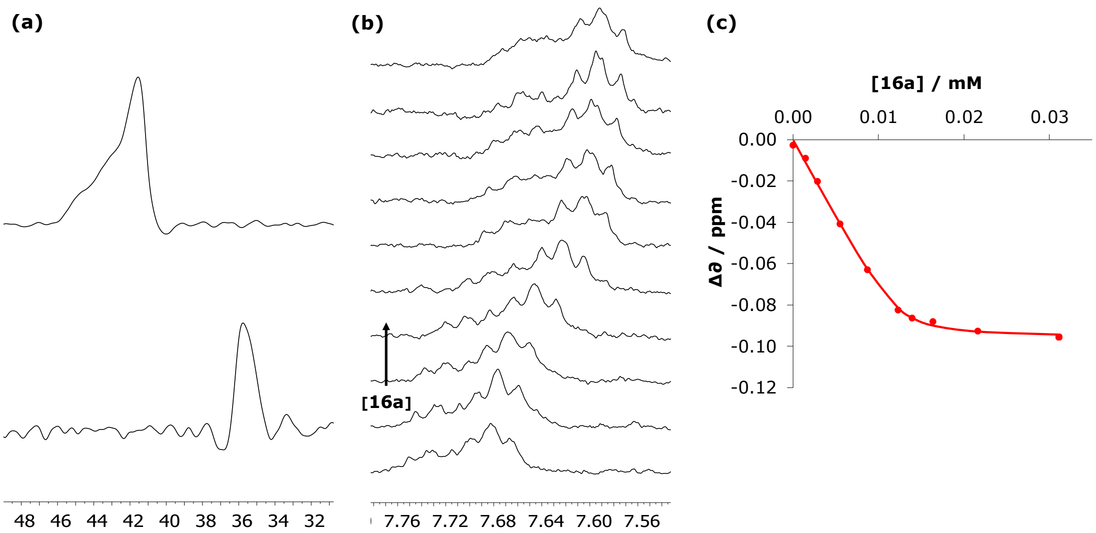

Figure S4. NMR data for titration of **16a** into **16b** (0.01 mM) at 298 K in toluene-d_8. (a) 202 MHz ^{31}P NMR of the free (bottom) and fully bound (top) host. (b) 500 MHz ^{1}H NMR spectra of the phosphine oxide aromatic α-protons. (c) Plot of the change in chemical shift of the ^{1}H signal as a function of guest concentration (the line represents the best fit to a 1:1 binding isotherm). A 10 Hz line broadening was applied to the spectra shown in (b) in order to extract chemical shift information.

Table S1. ^{31}P and ^{1}H (phosphine oxide aromatic α-protons) NMR chemical shifts and limiting complexation-induced changes in chemical shifts of the free host (ppm) obtained by fitting titration data measured in toluene-d_8 at 298 K to a 1:1 binding isotherm.

		**^{31}P NMR**			**^{1}H NMR**		
**Complex**		**δ_{free}**	**δ_{bound}**	**Δδ**	**δ_{free}**	**δ_{bound}**	**Δδ**
A•D	(**12a•12b**)	35.4	41.8	6.4	7.59	7.50	-0.09
AA•DD	(**14a•14b**)	35.6	42.0	6.4	7.67	7.59	-0.08
AAA•DDD	(**15a•15b**)	35.9	42.2	6.3	7.69	7.60	-0.09
AAAA•DDDD	(**16a•16b**)	35.8	41.7	5.9	7.68	7.59	-0.10

Thermal denaturation experiments

Thermal denaturation data were measured by making 1:1 solutions of length-complementary oligomers at 1 mM concentrations in toluene-d_8 and measuring ^{31}P NMR spectra at different temperatures in a Bruker 500 MHz AVIII HD Smart Probe spectrometer equipped with a BCU Chiller unit. The temperature of the sample was changed using the internal thermostat of the NMR spectrometer, and the sample was allowed to equilibrate in the probe until the probe thermometer gave a stable temperature. Due to significant broadening of the ^{31}P NMR peaks for the AAA•DDD (**15a•15b**) and AAAA•DDDD duplexes (**16a•16b**), 50 Hz line broadening was applied to all spectra (Figure S5c and S5d). The data obtained for the AAAA•DDDD duplex (**16a•16b**) is qualitative consistent with a duplex that melts at higher temperatures than the AAA•DDD duplex. However, it was not possible to extract accurate thermodynamic parameters for the formation of duplexes because the signals become very broad and overlapped with changes in temperature. In addition, at low temperature the melting profile suggests the existence of secondary processes like aggregation).

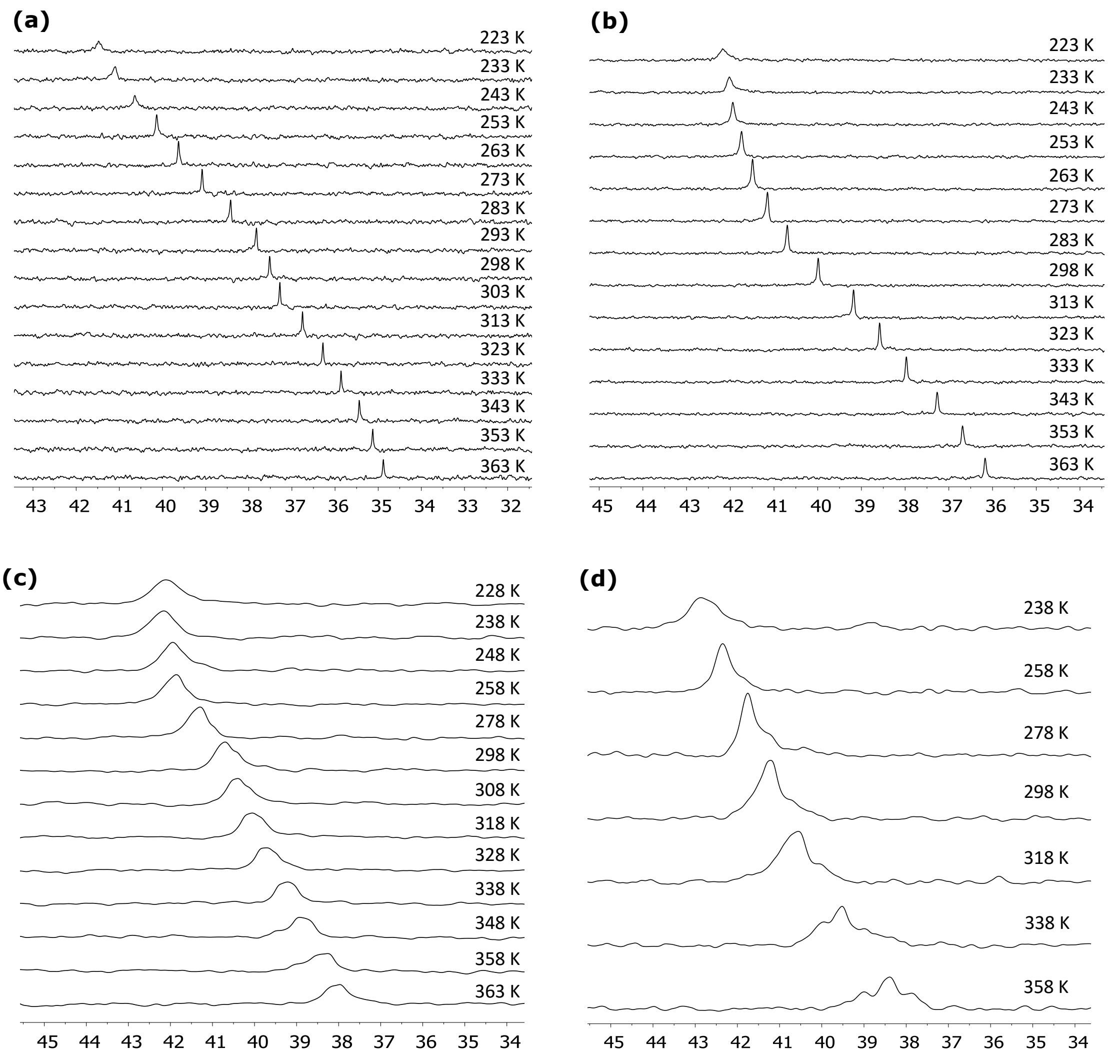

Figure S5. Variable temperature 202 MHz ^{31}P NMR spectra in toluene-d_8 for 1 mM solutions of 1:1 mixtures of: (a) **12a•12b**. (b) **14a•14b**. (c) **15a•15b**. (d) **16a•16b**. A line broadening of 50 Hz was applied to the spectra in (c) and (d).

Molecular mechanic calculations

Molecular mechanic calculations were performed using MacroModel version 9.8 (Schrödinger Inc.) on simplified AA•DD, AAA•DDD and AAAA•DDDD duplexes in which the *n*-hexyl solubilising group, the *n*-butyl groups in the phosphine oxide and the acetyl group were changed to methyl groups in order to reduce the computational cost. All structures were minimized first and the minimized structures were then used as the starting molecular structures for all MacroModel conformational searches. The force field used was MMFFs as implemented in this software. The charges were defined by the force field library and no cut off were used for non-covalent interaction. For the study of AA•DD and AAA•DDD duplex structures, one of the terminal H-bonds was fixed by constraining the distance between the phenol hydrogen and phosphine oxide oxygen to 2 ± 1 Å. In the case of the AAAA•DDDD duplex, the first and the third H-bonds were fixed using the same parameters as before. A Polak-Ribiere Conjugate Gradient (PRCG) was used and each structure was subjected to 10000 iterations. The minima converged on a gradient with a threshold of 0.01. Conformational search was performed from previously minimized structures using 10000 steps. Only the structures in a 5 kJ·mol^{-1} window from the global minimum were analysed. Images shown in Fig. 5-8 were created using PyMol.[S1]

(S1) The PyMOL Molecular Graphics System, Version 1.6 Schrödinger, LLC.

Synthesis of compounds 1-16 and NMR spectra

General experimental details

All the reagents and materials used in the synthesis of the compounds described below were bought from commercial sources, without prior purification. UV irradiations were performed using an UVP lamp model UVGL-58 (1x365 nm tube, 6 watt) and an UVP lamp model UVL-28 (2x365 nm tubes, 8 watt). Thin layer chromatography was carried out using with silica gel 60F (Merck) on aluminium. Flash chromatography was carried out on an automated system (Combiflash Companion, Combiflash Rf+ or Combiflash Rf Lumen) using prepacked cartridges of silica (25µ or 50µ PuriFlash® Columns). All NMR spectroscopy was carried out on a Bruker AVI250, AVI400, DPX400, AVIII400 spectrometer using the residual solvent as the internal standard. All chemical shifts (δ) are quoted in ppm and coupling constants given in Hz. Splitting patterns are given as follows: s (singlet), d (doublet), t (triplet), q (quadruplet), m (multiplet). FT-IR spectra were measured on a PerkinElmer Spectrum 100 or One spectrometer equipped with an ATR cell. Melting points were measured in a Mettler Toledo MP50 Melting Point System. Optical activity was measured in an AA-10 or an Anton Paar (MCP 100) at 589 nm. ES+ was carried out on a Waters LCT-TOF spectrometer or a Waters Xevo G2-S bench top QTOF machine.

Synthesis of 2.

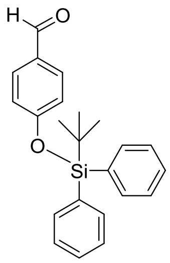

A solution of *p*-hydroxybenzaldehyde (6.00 g, 49.13 mmol) in DMF (60 mL) was treated with imidazol (6.690 g, 98.26 mmol) and TBDPS-Cl (15.117 mL, 58.96 mmol). The solution was stirred at room temperature for 15 h. Then, the mixture was acidified with 0.1 M HCl solution to pH = 2-3 and extracted with EtOAc (3x100 mL). The organic layers were joined and washed with brine and dried over $MgSO_4$. The solvent was evaporated to dryness and the residue was purified by flash chromatography (EtOAc:hexane 1:20) to afford compound **2** (19.030 g, 91%) as a white solid.

Melting point: 106-109 ^{o}C.

^{1}H NMR (400 MHz, CDCl$_3$): δ_H = 9.81 (s, 1H), 7.70 (m, 4H), 7.65 (d, 2H, *J* = 8.5 Hz), 7.44 (m, 2H), 7.38 (m, 4H), 6.86 (d, 2H, *J* = 8.5 Hz), 1.11 (s, 9H).

^{13}C NMR (100.6 MHz, CDCl$_3$): δ_C = 191.0, 161.4, 135.5, 132.1, 131.8, 130.5, 130.4, 128.1, 120.5, 26.5, 19.6.

MS (ES+): m/z (%) = 361.3 $[M+H]^+$.

HRMS (ES+): calcd for $C_{23}H_{25}O_2Si$ 361.1624 $[M+H]^+$, found 361.1618 $[M+H]^+$.

FT-IR (ATR): 2933, 2860, 1699, 1599, 1507, 1271, 1157, 911 ν_{max}/cm^{-1}.

^1H NMR (400 MHz, $CDCl_3$) compound 2

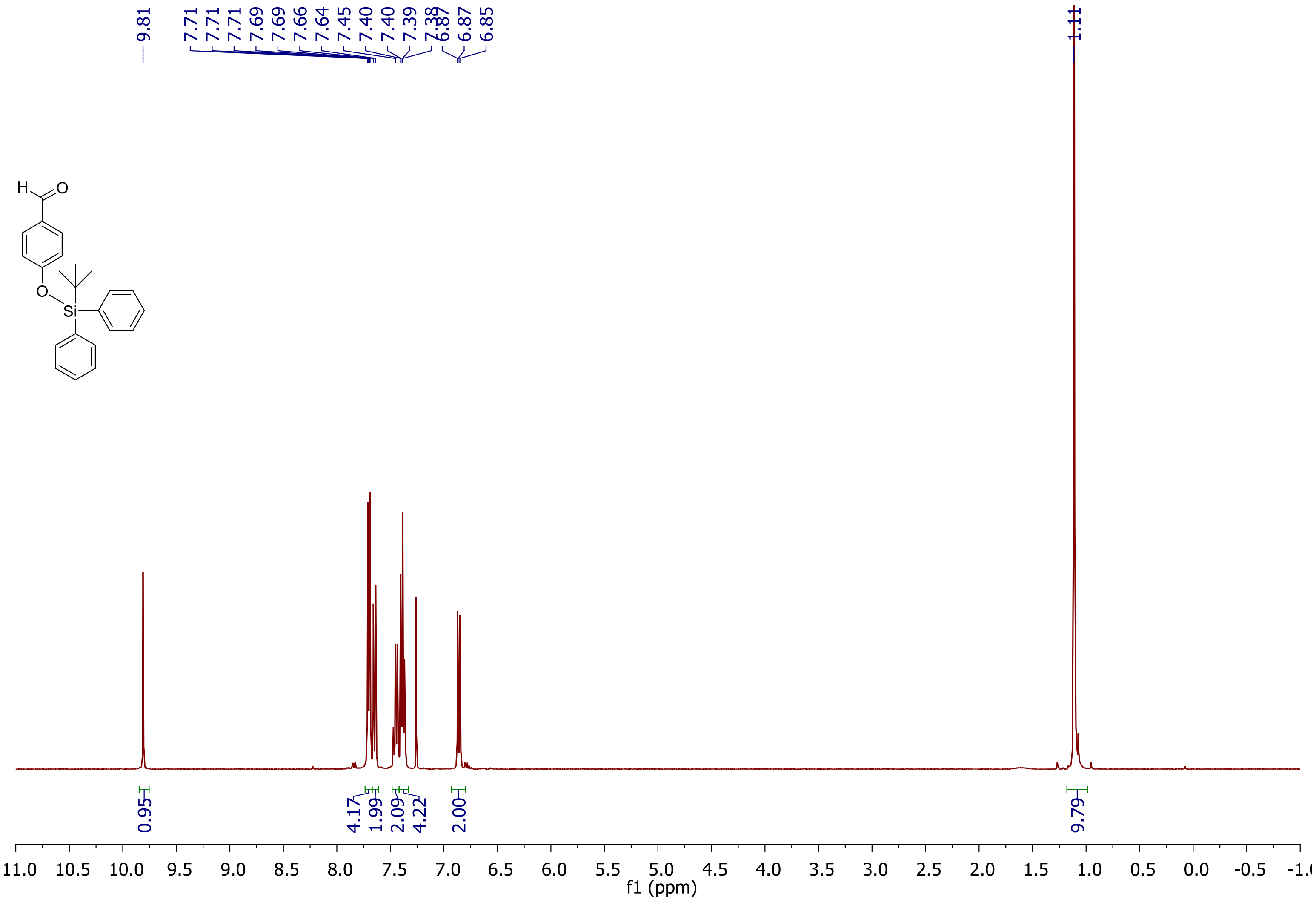

^{13}C NMR (100.6 MHz, $CDCl_3$) compound 2

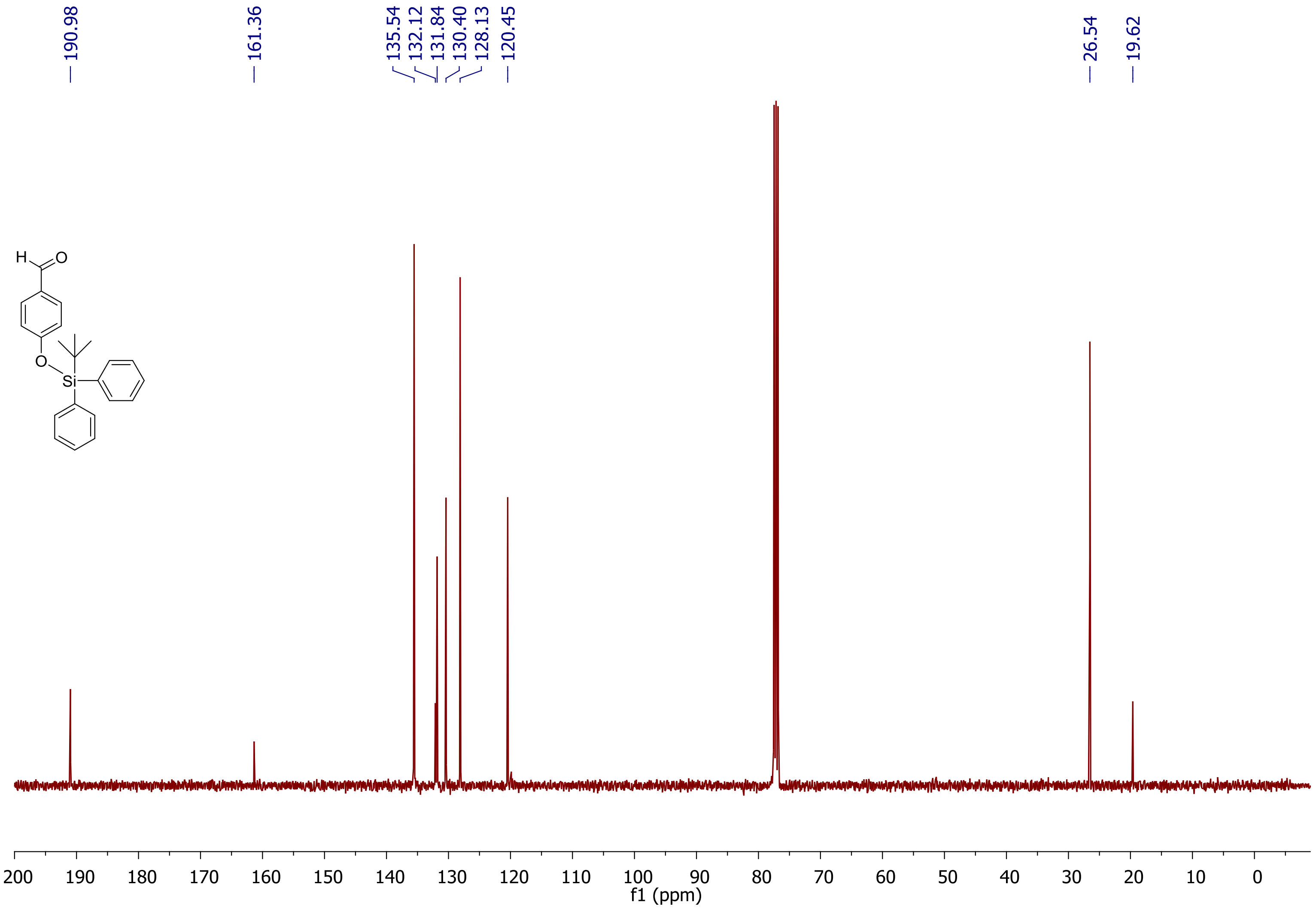

Synthesis of 3a.

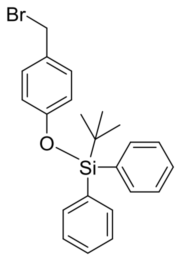

To a solution of aldehyde **2** (13.323 g, 36.95 mmol) in EtOH/Et_2O (1:1, 50 mL) was added $NaBH_4$ (1.538 g, 40.65 mmol) portionwise at 0 °C. After complete addition, the reaction mixture was warmed to room temperature and stirred for 10 min. The reaction was quenched with water (50 mL), extracted with EtOAc (3x100 mL), dried ($MgSO_4$), and concentrated *in vacuo*. To a solution of the obtained benzyl alcohol in CH_2Cl_2 (75 mL) at 0 °C was added a solution of PBr_3 (1.737 mL, 18.48 mmol) in CH_2Cl_2 (50 mL) and the resulting mixture was stirred for 10 min. The reaction mixture was diluted with CH_2Cl_2 (150 mL) and washed with saturated aqueous $NaHCO_3$ solution (2x50 mL) and H_2O (1x50 mL), and then dried ($MgSO_4$) and concentrated to dryness to give the corresponding benzyl bromide compound **3a** (15.093 g, 96%) as a yellow oil.

^{1}H NMR (400 MHz, CDCl$_3$): δ_H = 7.70 (m, 4H), 7.43 (m, 2H), 7.37 (m, 4H), 7.12 (d, 2H, *J* = 8.5 Hz), 6.71 (d, 2H, *J* = 8.5 Hz), 4.43 (s, 2H), 1.09 (s, 9H).

^{13}C NMR (100.6 MHz, CDCl$_3$): δ_C = 155.9, 135.6, 132.7, 130.4, 130.1, 128.0, 120.1, 34.2, 26.6, 19.6.

MS (ES+): m/z (%) = 424.1 $[M+H]^+$.

HRMS (ES+): calcd for $C_{23}H_{25}BrOSi$ 424.0853 $[M+H]^+$, found 424.0855 $[M+H]^+$.

FT-IR (ATR): 2931, 2857, 1606, 1509, 1262, 1113, 916 ν_{max}/cm^{-1}.

^{1}H NMR (400 MHz, $CDCl_3$) compound 3a

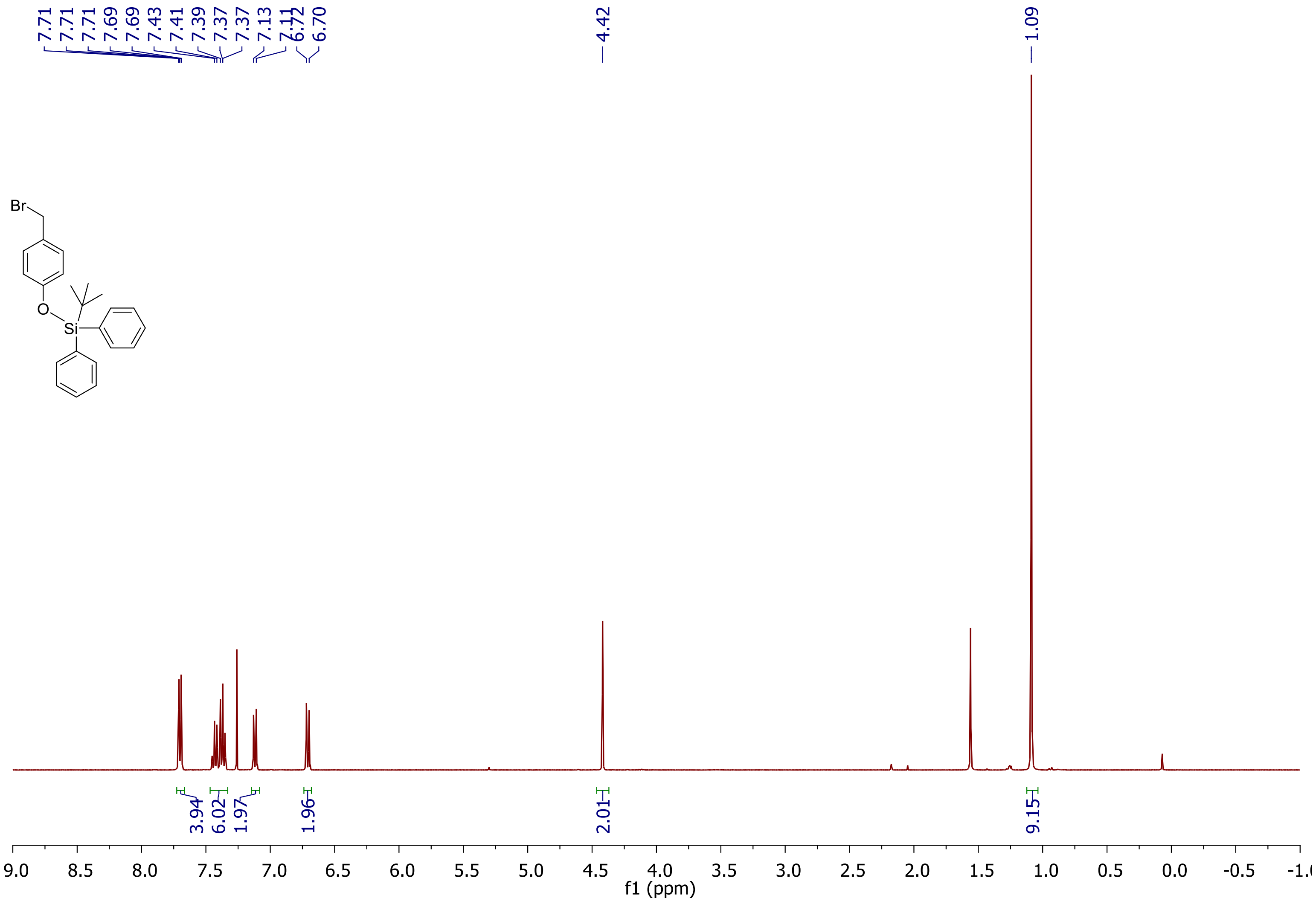

^{13}C NMR (100.6 MHz, $CDCl_3$) compound 3a

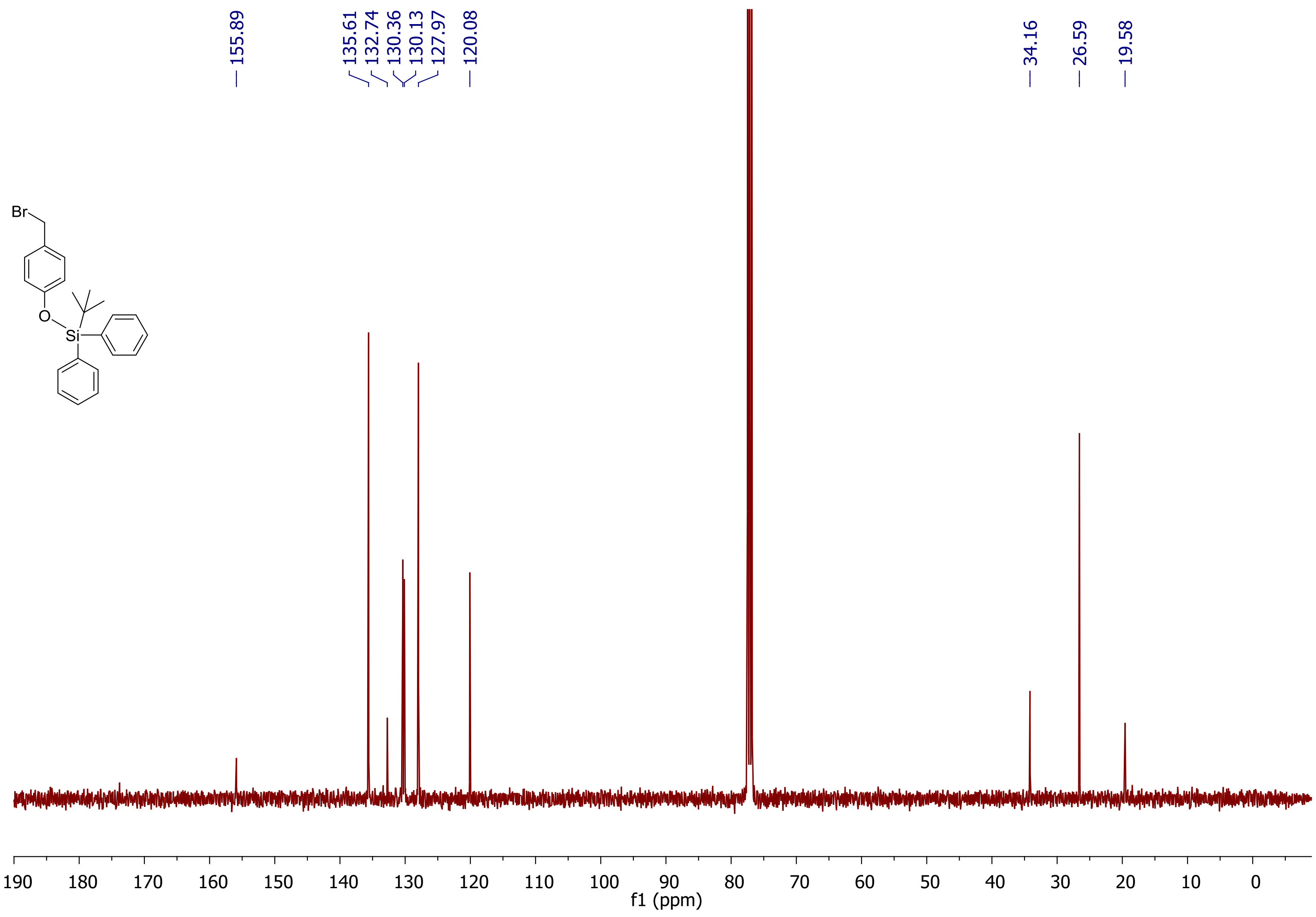

Synthesis of 5.

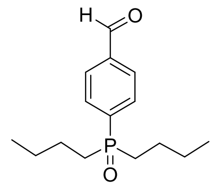

A flask, evacuated/N_2 filled (3x), was charged with *n*-BuMgCl (2 M in Et_2O, 38.78 mL, 77.57 mmol) and the solution cooled to 0°C. A solution of diethylphosphite (3.331 mL, 25.86 mmol) in 50 mL THF was then added dropwise over 15 minutes. The mixture was aged 15 minutes at 0°C, then the bath was removed, and the mixture stirred two hours at room temperature, then cooled again to 0°C. 0.1 M HCl solution (50 mL) was added dropwise over 20 minutes. To the obtained gel was added CH_2Cl_2 (50 mL), and the mixture agitated well for 5 minutes. The resultant mixture was then filtered through a Celite pad, washing the pad with CH_2Cl_2. The filtrate phases were separated, and the organic phase combined with the first organic phase, dried ($MgSO_4$), and the solvents removed *in vacuo*. The residue was azeotroped with hexane (2x30 mL) causing a precipitate that correspond to dibutylphosphine oxide (4.195 g, quantitative). This compound was used in the following step without further purification.

A neat mixture of di-*n*-butylphosphine oxide (4.195 g, 25.86 mmol) and *p*-iodobenzaldehyde (5.000 g, 21.55 mmol) was prepared in a two-neck flask. The flask was then evacuated and refilled with nitrogen three times. Under an atmosphere of nitrogen, 1,4-dioxane (degassed by three freeze-pump-thaw cycles and stored under nitrogen, 50 mL) was added, and the resulting mixture was stirred at room temperature for 10 min. In a separate flask, a dry mixture of tris(dibenzylideneacetone)dipalladium (0.197 g, 0.215 mmol) and Xantphos (0.125 g, 0.215 mmol) was dissolved in 1,4-dioxane (50 mL). Triethylamine (3.514 mL, 25.21 mmol) was added and the resulting mixture was stirred at room temperature for 2 h. Then, the reaction was diluted with CH_2Cl_2 (50 mL) and transferred to a separation funnel. The organic layer was washed with saturated aqueous $NaHCO_3$ solution (2x25 mL). The aqueous layer was extracted with CH_2Cl_2 (3×25 mL). The organic layers were combined and dried over $MgSO_4$. The crude was purified by flash chromatography (EtOAc:MeOH 99:1) to yield compound **5** (5.530 g, 96%) as a foam.

^{1}H NMR (400 MHz, $CDCl_3$): δ_H = 10.09 (s, 1H), 7.99 (m, 2H), 7.88 (m, 2H), 1.93 (m, 4H), 1.58 (m, 2H), 1.38 (m, 6H), 0.86 (t, 6H, *J* = 7.0 Hz).

^{13}C NMR (100.6 MHz, $CDCl_3$): δ_C = 191.8, 140.0 (d, *J* = 86.5 Hz), 138.5 (d, *J* = 2.5 Hz), 131.3 (d, *J* = 9.0 Hz), 129.6 (d, *J* = 11.0 Hz), 29.8 (d, *J* = 68.5 Hz), 24.2 (d, *J* = 14.5 Hz), 23.6 (d, *J* = 4.0 Hz), 13.7.

MS (ES+): m/z (%) = 267.1 $[M+H]^+$.

HRMS (ES+): calcd for $C_{15}H_{24}O_2P$ 267.1514 $[M+H]^+$, found 267.1520 $[M+H]^+$.

FT-IR (ATR): 2958, 2930, 2871, 1698, 1273, 1143, 1098, 758 ν_{max}/cm^{-1}.

^{1}H NMR (400 MHz, $CDCl_3$) compound 5

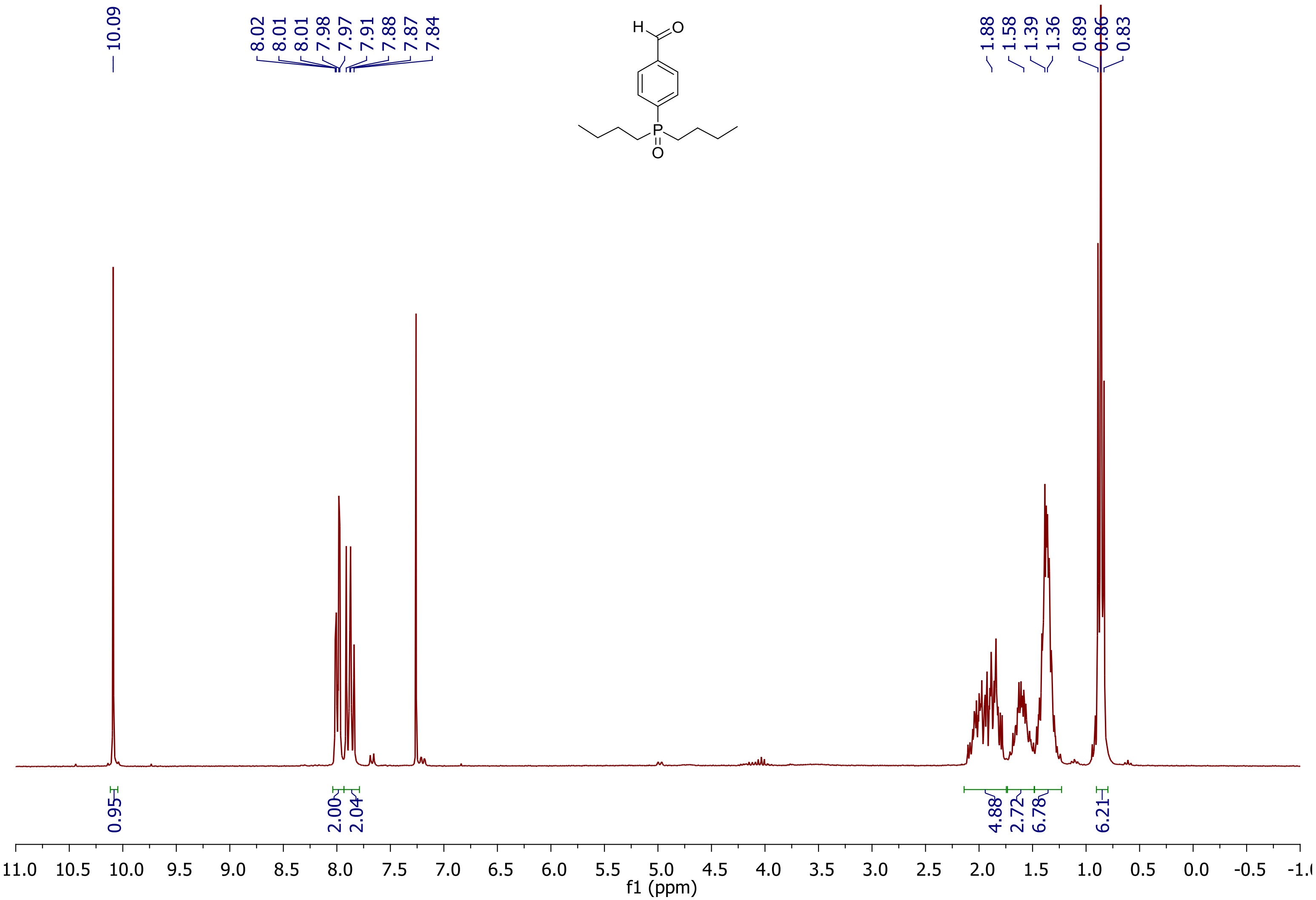

^{13}C NMR (100.6 MHz, $CDCl_3$) compound 5

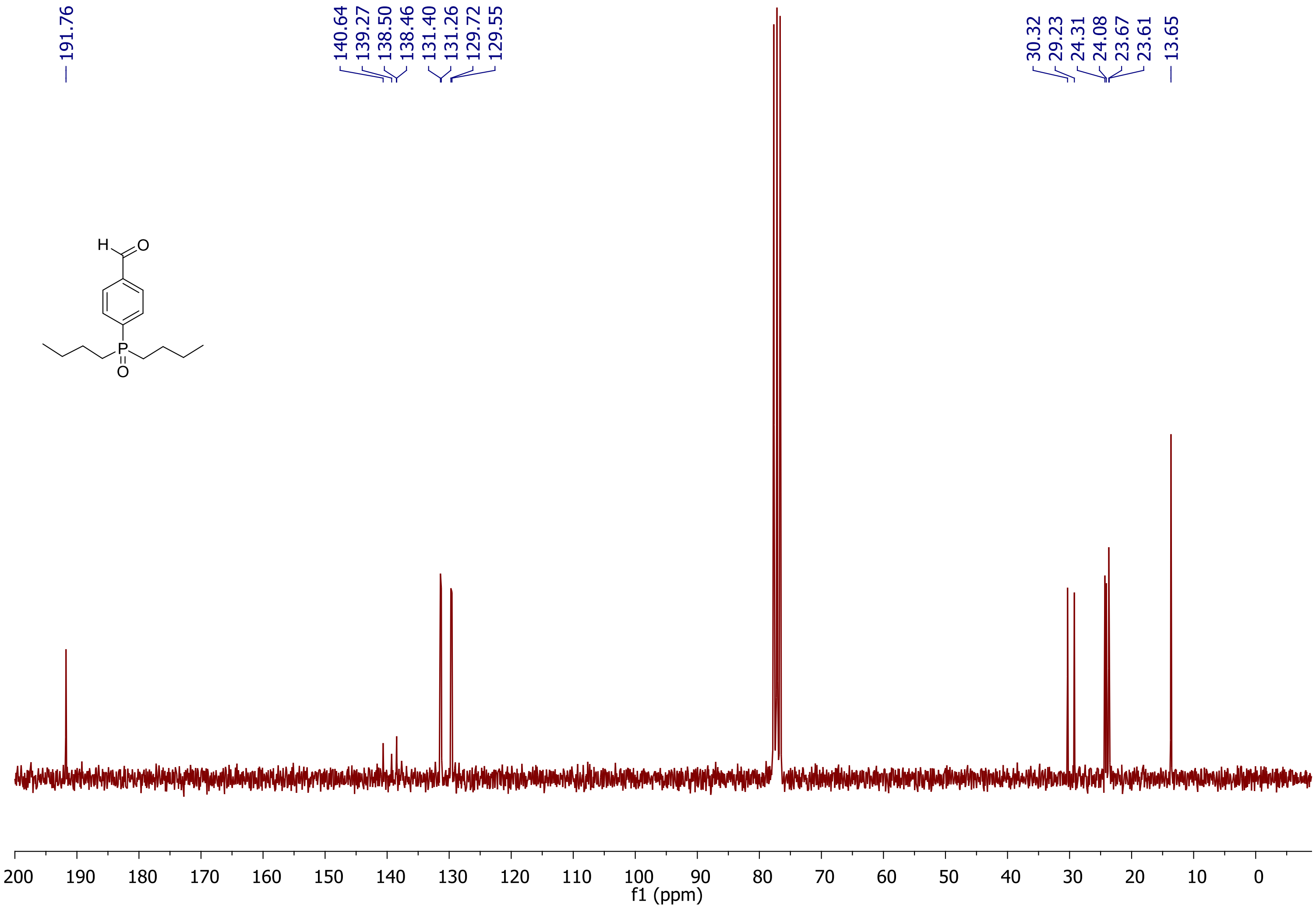

Synthesis of 3b.

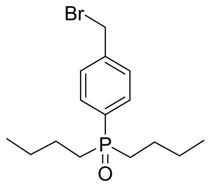

To a solution of aldehyde **5** (5.53 g, 20.76 mmol) in EtOH/Et_2O (1:1, 100 mL) was added $NaBH_4$ (0.864 g, 22.84 mmol) portionwise at 0 °C. After complete addition, the reaction mixture was warmed to room temperature and stirred for 10 min. The reaction was quenched with water, extracted with EtOAc (3x100 mL), dried ($MgSO_4$), and concentrated *in vacuo*. Following this procedure, the corresponding benzyl alcohol (5.48 g, 98%) was obtained as a yellow oil. To a solution of the obtained benzyl alcohol (5.25 g, 19.57 mmol) in CH_2Cl_2 (80 mL) under N_2 atmosphere was added a solution of PPh_3 (9.23 g, 35.22 mmol) in CH_2Cl_2 (40 mL). The resulting solution was cooled in an ice bath and a solution of *N*-bromosuccinimide (6.269 g, 35.22 mmol) in CH_2Cl_2 (40 ml) was added. The reaction mixture was stirred for 12 h at room temperature. Then, the solvent was removed and the product was purified with a silica gel flash column eluted with 100% EtOAc to afford compound **3b** (4.381 g, 68%) as a yellow oil.

^{1}H NMR (400 MHz, $CDCl_3$): δ_H = 7.66 (dd, 2H, *J* = 10.5, 8.5 Hz), 7.49 (dd, 2H, *J* = 8.5, 2.0 Hz), 4.49 (s, 2H), 1.89 (m, 4H), 1.55 (m, 2H), 1.34 (m, 6H), 0.85 (t, 6H, *J* = 7.0 Hz).

^{13}C NMR (100.6 MHz, $CDCl_3$): δ_C = 141.2 (d, *J* = 3.0 Hz), 133.2 (d, *J* = 91.0 Hz), 131.1 (d, *J* = 9.0 Hz), 129.2 (d, *J* = 11.5 Hz), 32.4, 29.8 (d, *J* = 68.5 Hz), 24.2 (d, *J* = 14.5 Hz), 23.6 (d, *J* = 4.0 Hz), 13.6.

^{31}P NMR (161.3 MHz, $CDCl_3$): δ_P = 40.4.

MS (ES+): m/z (%) = 333.1 $[M+H]^+$.

HRMS (ES+): calcd for $C_{15}H_{25}OBrP$ 331.0826 $[M+H]^+$, found 331.0834 $[M+H]^+$.

FT-IR (ATR): 2957, 2929, 2868, 1465, 1402, 1164, 1111, 774 ν_{max}/cm^{-1}.

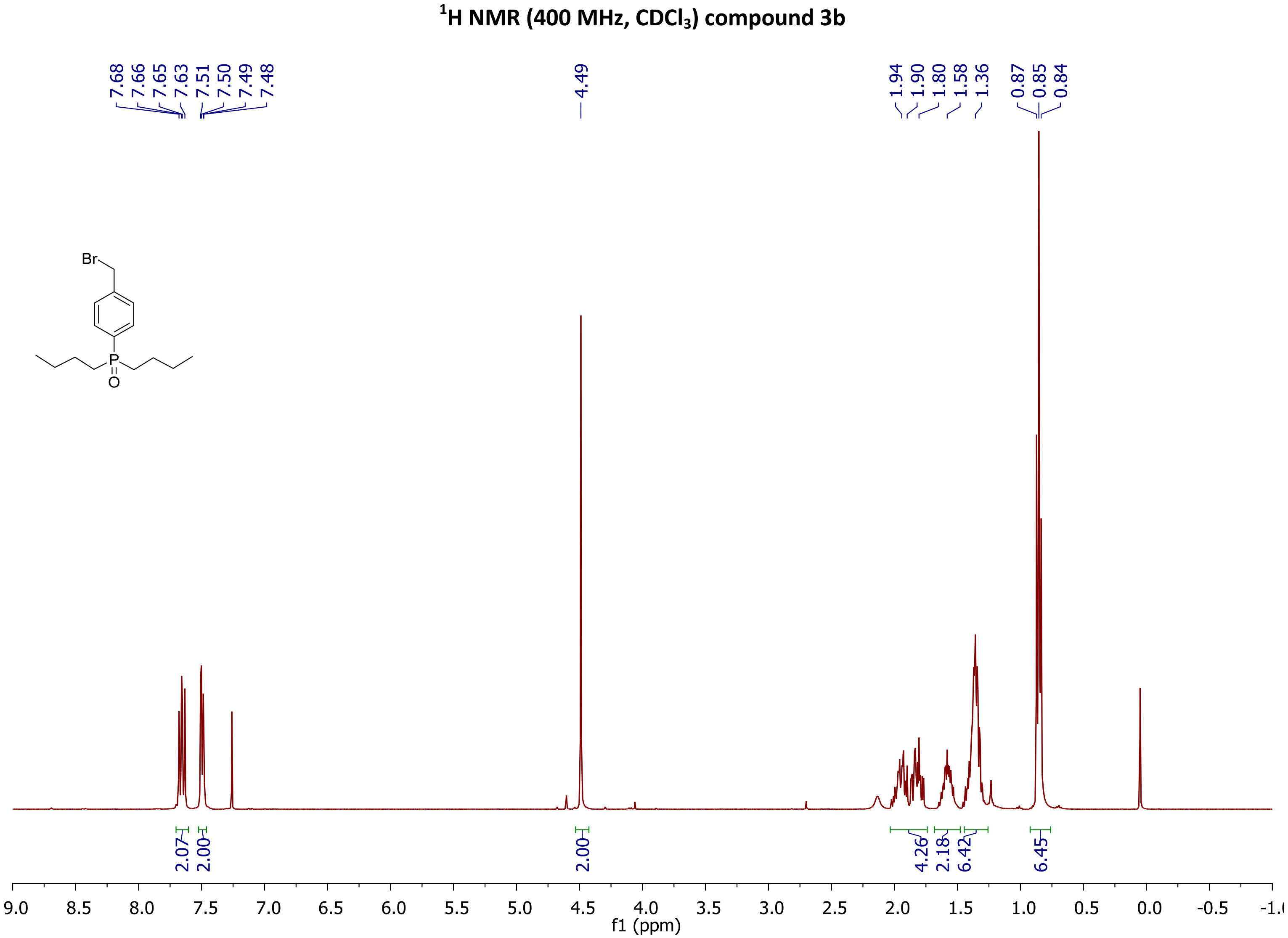

^{1}H NMR (400 MHz, CDCl$_3$) compound 3b
7.68
7.66
7.65
7.63
7.51
7.50
7.49
7.48
4.49
1.94
1.90
1.80
1.58
1.36
0.87
0.85
0.84
Br
P
O
2.07
2.00
2.00
4.26
2.18
6.42
6.45
9.0 8.5 8.0 7.5 7.0 6.5 6.0 5.5 5.0 4.5 4.0 3.5 3.0 2.5 2.0 1.5 1.0 0.5 0.0 -0.5
f1 (ppm)

^{13}C NMR (100.6 MHz, $CDCl_3$) compound 3b

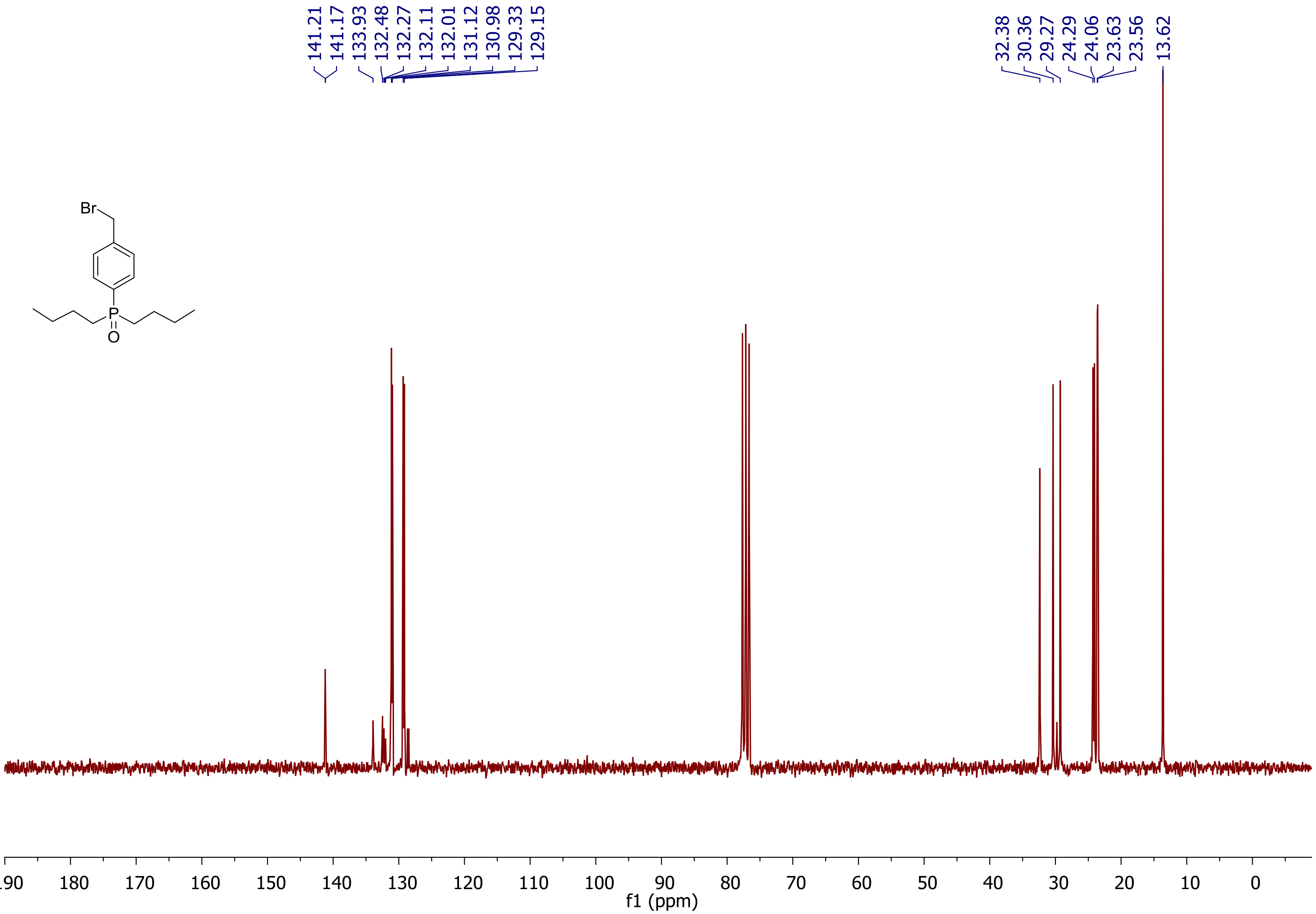

^{31}P NMR (161.3 MHz, $CDCl_3$) compound 3b

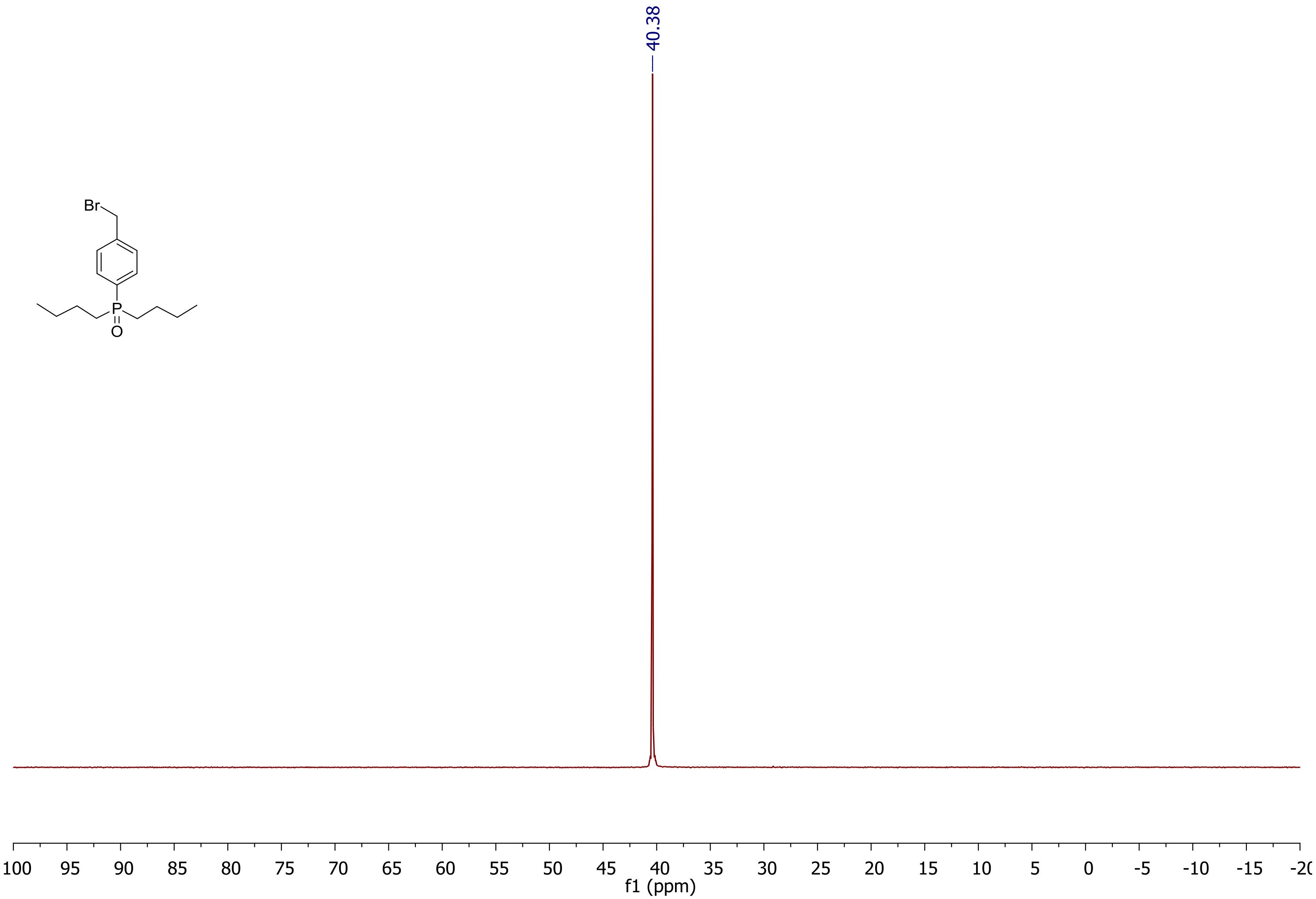

Synthesis of 7.

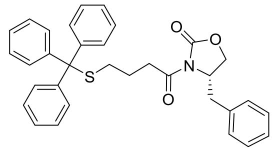

Compound **7** was synthesized in two steps. Firstly, 4-(tritylthio)butanoic acid was prepared from commercial 4-bromobutyric acid (**6**) following a described procedure (yield: 85 %).[S2] A solution of 4-(tritylthio)butanoic acid (1.860 g, 5.13 mmol) in dry THF (40 mL) were added Et_3N (2.145 mL, 15.39 mmol) and Piv-Cl (0.695 mL, 5.64 mmol) at 0 ^oC. The mixture was stirred at room temperature for 2 h. To the solution were added LiCl (1.087 g, 25.65 mmol) and (*S*)-4-Benzyl-2-oxazolidinone (1.000 g, 5.64 mmol), and the mixture was stirred at room temperature for 15 h. The reaction was quenched by the addition of saturated aqueous NH_4Cl solution (10 mL) at 0 ^oC, giving a slurry that was extracted with EtOAc (3x30 mL). The combined organic layers were washed with brine, dried over anhydrous $MgSO_4$, and concentrated *in vacuo*. The residue was purified by silica gel flash chromatography (EtOAc:hexane, 1:5) to give compound **7** (2.545 g, 95%; 13% overall yield over 2 steps) as a white foam.

$[\alpha]_D^{20}$: +28.0 (*c* 1.36, $CHCl_3$).

^{1}H NMR (400 MHz, $CDCl_3$): δ_H = 7.45-7.15 (m, 20H), 4.61 (m, 1H), 4.15 (m, 2H), 3.24 (dd, 1H, *J =* 13.5 Hz, 3.5 Hz), 2.88 (m, 2H), 2.70 (dd, 1H, *J =* 13.5 Hz, 9.5 Hz), 2.25 (m, 2H), 1.77 (m, 2H).

^{13}C NMR (100.6 MHz, $CDCl_3$): δ_C = 172.5, 153.5, 145.0, 135.4, 130.3, 129.7, 129.5, 129.1, 128.0, 127.5, 126.8, 66.8, 66.3, 55.2, 38.0, 34.9, 31.4, 23.3.

MS (ES+): m/z (%) = 544.3 $[M+Na]^+$.

HRMS (ES+): calcd for $C_{33}H_{31}NO_3NaS$ 544.1922 $[M+Na]^+$, found 544.1934 $[M+Na]^+$.

FT-IR (ATR): 1780, 1697, 1385, 1219, 771 ν_{max}/cm^{-1}.

(S2) Qvit, N., Reuveni, H., Gazal, S., Zundelevich, A., Blum, G., Niv, M. Y., Feldstein, A., Meushar, S., Shalev, D. E., Friedler, A., and Gilon, C. *J. Comb. Chem.* **2008**, *10*, 256-266.

^{1}H NMR (400 MHz, $CDCl_3$) compound 7.

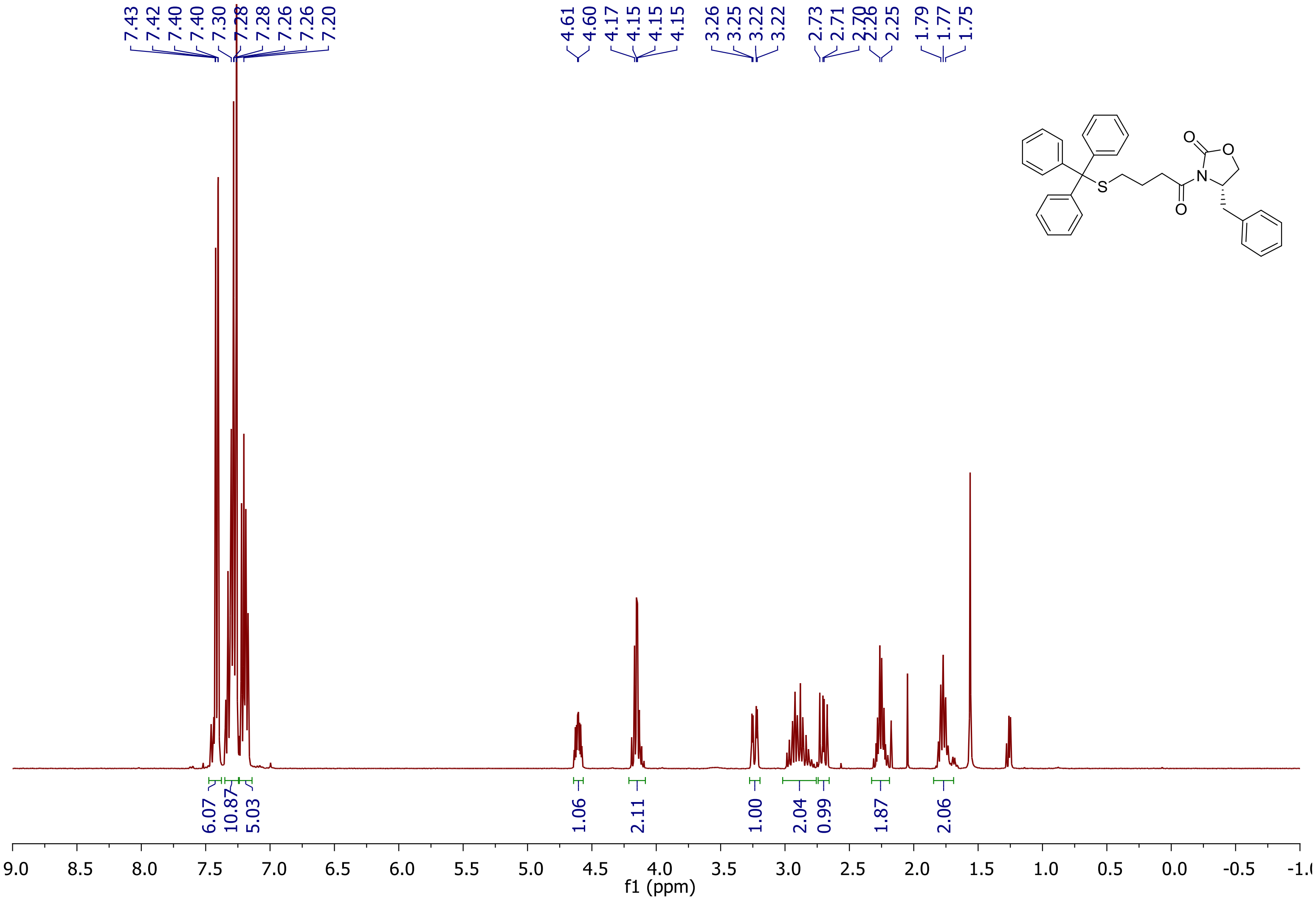

^{13}C NMR (100.6 MHz, $CDCl_3$) compound 7.

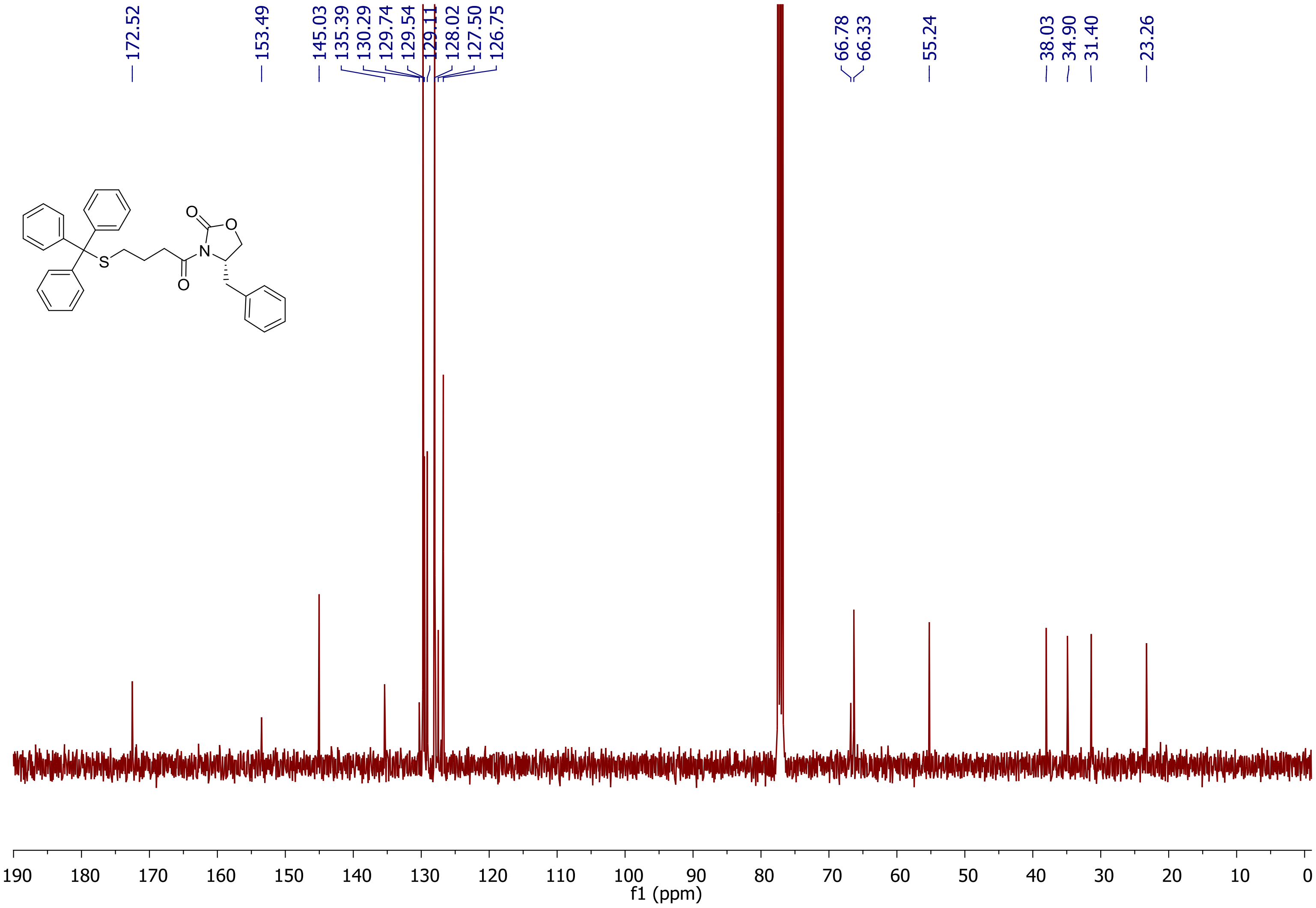

General procedure for the synthesis of compounds 8a and 8b.

To a 1 M solution of LiHMDS in dry THF (32.39 mL, 32.39 mmol), cooled to -75 °C, was added dropwise a solution of **7** (9.92 g, 19.05 mmol) in dry THF (200 mL). After stirring for 1 h, a solution of **3a** or **3b** (32.39 mmol) in dry THF (100 mL) was added dropwise at -75 °C. The reaction temperature was gradually raised from -75 to 0 °C over 1 h, and stirring was continued for 1.5 h at 0 °C. Then, saturated aqueous NH_4Cl solution was added (100 mL) with cooling and the mixture was partitioned between EtOAc (50 mL) and H_2O (50 mL), and the aqueous layer was extracted with EtOAc (3x100 mL). The combined organics were washed with brine and dried ($MgSO_4$), and the residue was purified by flash chromatography.

Compound 8a.

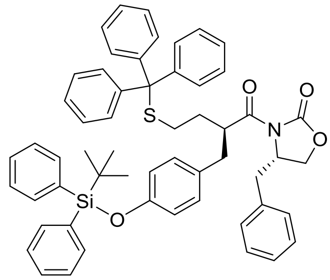

Compound **8a** was obtained following the general procedure above as a syrup (yield: 86%) using EtOAc/hexane 1:9 as the solvent system for flash chromatography.

$[\alpha]_D^{20}$: +10.4 (*c* 0.96, $CHCl_3$).

^{1}H NMR (400 MHz, $CDCl_3$): δ_H = 7.66 (m, 4H), 7.45-7.15 (m, 24H), 7.00 (m, 2H), 6.89 (d, 2H, *J* = 8.5 Hz), 6.63 (d, 2H, *J* = 8.5 Hz), 4.47 (m, 1H), 4.00 (m, 3H), 3.24 (dd, 1H, *J* = 13.5 Hz, 3.0 Hz), 2.78 (dd, 1H, *J* = 13.5 Hz, 7.5 Hz), 2.45 (dd, 1H, *J* = 13.5 Hz, 7.5 Hz), 2.35 (dd, 1H, *J* = 13.5 Hz, 9.5 Hz), 2.09 (m, 2H), 1.78 (m, 1H), 1.49 (m, 1H), 1.07 (s, 9H).

^{13}C NMR (100.6 MHz, $CDCl_3$): δ_C = 175.3, 154.3, 152.9, 145.0, 135.6, 135.4, 133.2, 131.0, 130.2, 130.0, 129.9, 129.7, 129.5, 129.0, 128.0, 127.9, 127.8, 127.4, 126.7, 119.6, 66.8, 65.9, 55.3, 44.3, 37.8, 37.7, 30.7, 29.9, 26.7, 19.6.

MS (ES+): m/z (%) = 888.6 $[M+Na]^+$.

HRMS (ES+): calcd for $C_{56}H_{55}NO_4NaSSi$ 888.3519 $[M+Na]^+$, found 888.3489 $[M+Na]^+$.

FT-IR (ATR): 2930, 2857, 1778, 1695, 1509, 1386, 1252, 1107, 918, 822 ν_{max}/cm^{-1}.

^{1}H NMR (400 MHz, CDCl$_3$) compound 8a.

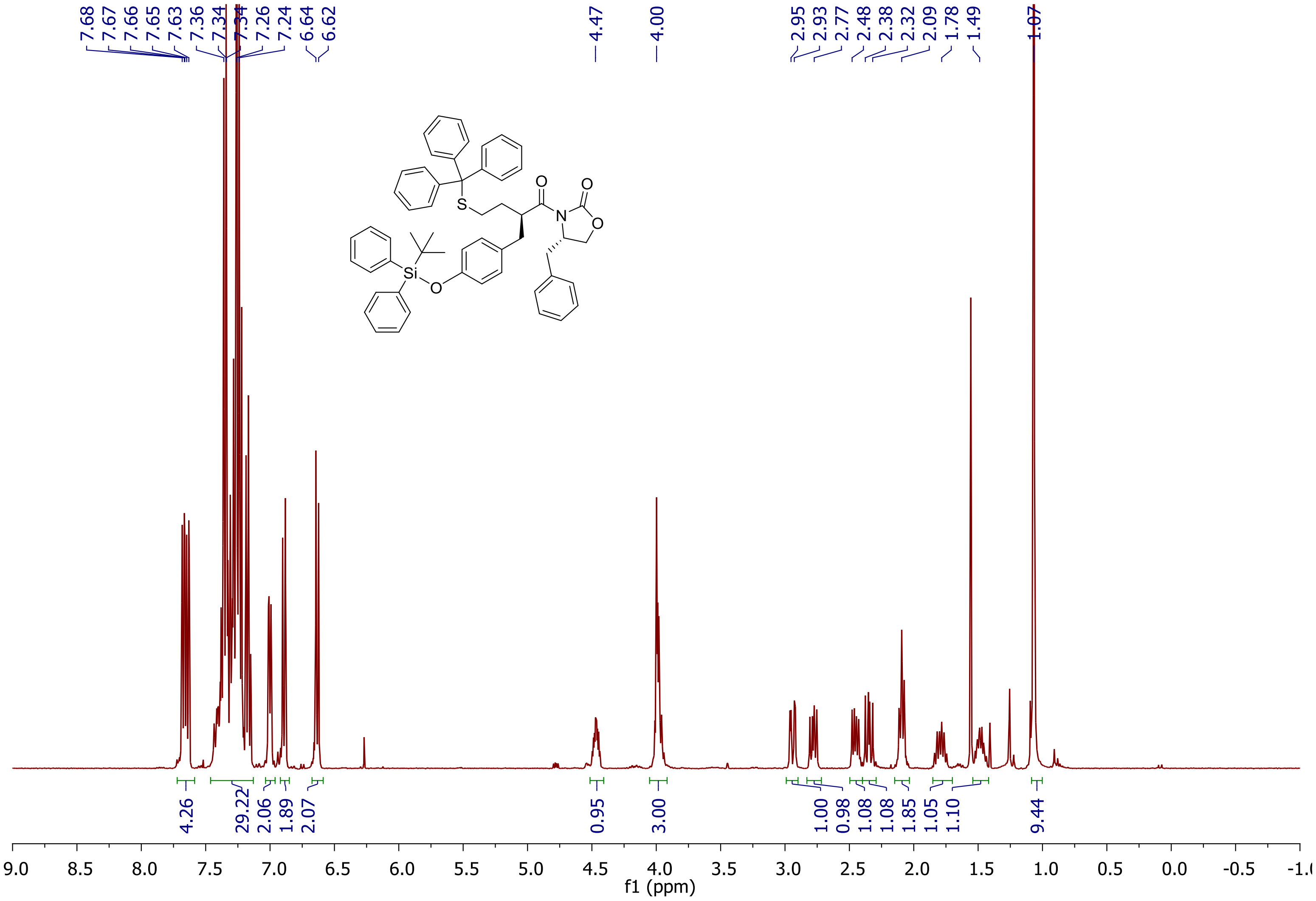

^{13}C NMR (100.6 MHz, CDCl$_3$) compound 8a.

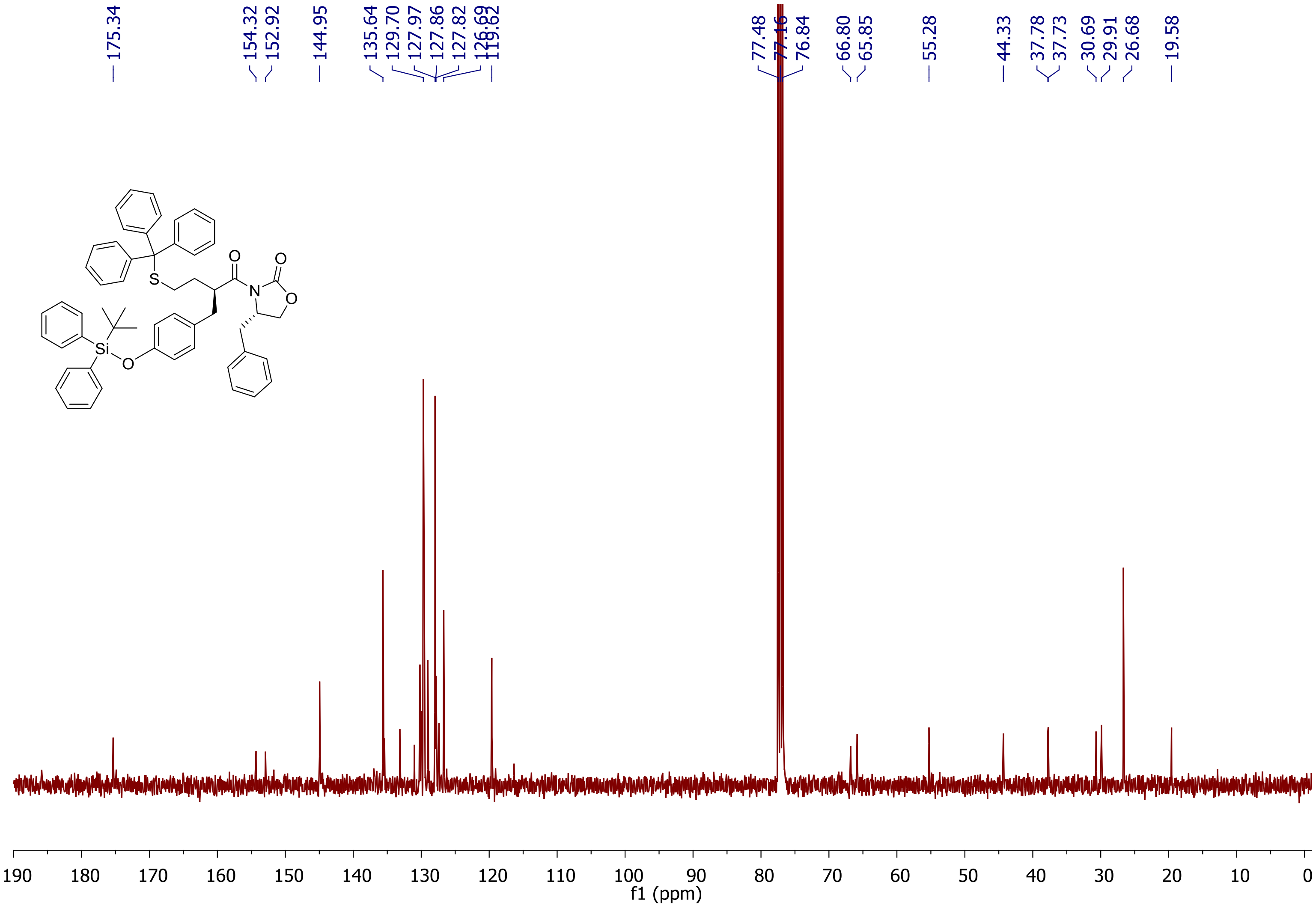

Compound 8b.

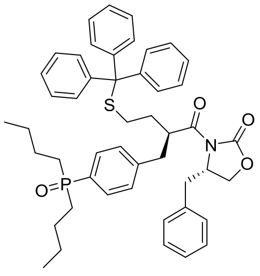

Compound **8b** was obtained following the general procedure described before as a syrup (yield: 87%) using CH_2Cl_2:MeOH 49:1 as the solvent system for flash chromatography.

$[\alpha]_D^{20}$: +20.2 (*c* 0.99, $CHCl_3$).

^{1}H NMR (400 MHz, $CDCl_3$): δ_H = 7.57 (m, 2H), 7.40-7.15 (m, 20H), 7.02 (d, 2H, *J* = 9.0 Hz), 4.49 (m, 1H), 4.05 (m, 3H), 2.97 (m, 4H), 2.61 (dd, 1H, *J* = 13.5, 7.0 Hz), 2.41 (dd, 1H, *J* = 13.5, 9.5 Hz), 2.15 (m, 2H), 1.84 (m, 5H), 1.53 (m, 3H), 1.29 (m, 6H), 0.83 (t, 3H, *J* = 7.0 Hz), 0.80 (t, 3H, *J* = 7.0 Hz).

^{13}C NMR (100.6 MHz, $CDCl_3$): δ_C = 174.8, 153.0, 144.9, 142.6, 135.1, 130.7, 130.6, 129.8, 129.7, 129.4, 129.1, 128.0, 127.5, 126.8, 66.9, 66.0, 55.4, 43.9, 38.2, 37.7, 30.8, 29.8, 29.9 (d, *J* = 68.5 Hz), 29.8 (d, *J* = 68.5 Hz), 24.2 (d, *J* = 14.5 Hz), 23.6, 13.7 (d, *J* = 6.0 Hz).

^{31}P NMR (161.3 MHz, $CDCl_3$): δ_P = 40.43.

MS (ES+): m/z (%) = 772.4 $[M+H]^+$.

HRMS (ES+): calcd for $C_{48}H_{55}NO_4SP$ 772.3589 $[M+H]^+$, found 772.3593 $[M+H]^+$.

FT-IR (ATR): 2972, 2930, 1777, 1694, 1387, 1164, 1109, 744 ν_{max}/cm^{-1}.

^{1}H NMR (400 MHz, $CDCl_3$) compound 8b.

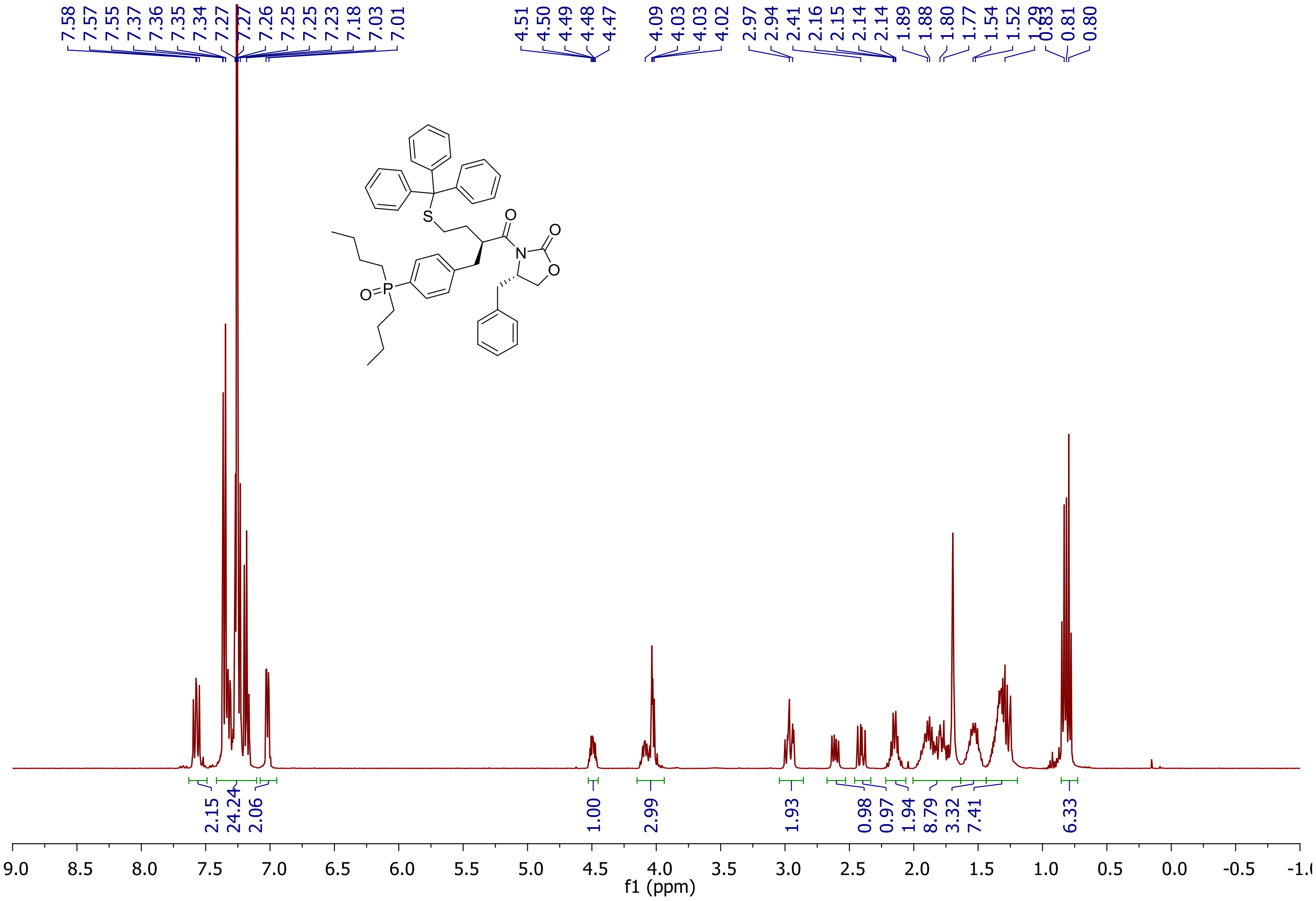

^{13}C NMR (100.6 MHz, CDCl$_3$) compound 8b.

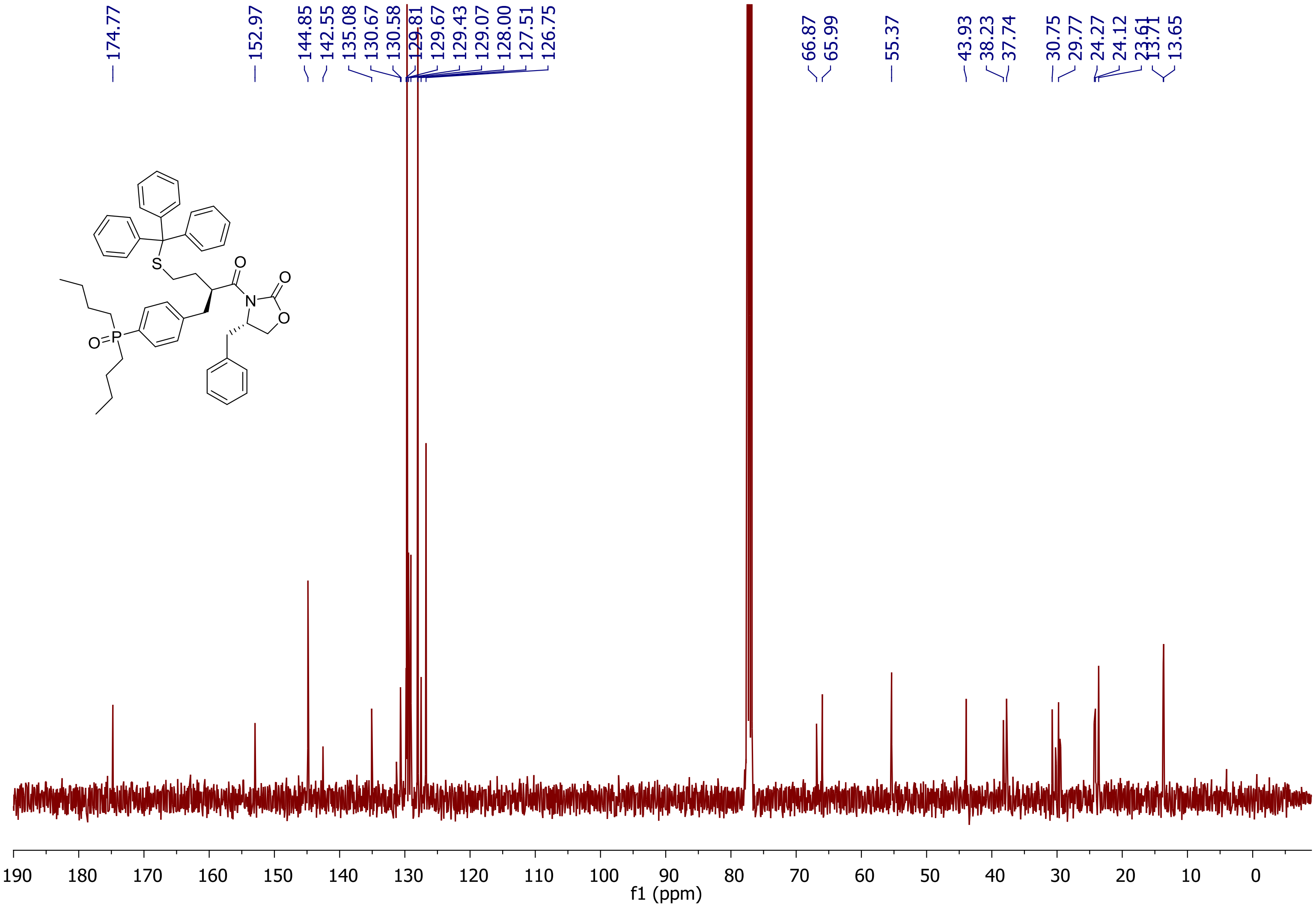

^{31}P NMR (161.3 MHz, $CDCl_3$) compound 8b.

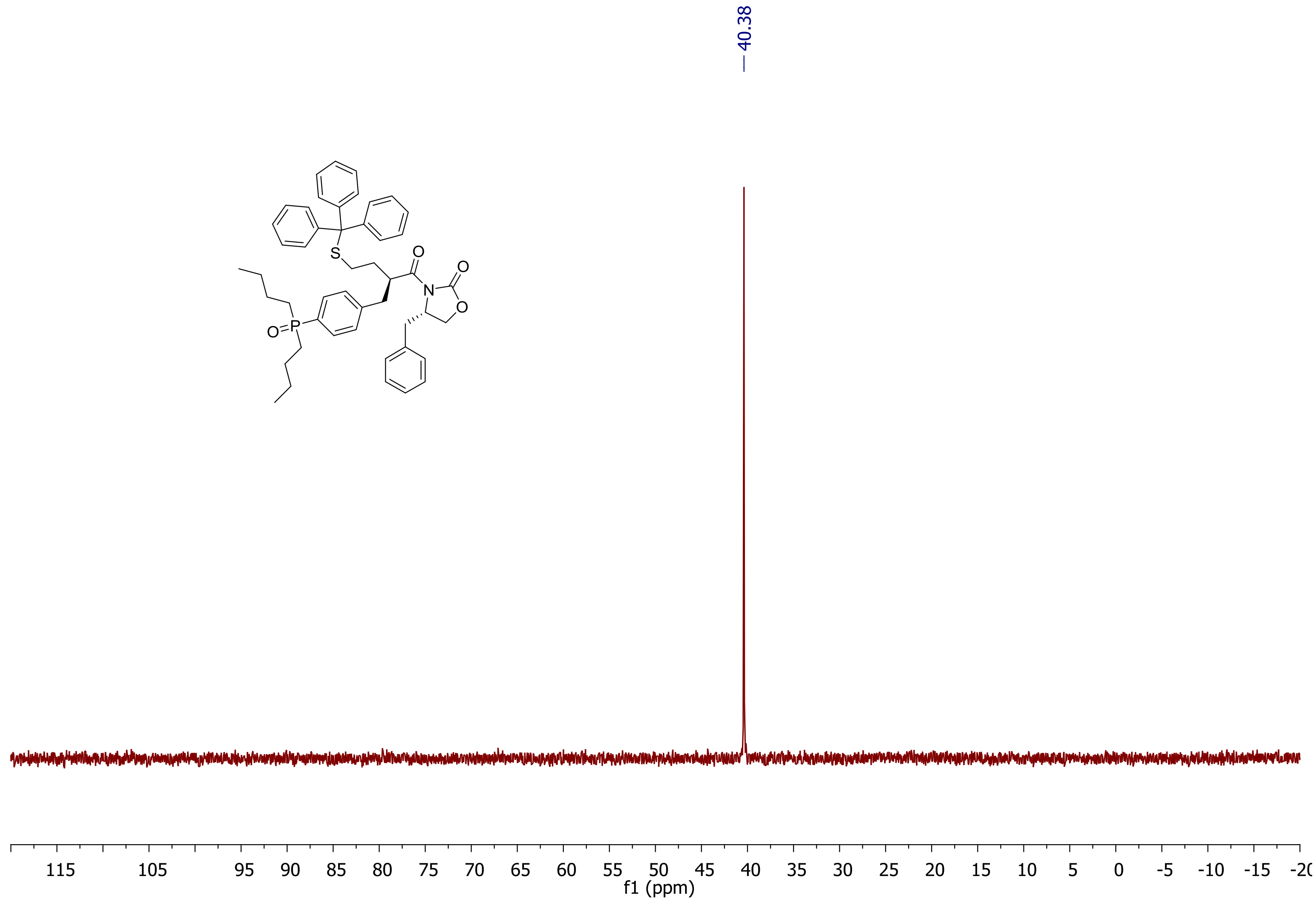

General procedure for the synthesis of compounds 9a and 9b.

To a solution of alcohol **8a** or **8b** (16.29 mmol) in THF (100 mL) at 0 °C was added a solution of $NaBH_4$ (3.084 g, 81.44 mmol) in H_2O (30 mL). The mixture was stirred at 0 °C for 10 min, warmed to room temperature over 30 min, then stirred for 5 h. The reaction was quenched with saturated aqueous NH_4Cl solution (30 mL) and stirred for additional 1 h. The layers were separated, and the aqueous phase was extracted with EtOAc (3x50 mL). The organic extracts were combined, washed with saturated aqueous $NaHCO_3$ solution (1x25 mL) and brine (1x25 mL), dried over $MgSO_4$ then concentrated *in vacuo*. The crude product was purified by flash chromatography.

Compound 9a.

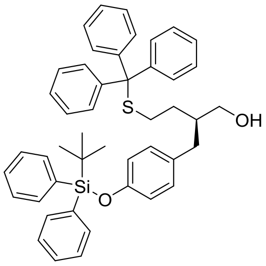

Compound **9a** was obtained from compound **8a** following the general procedure above as a colourless oil (yield: 86%) using EtOAc/hexane 1:5 as the solvent system for flash chromatography.

$[\alpha]_D^{20}$: +8.5 (*c* 0.82, $CHCl_3$).

^{1}H NMR (400 MHz, $CDCl_3$): δ = 7.70 (m, 4H), 7.40-7.20 (m, 21H), 6.78 (d, 2H, *J* = 8.5 Hz), 6.64 (d, 2H, *J* = 8.5 Hz), 3.27 (m, 2H), 2.32 (m, 2-H), 2.16 (m, 2H), 1.63 (m, 1H), 1.37 (m, 2H), 1.26 (bs, 1H), 1.10 (s, 9H).

^{13}C NMR (100.6 MHz, $CDCl_3$): δ_C = 153.9, 145.1, 135.7, 133.2, 132.5, 130.3, 130.0, 129.9, 129.7, 128.0, 127.9, 126.7, 119.7, 66.8, 64.4, 41.9, 36.6, 29.9, 29.7, 26.7, 19.6.

MS (ES+): m/z (%) = 715.5 $[M+Na]^+$.

HRMS (ES+): calcd for $C_{46}H_{48}O_2NaSiS$ 715.3042 $[M+Na]^+$, found 715.3033 $[M+Na]^+$.

FT-IR (ATR): 2928, 2857, 1607, 1508, 1428, 1254, 1113, 919 ν_{max}/cm^{-1}.

^{1}H NMR (400 MHz, $CDCl_3$) compound 9a.

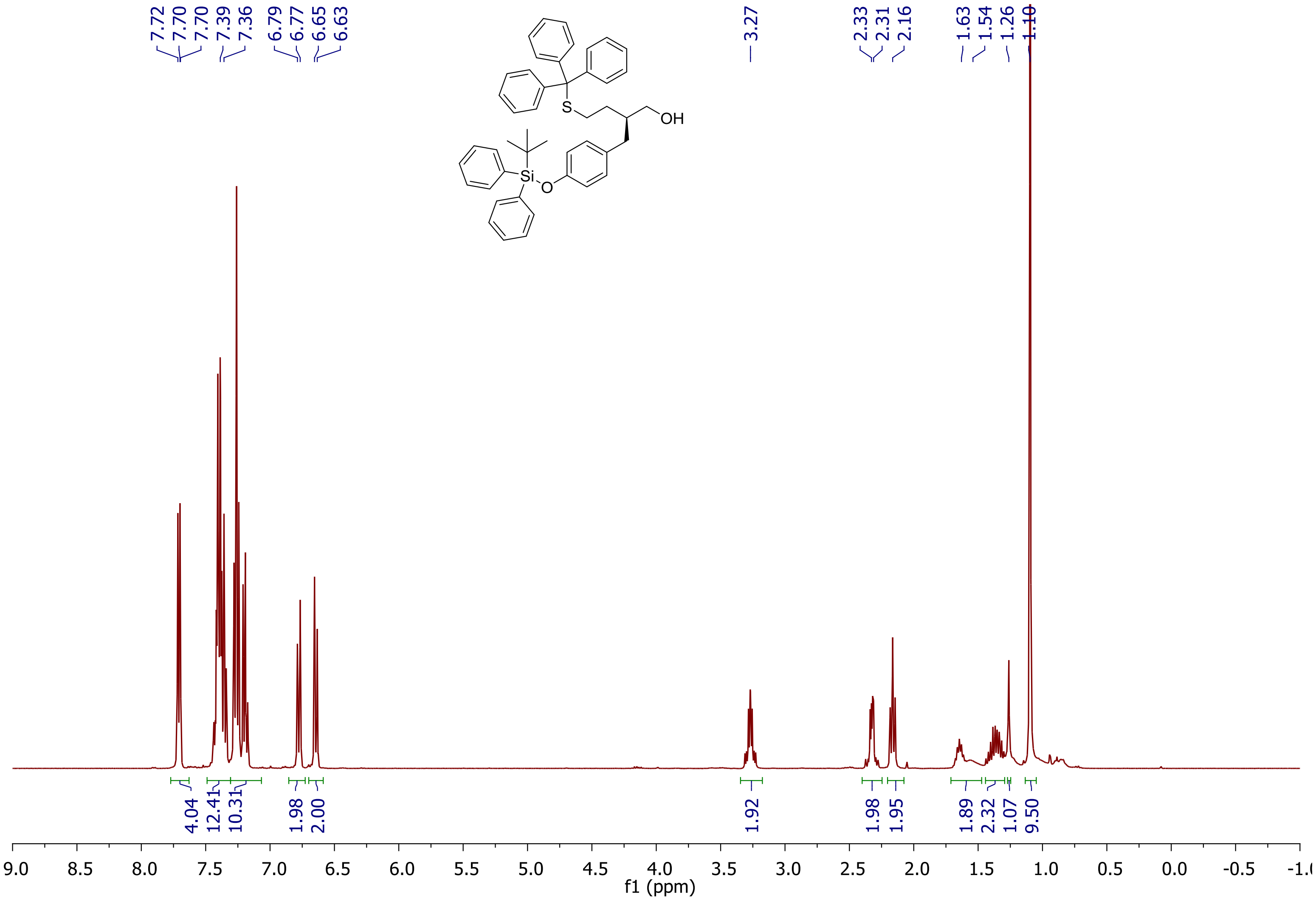

^{13}C NMR (100.6 MHz, $CDCl_3$) 9a.

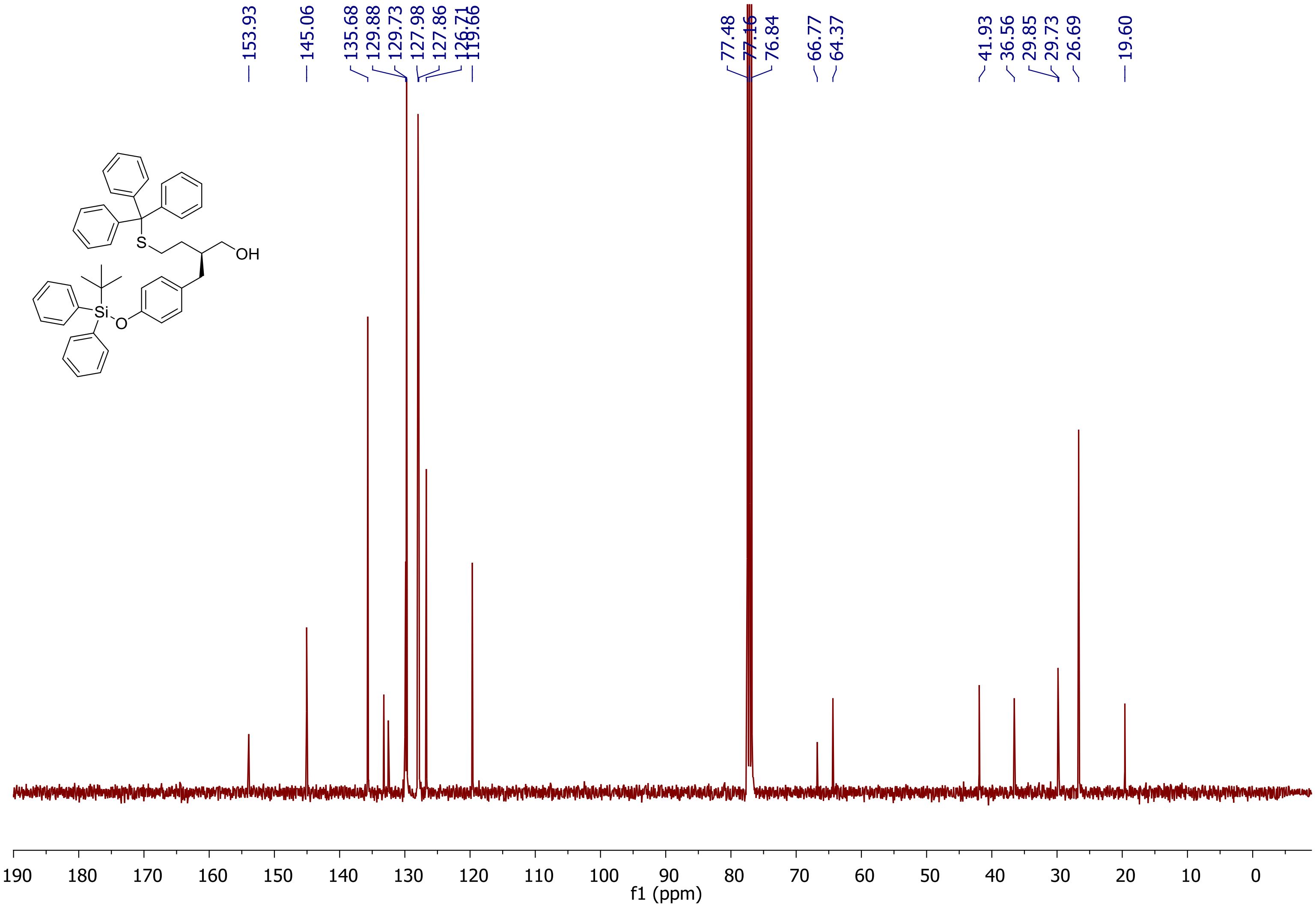

Compound 9b.

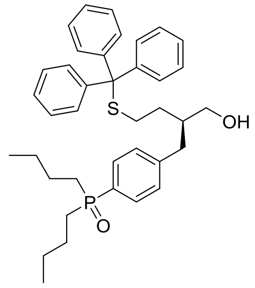

Compound **9b** was obtained from compound **8b** following the general procedure described before as a colourless oil (yield: 88%) using CH_2Cl_2:MeOH 35:1 as the solvent system for flash chromatography.

$[\alpha]_D^{20}$: +6.9 (*c* 1.44, $CHCl_3$).

^{1}H NMR (400 MHz, $CDCl_3$): δ_H = 7.55 (dd, 2H, *J* = 10.5, 8.0 Hz), 7.40-7.15 (m, 17H), 3.31 (m, 2H), 2.52 (m, 2-H), 2.22 (t, 2H, *J* = 7.5 Hz), 1.88 (m, 5H), 1.59 (m, 3H), 1.38 (m, 8H), 0.86 (t, 6H, *J* = 7.0 Hz).

^{13}C NMR (100.6 MHz, $CDCl_3$): δ_C = 145.0, 144.3, 130.6, 130.5, 129.7, 129.6, 128.0, 126.8, 66.8, 63.7, 41.6, 37.2, 29.9 (d, *J* = 68.5 Hz), 29.8 (d, *J* = 68.5 Hz), 29.9, 29.7, 24.3 (d, *J* = 14.5 Hz), 23.7 (d, *J* = 4.0 Hz), 13.7.

^{31}P NMR (161.3 MHz, $CDCl_3$): δ_P = 40.7.

MS (ES+): m/z (%) = 599.3 $[M+H]^+$.

HRMS (ES+): calcd for $C_{38}H_{47}O_2PS$ 599.3113 $[M+H]^+$, found 599.3118 $[M+H]^+$.

FT-IR (ATR): 2956, 2931, 2870, 1489, 1444, 1159, 743 ν_{max}/cm^{-1}.

^{1}H NMR (400 MHz, $CDCl_3$) compound 9b.

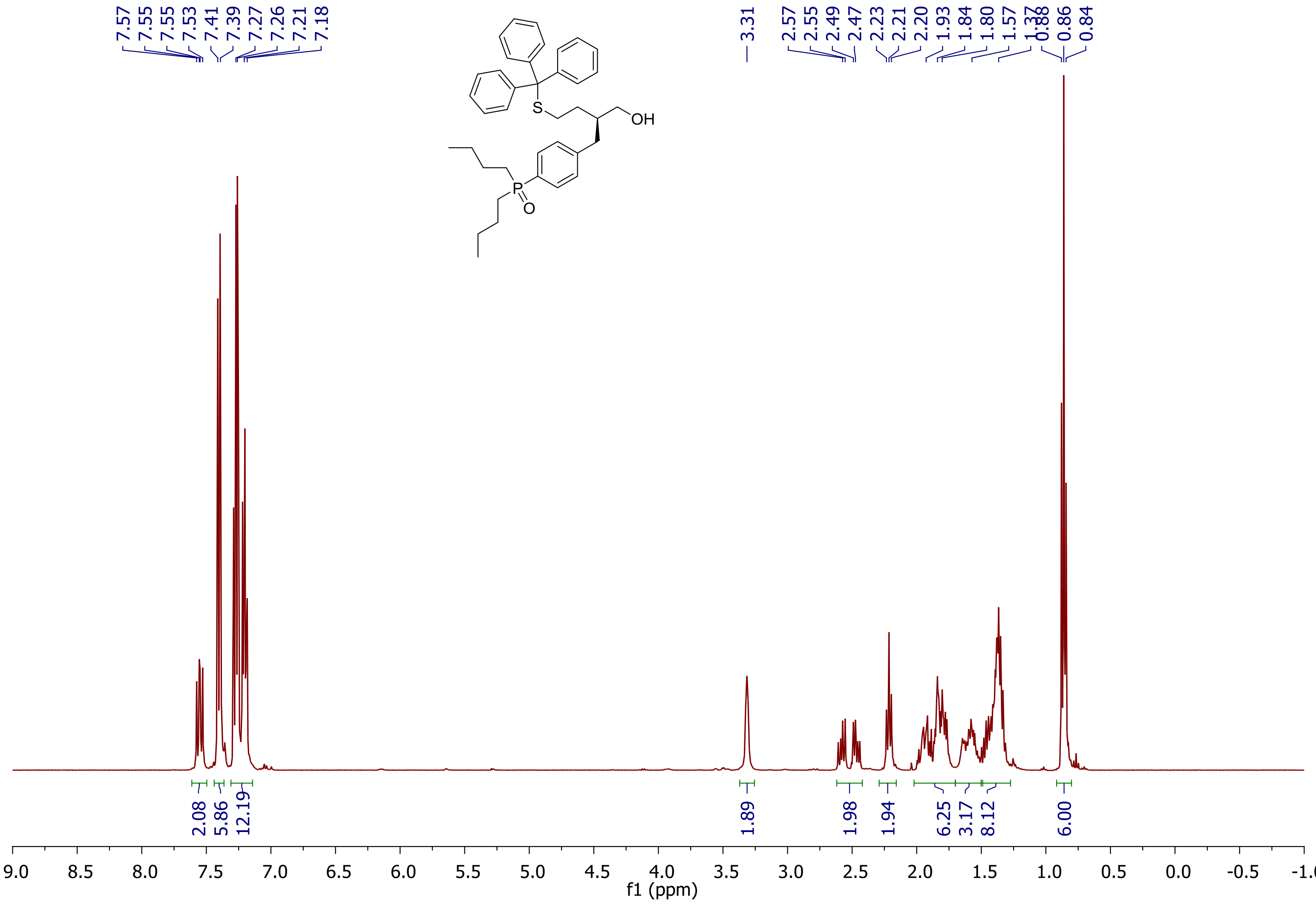

^{13}C NMR (100.6 MHz, $CDCl_3$) compound 9b.

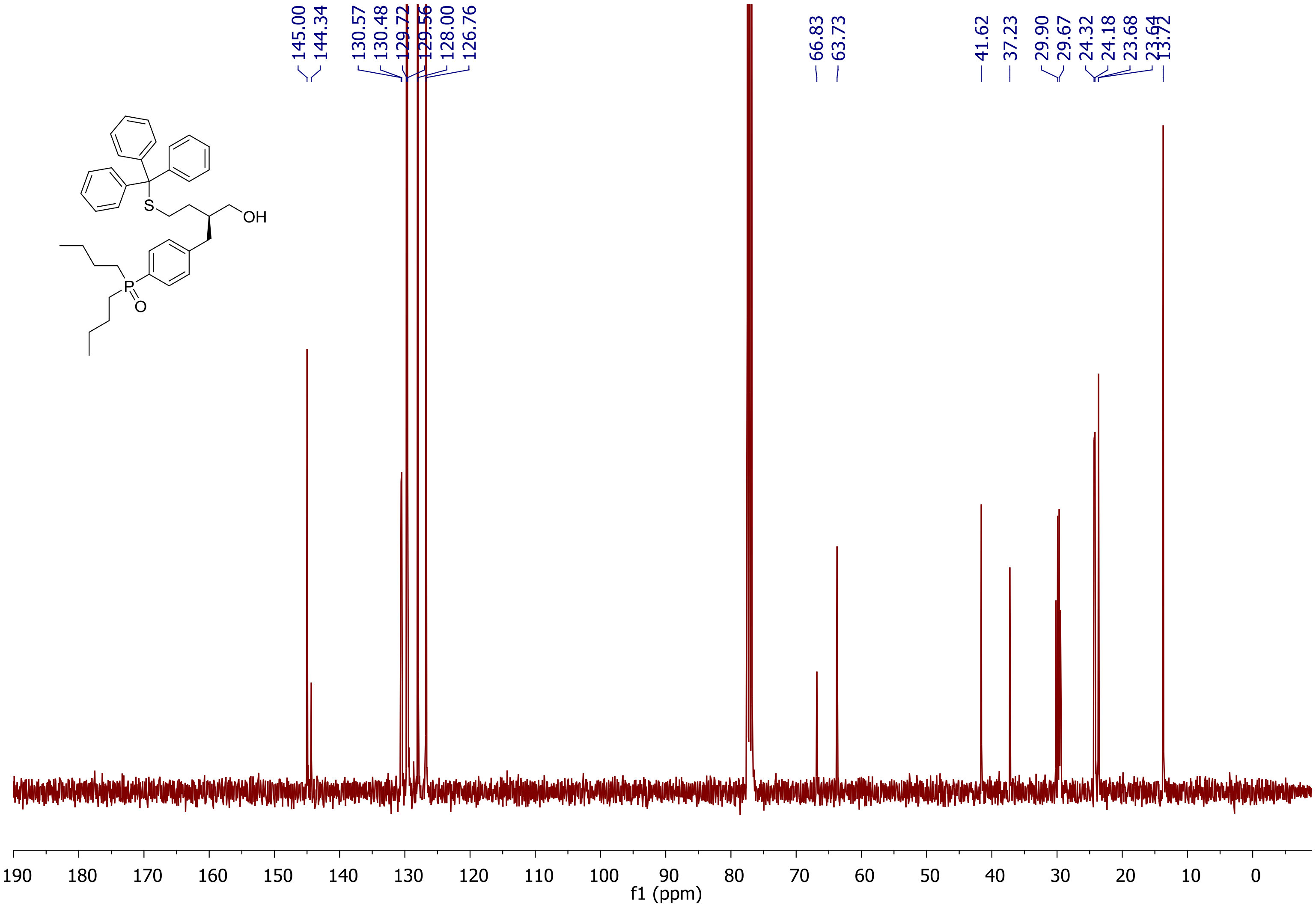

^{31}P NMR (161.3 MHz, $CDCl_3$) compound 9b.

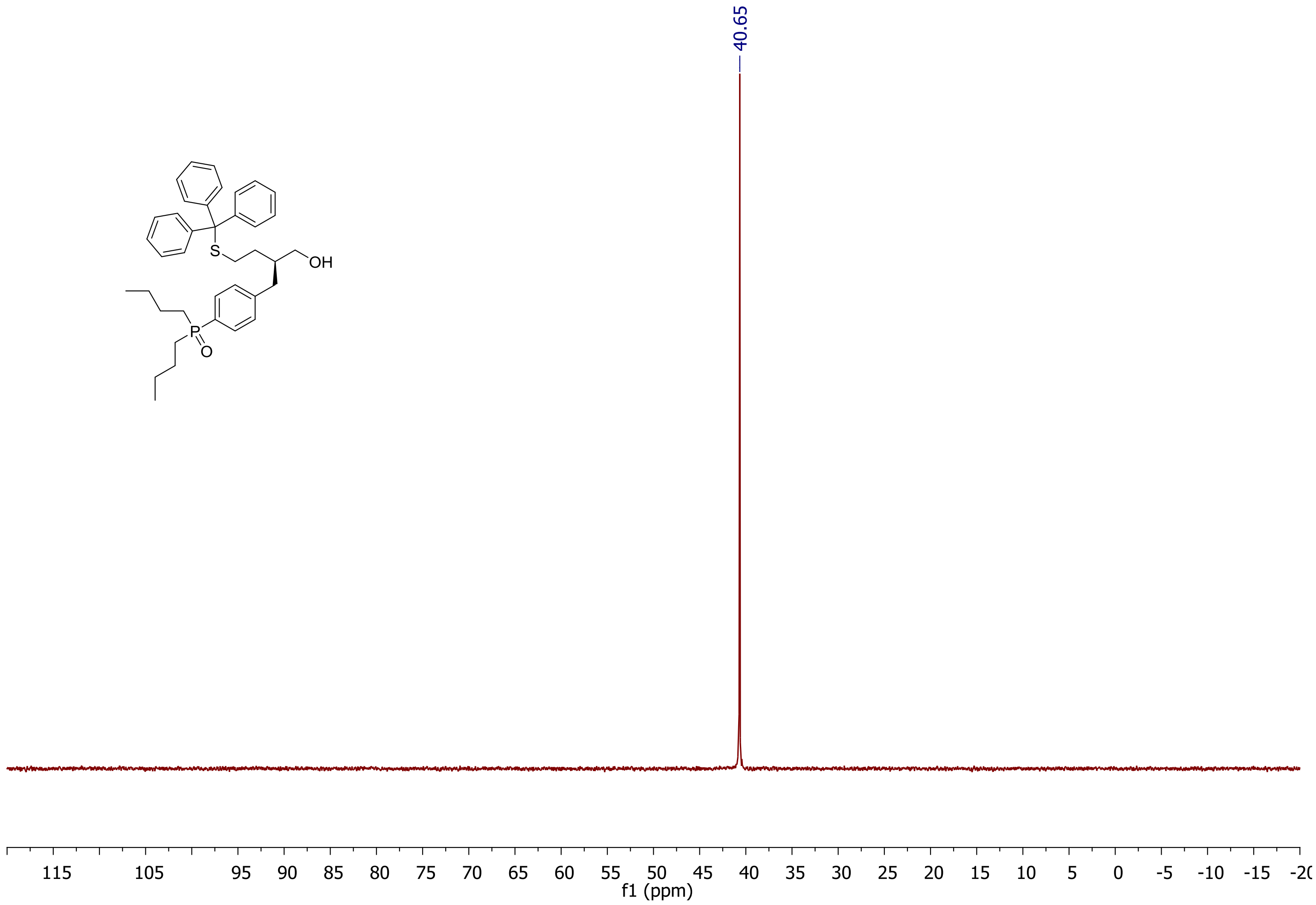

General procedure for the synthesis of 10a and 10b.

To a stirred solution of alcohol **9a** or **9b** (14.04 mmol) in dry CH_2Cl_2 (75 mL) at 0 ^oC were added Na_2CO_3 (2.232 g, 21.06 mmol) and solid Dess-Martin periodinane (6.550 g, 15.44 mmol). To the resulting clear solution, 270 ml of wet CH_2Cl_2 (prepared by shaking of 0.27 mL of H_2O with 270 ml of CH_2Cl_2) was added dropwise during a period of 20 min. The resulting opaque white reaction mixture was stirred for additional 1 h, and then a second addition of Na_2CO_3 (2.232 g, 21.06 mmol) and solid Dess-Martin periodinane (6.550 g, 15.44 mmol) were performed. After stirring for an additional 1 h, the mixture was mixed with 20 ml of diethyl ether and washed with a 1:1 mixture (50 mL) of saturated aqueous $NaHCO_3$ solution and 10% aqueous $Na_2S_2O_3$ solution. Phases were separated, and the organic phase was washed consecutively with H_2O (2x50 mL) and brine (1x50 mL). Combined organic phase was dried over anhydrous $MgSO_4$, and solvent was removed on a rotary evaporator. The obtained aldehyde was used in the following step without further purification.

To a suspension of methyl triphenylphosphonium bromide (20.062 g, 56.16 mmol) in THF (175 mL) at 0 ^oC was added dropwise a solution of *n*-butyllithium in hexanes (21.3 mL, 2.5 M, 53.35 mmol). The resulting yellow solution of the ylide was stirred for 30 min, and treated with the previously obtained aldehyde dissolved in THF (80 mL). The reaction mixture was allowed to warm to room temperature and stirred for 2 h. Saturated aqueous NH_4Cl solution (20 mL) was added, and the aqueous phase was extracted with EtOAc (3x75 mL). The combined organic layers were washed with brine and dried over $MgSO_4$. Concentration *in vacuo* followed by flash chromatography afforded the desired products.

Compound 10a.

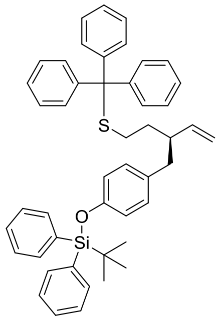

Compound **10a** was obtained from compound **9a** following the general procedure above as a yellow syrup (yield: 88%) using EtOAc:hexane 1:30 as the solvent system for flash chromatography.

$[\alpha]_D^{20}$: -4.2 (*c* 0.96, $CHCl_3$).

^{1}H NMR (400 MHz, $CDCl_3$): δ_H = 7.71 (m, 4H), 7.45-7.20 (m, 21H), 6.75 (d, 2H, *J* = 8.5 Hz), 6.64 (d, 2H, *J* = 8.5 Hz), 5.33 (ddd, 1H, *J* = 17.0, 10.5, 8.5 Hz), 4.82 (dd, 1H, *J* = 10.5, 1.5 Hz), 4.73 (dd, 1H, *J* = 17.0, 1.5 Hz), 2.35 (d, 2H, *J* = 7.0 Hz), 2.15 (m, 1H), 2.09 (m, 2H), 1.39 (m, 1H), 1.26 (m, 1H), 1.10 (s, 9H).

^{13}C NMR (100.6 MHz, $CDCl_3$): δ_C = 153.8, 145.1, 141.3, 135.7, 133.3, 132.6, 130.0, 129.9, 129.8, 127.9, 127.8, 126.6, 119.4, 115.3, 66.7, 45.1, 40.8, 32.9, 29.9, 26.7, 19.6.

MS (ES+): m/z (%) = 711.5 $[M+Na]^+$.

HRMS (ES+): calcd for $C_{47}H_{48}ONaSiS$ 711.3093 $[M+Na]^+$, found 711.3088 $[M+Na]^+$.

FT-IR (ATR): 2930, 2857, 1509, 1254, 1113, 917, 742 ν_{max}/cm^{-1}.

^{1}H NMR (400 MHz, $CDCl_3$) compound 10a.

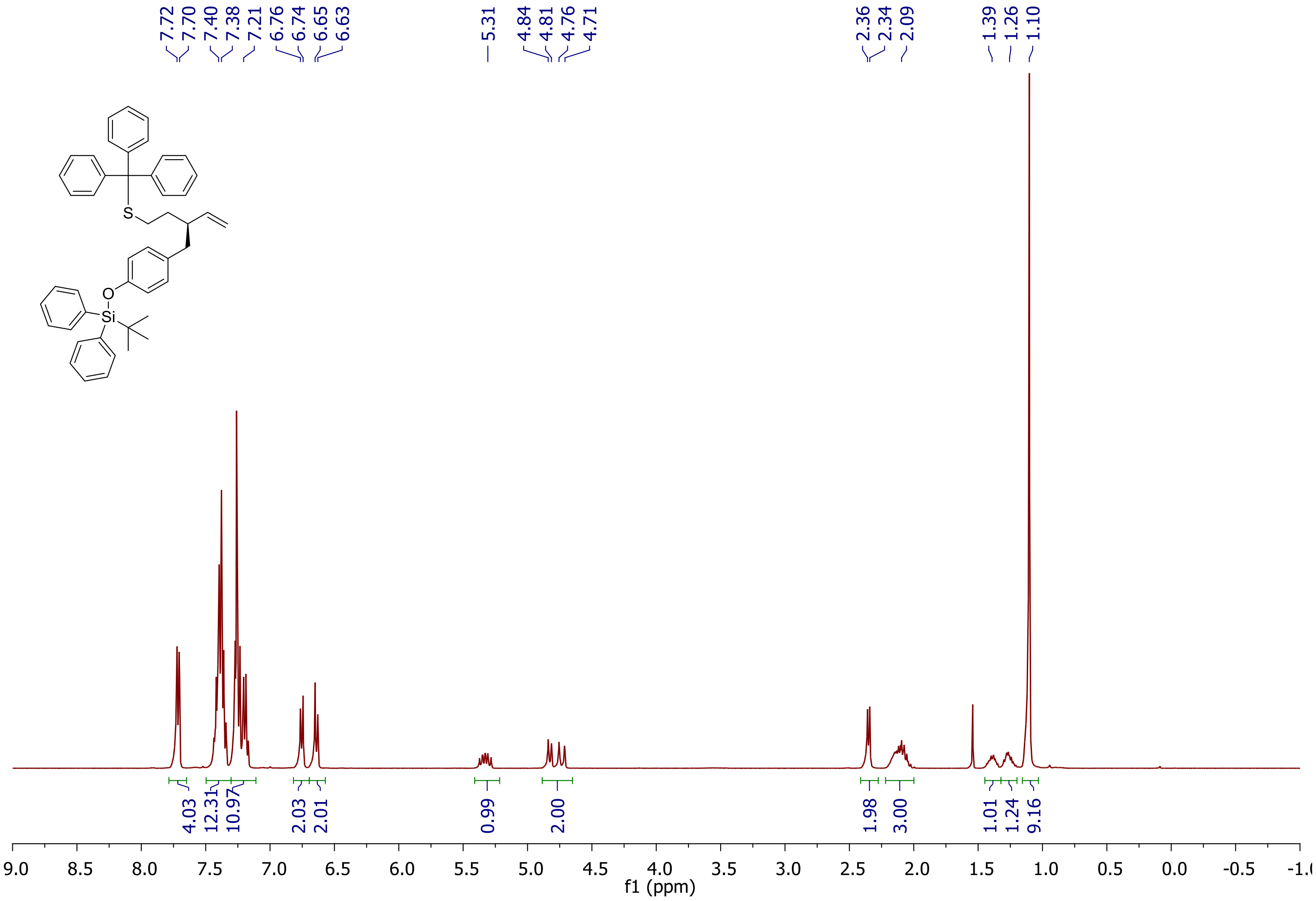

^{13}C NMR (100.6 MHz, $CDCl_3$) compound 10a.

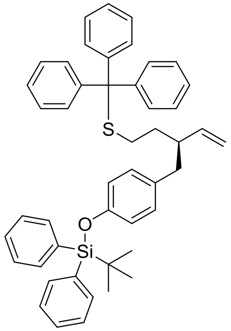

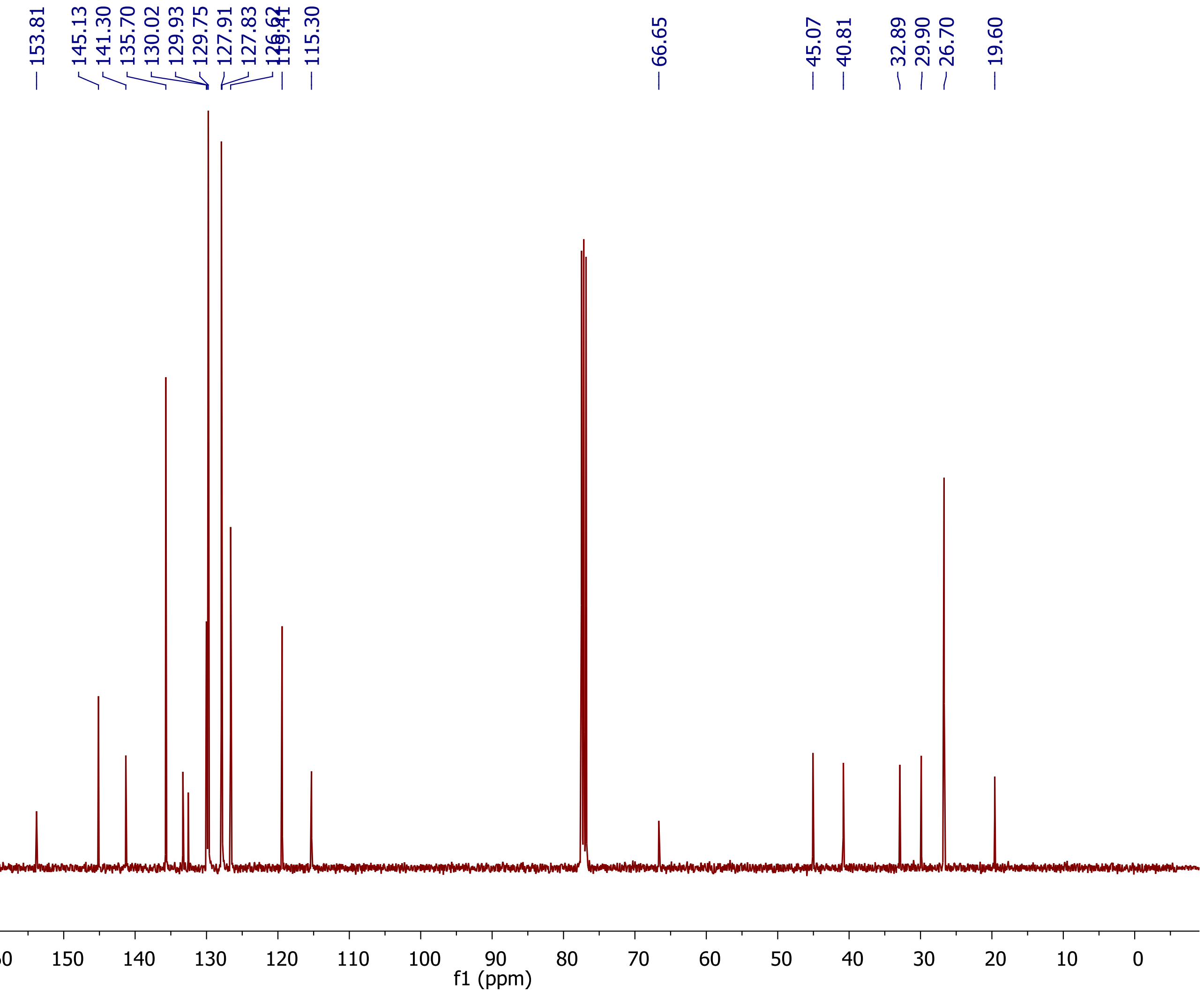

Compound 10b.

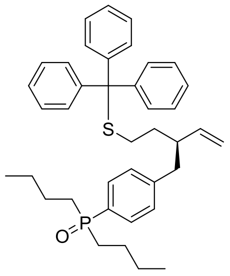

Compound **10b** was obtained from compound **9b** following the general procedure described before as a colorless oil (yield: 63%) using CH_2Cl_2:MeOH 49:1 as the solvent system for flash chromatography.

$[\alpha]_D^{20}$: -2.4 (*c* 0.85, $CHCl_3$).

^{1}H NMR (400 MHz, $CDCl_3$): δ_H = 7.55 (dd, 2H, *J* = 10.5, 8.0 Hz), 7.39 (m, 5H), 7.30-7.15 (m, 12H), 5.36 (ddd, 1H, *J* = 17.0, 10.5, 8.5 Hz), 4.86 (dd, 1H, *J* = 10.5, 1.5 Hz), 4.76 (m, 1H), 2.52 (qd, *J* = 13.5, 7.0 Hz), 2.29 (m, 1H), 2.14 (m, 2H), 1.88 (m, 4H), 1.58 (m, 2H), 1.38 (m, 8H), 0.86 (t, 6H, *J* = 7.0 Hz).

^{13}C NMR (100.6 MHz, $CDCl_3$): δ_C = 145.1, 144.0, 140.6, 130.4, 130.3, 129.8, 129.6, 127.9, 126.7, 116.0, 66.8, 44.7, 41.6, 33.3, 29.9 (d, *J* = 68.5 Hz), 29.8 (d, *J* = 68.5 Hz), 29.8, 24.2 (d, *J* = 14.5 Hz), 23.7 (d, *J* = 4.0 Hz), 13.7.

^{31}P NMR (161.3 MHz, $CDCl_3$): δ_P = 40.6.

MS (ES+): m/z (%) = 595.3 $[M+Na]^+$.

HRMS (ES+): calcd for $C_{39}H_{48}OPS$ 595.3164 $[M+H]^+$, found 595.3155 $[M+H]^+$.

FT-IR (ATR): 2956, 2929, 2869, 1600, 1488, 1443, 1167, 1111, 913 ν_{max}/cm^{-1}.

^{1}H NMR (400 MHz, $CDCl_3$) compound 10b.

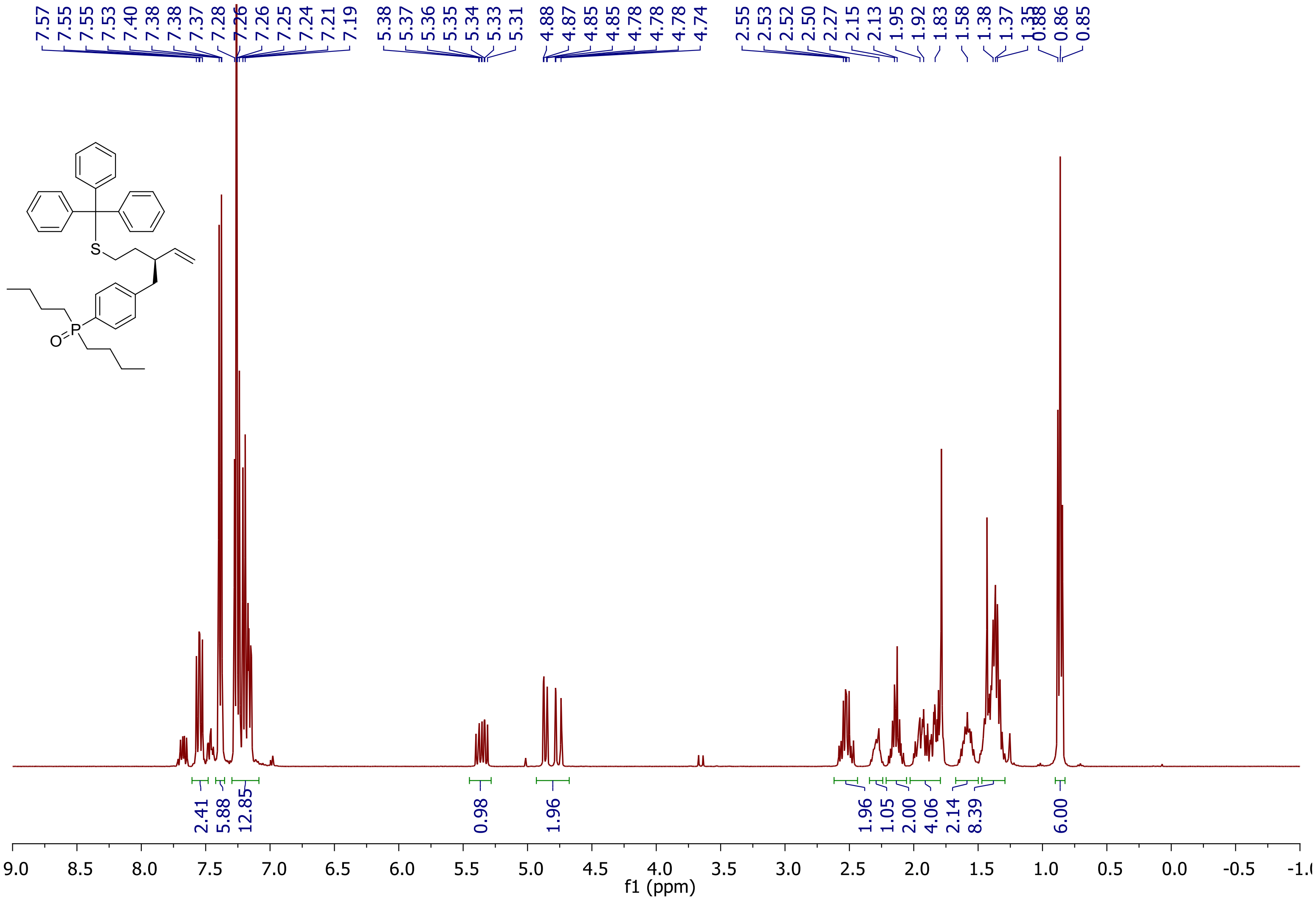

^{13}C NMR (100.6 MHz, $CDCl_3$) compound 10b.

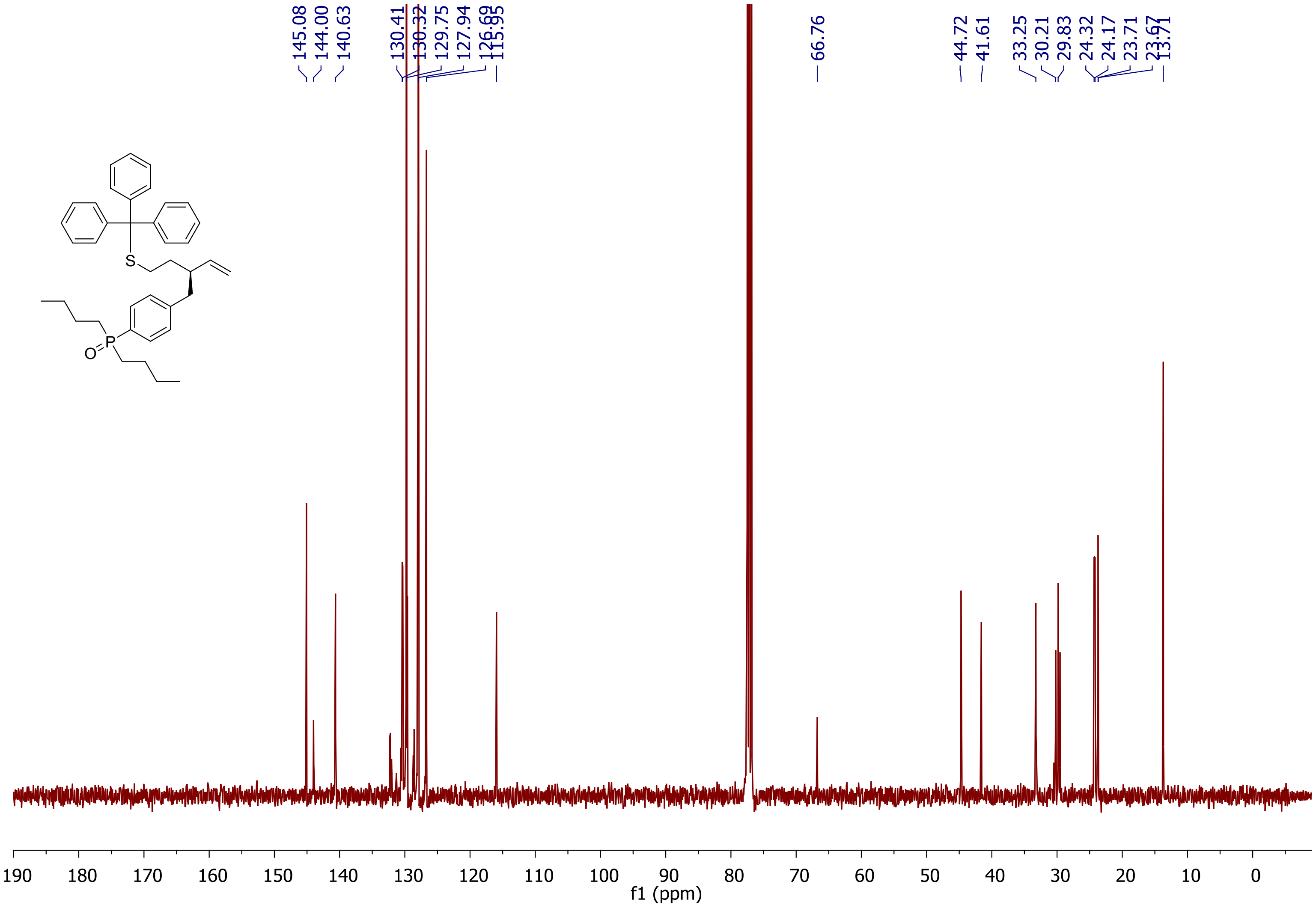

^{31}P NMR (161.3 MHz, CDCl$_3$) compound 10b.

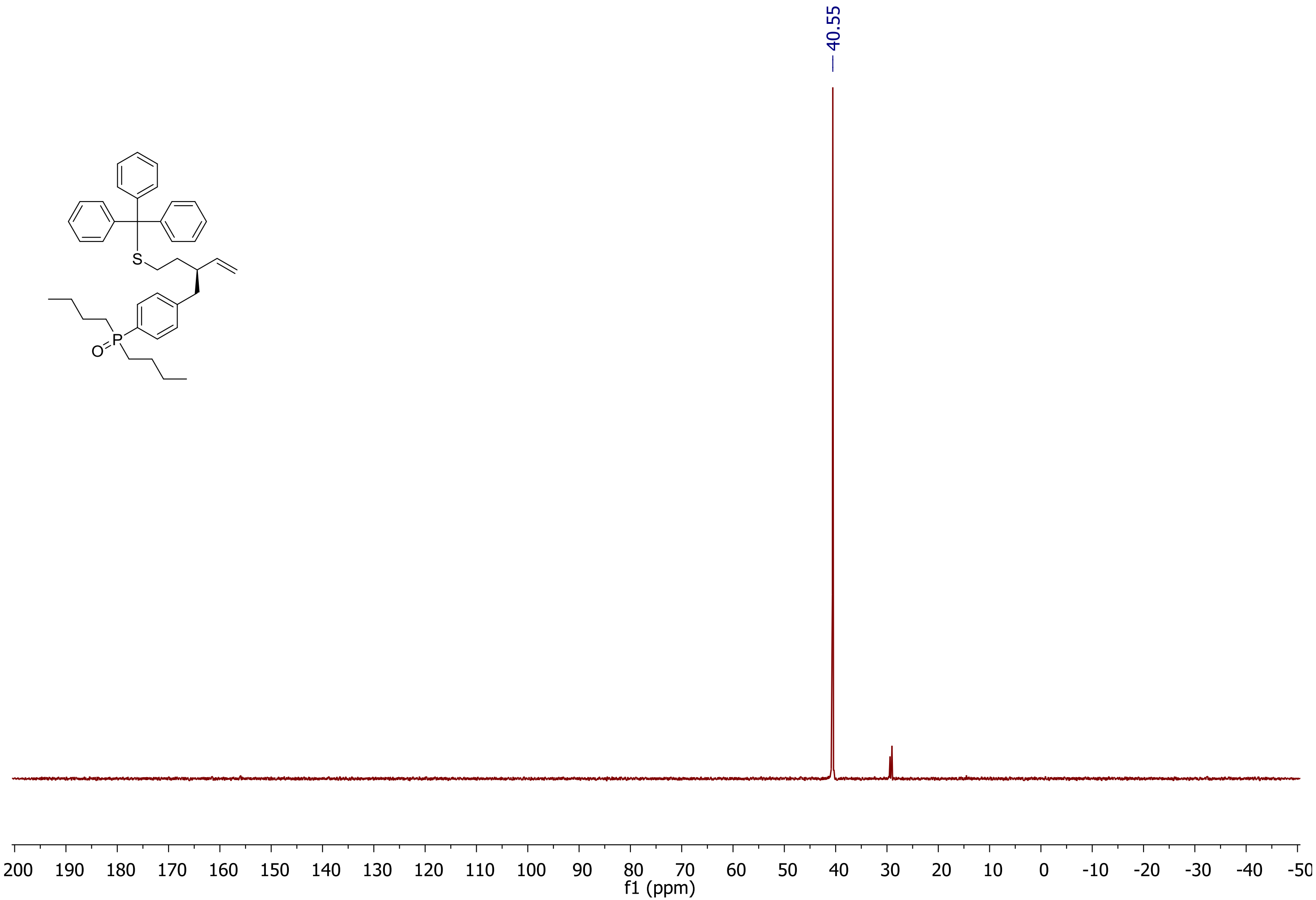

General procedure for the synthesis of 11a and 12b.

A solution of trityl derivative **10a** or **10b** (0.577 mmol) in CH_2Cl_2 (1 mL) was treated with a solution of 5% TFA and 2.5% Et_3SiH in CH_2Cl_2 (0.15 M, 7.69 mL) and stirred at room temperature for 1 h. Then, the solvent was evaporated to dryness, co-evaporating twice with CH_2Cl_2. The obtained residue was dissolved in dry CH_2Cl_2 (5 mL), and triethylamine (0.201 mL, 1.44 mmol) and acetyl chloride (0.082 mL, 1.15 mmol) were sequentially added at 0 ^{o}C. The resulting solution was stirred at room temperature for 2 h and the reaction mixture was directly subjected to silica gel column chromatography.

Compound 11a.

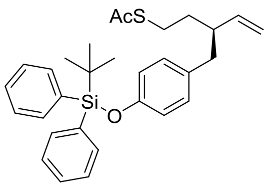

Compound **11a** was obtained from compound **10a** following the general procedure above as a syrup (yield: 85%) using EtOAc:hexane 1:99 as the solvent system for flash chromatography.

$[\alpha]_D^{20}$: +20.4 (*c* 0.79, $CHCl_3$).

^{1}H NMR (400 MHz, $CDCl_3$): δ_H = 7.71 (m, 4H), 7.45-7.33 (m, 6H), 6.84 (d, 2H, *J* = 8.5 Hz), 6.66 (d, 2H, *J* = 8.5 Hz), 5.52 (ddd, 1H, *J* = 17.0, 10.5, 9.0 Hz), 4.96 (dd, 1H, *J* = 10.5, 1.5 Hz), 4.87 (m, 1H), 2.92 (m, 1H), 2.69 (m, 1H), 2.50 (m, 2H), 2.30 (s, 3H), 2.26 (m, 1H), 1.60 (m, 1H), 1.48 (m, 1H), 1.10 (s, 9H).

^{13}C NMR (100.6 MHz, $CDCl_3$): δ_C = 196.0, 153.9, 141.1, 135.7, 133.3, 132.5, 130.0, 129.9, 127.8, 119.5, 115.9, 45.4, 41.0, 33.7, 30.8, 27.2, 26.7, 19.6.

MS (ES+): m/z (%) = 511.3 $[M+Na]^+$.

HRMS (ES+): calcd for $C_{30}H_{36}O_2SiSNa$ 511.2103 $[M+Na]^+$, found 511.2127 $[M+Na]^+$.

FT-IR (ATR): 2934, 2857, 1692, 1509, 1254, 1113, 916 ν_{max}/cm^{-1}.

^{1}H NMR (400 MHz, $CDCl_3$) compound 11a.

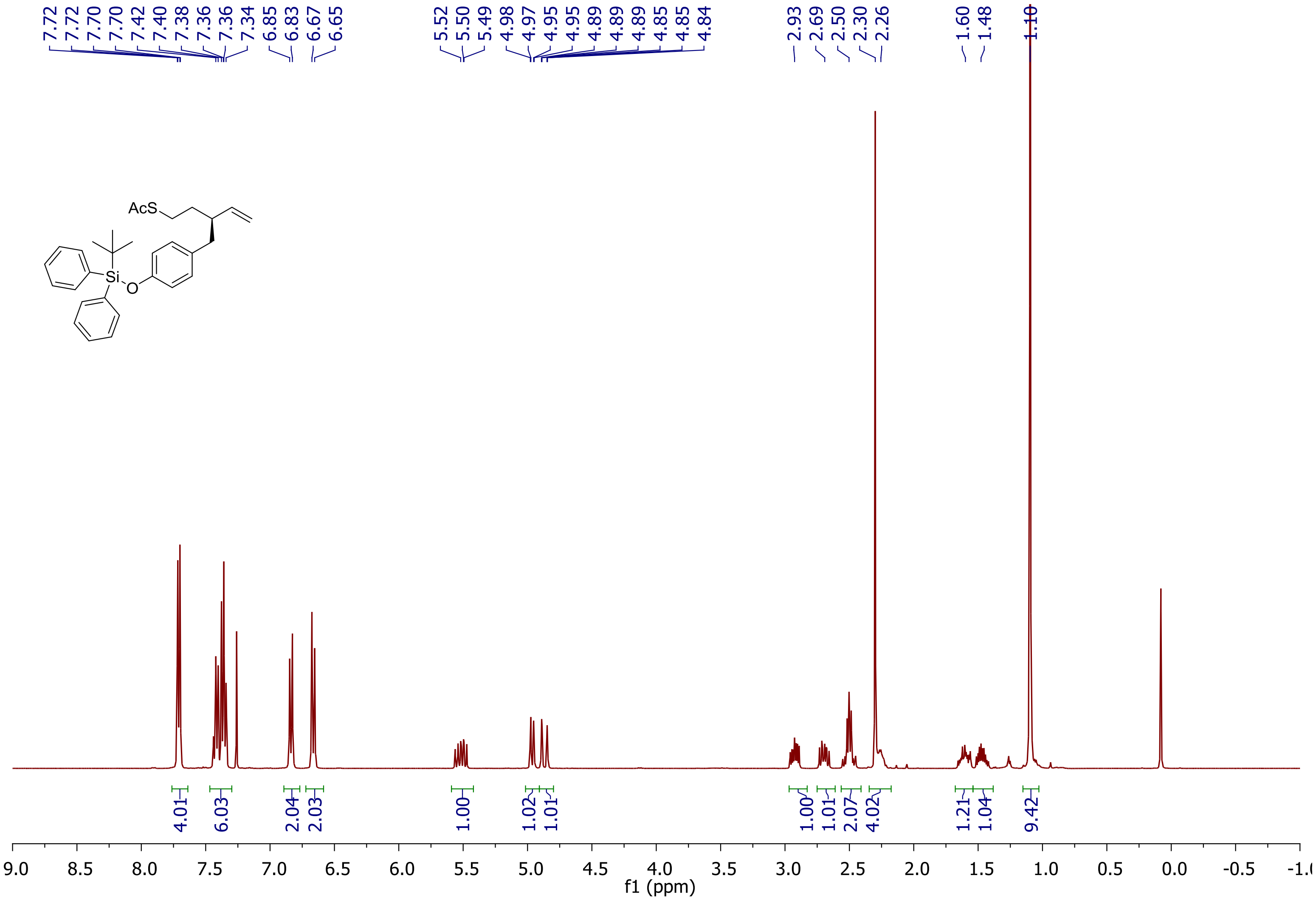

^{13}C NMR (100.6 MHz, $CDCl_3$) compound 11a.

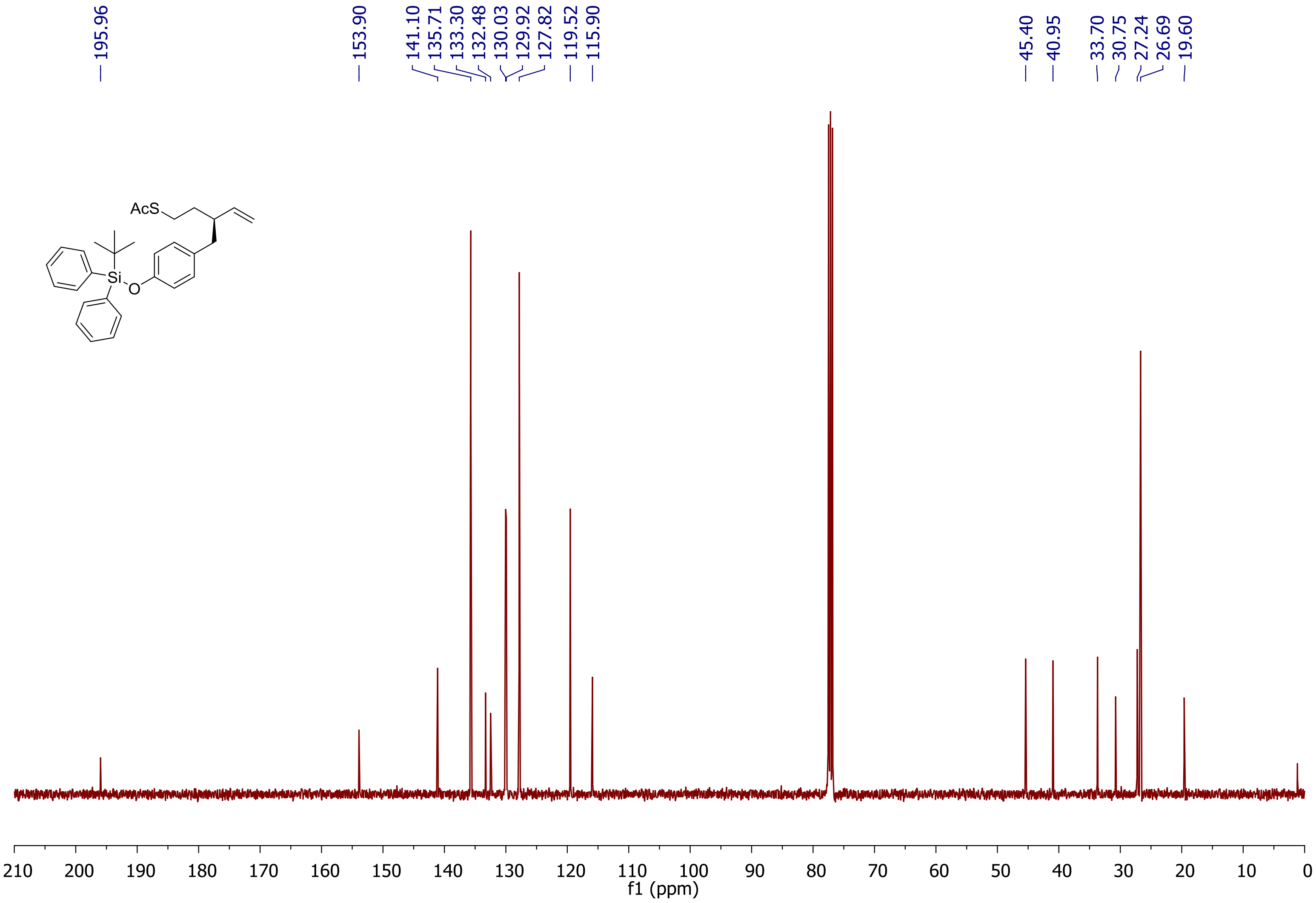

Compound 12b.

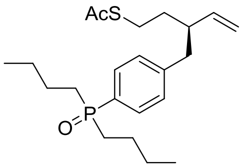

Compound **12b** was obtained from compound **10b** following the general procedure described before as syrup (yield: 87%) using CH_2Cl_2:MeOH 49:1 as the solvent system for flash chromatography.

$[\alpha]_D^{20}$: +21.6 (*c* 1.39, $CHCl_3$).

^{1}H NMR (400 MHz, $CDCl_3$): δ_H = 7.58 (dd, 2H, *J* = 10.5, 8.0 Hz), 7.24 (dd, 2H, *J* = 8.0, 2.0 Hz), 5.55 (ddd, *J* = 17.0, 10.0, 9.0 Hz), 5.00 (dd, 1H, *J* = 10.0, 1.5 Hz), 4.90 (m, 1H), 2.95 (m, 1H), 2.69 (m, 3H), 2.39 (m, 1H), 2.31 (s, 3H), 1.89 (m, 4H), 1.61 (m, 4H), 1.37 (m, 6H), 0.86 (t, 6H, *J* = 7.0 Hz).

^{13}C NMR (100.6 MHz, $CDCl_3$): δ_C = 195.9, 143.9, 140.4, 130.4 (d, *J* = 9.0 Hz), 129.7 (d, *J* = 11.5 Hz), 129.5, 116.5, 45.1, 41.7, 34.0, 30.8, 29.8 (d, *J* = 68.5 Hz), 27.1, 24.2 (d, *J* = 14.5 Hz), 23.6 (d, *J* = 4.0 Hz), 13.7.

^{31}P NMR (161.3 MHz, $CDCl_3$): δ_P = 40.8.

MS (ES+): m/z (%) = 395.2 $[M+H]^+$.

HRMS (ES+): calcd for $C_{22}H_{36}O_2PS$ 395.2174 $[M+H]^+$, found 395.2183 $[M+H]^+$.

FT-IR (ATR): 2958, 2930, 2871, 1689, 1165, 1110, 912 ν_{max}/cm^{-1}.

^{1}H NMR (400 MHz, CDCl$_3$) compound 12b.

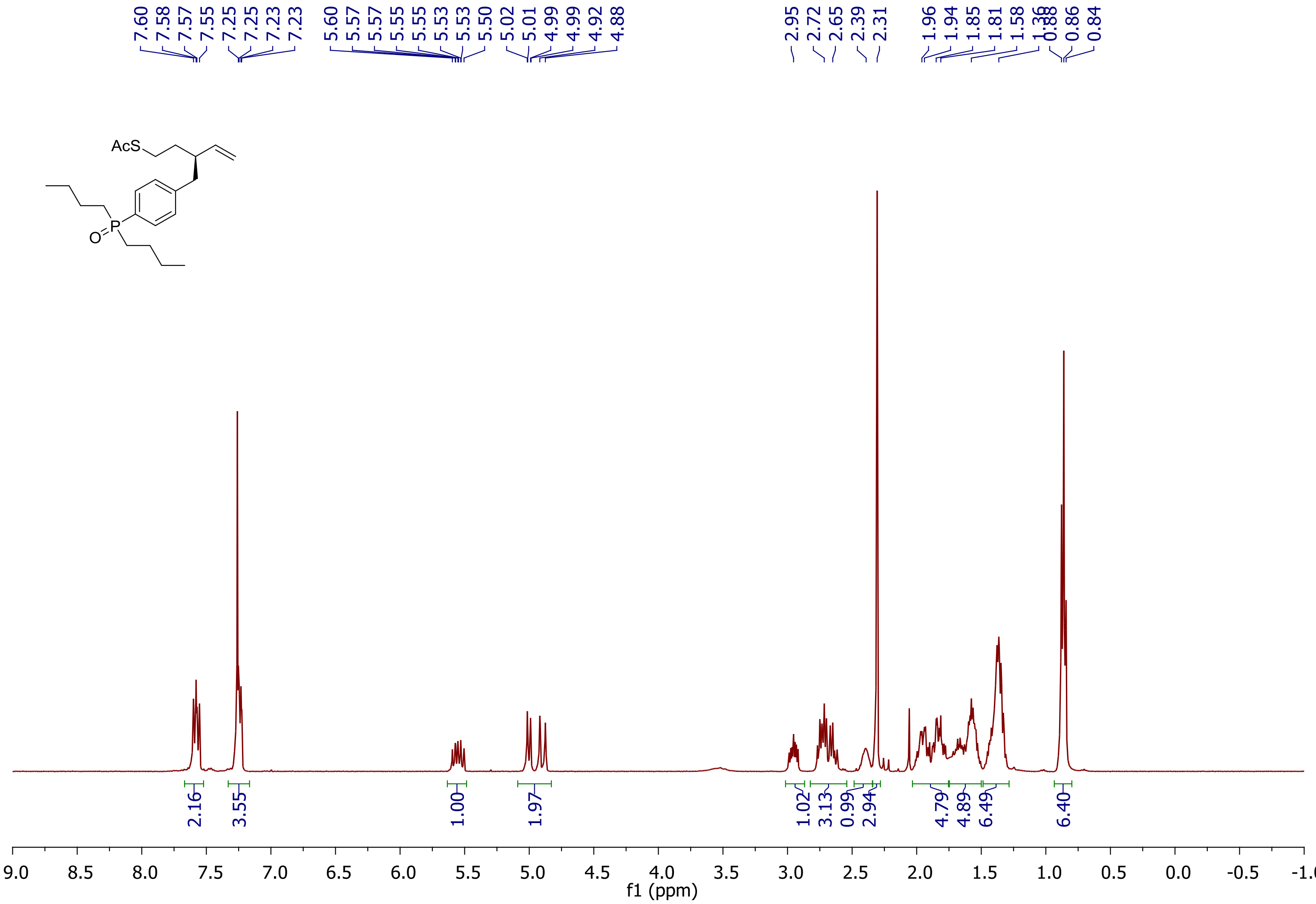

^{13}C NMR (100.6 MHz, $CDCl_3$) compound 12b.

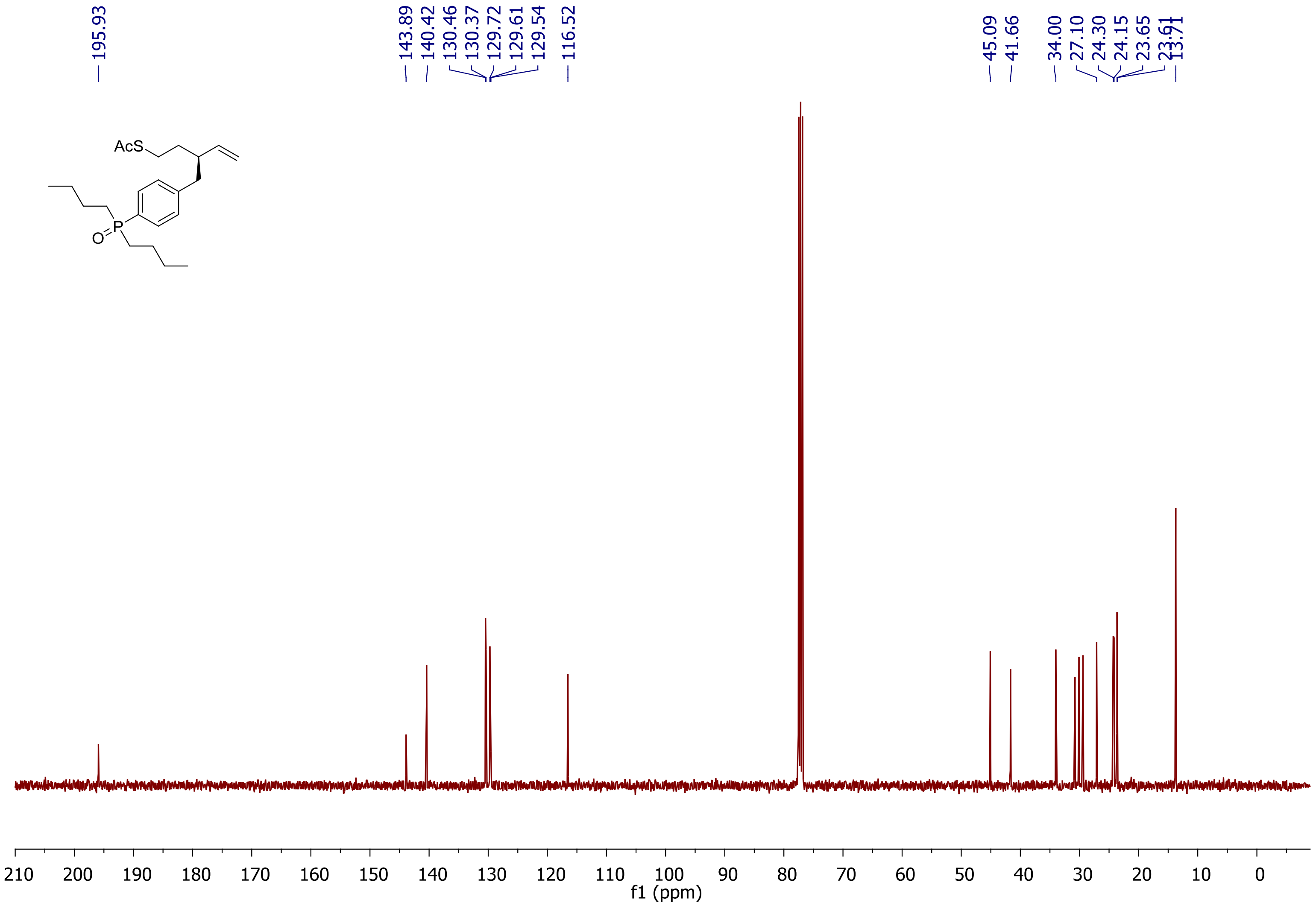

^{31}P NMR (161.3 MHz, $CDCl_3$) compound 12b.

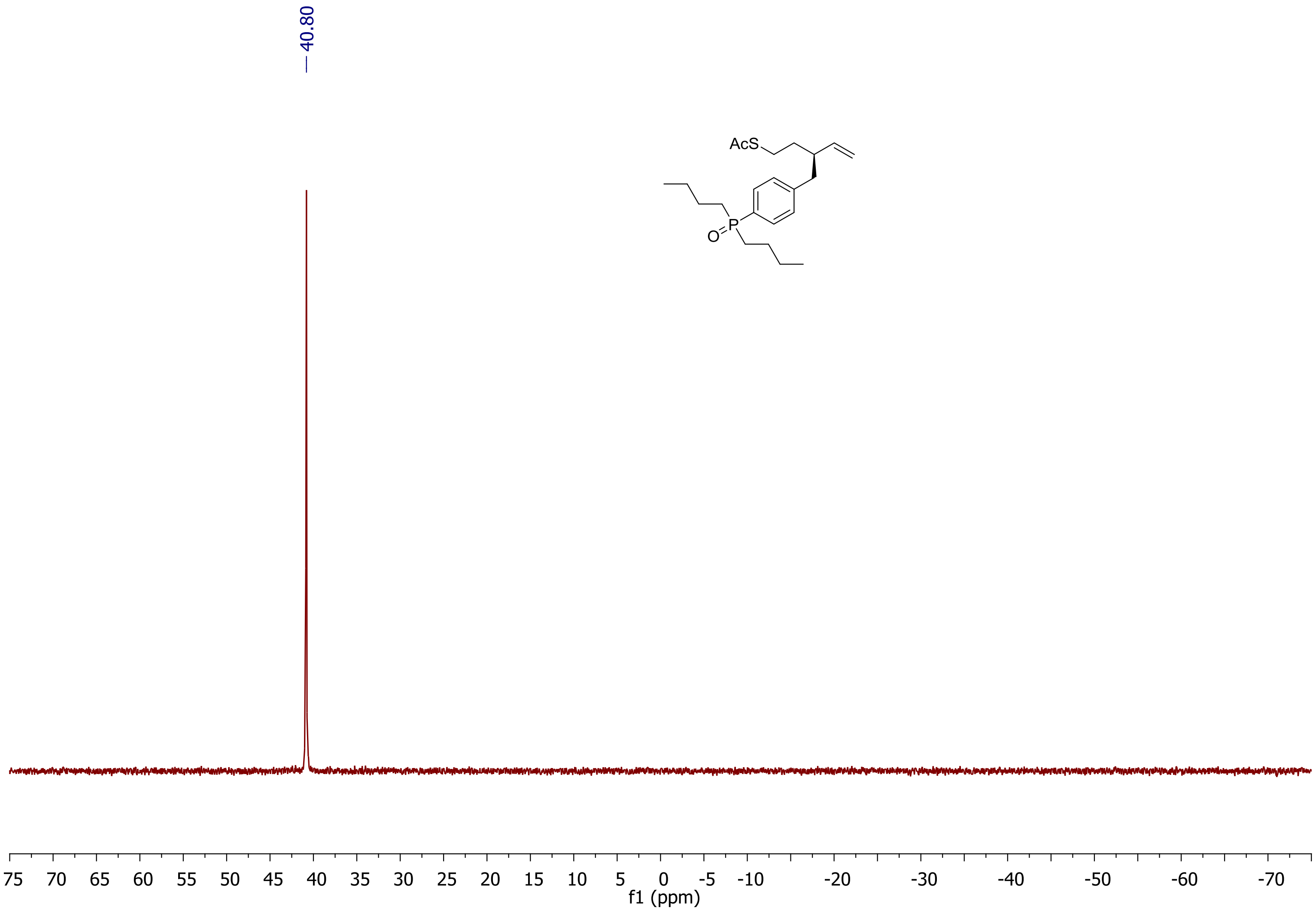

Synthesis of compound 12a.

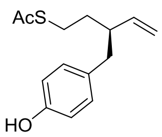

A solution of TBAF in THF (1 M, 3.309 mL, 3.31 mmol) was added dropwise to a solution of **11a** (1.617 g, 3.31 mmol) in THF (20 mL) at 0 °C. After stirring at room temperature for 15 min, H_2O (20 mL) was added and the solution was extracted with EtOAc (3x25 mL). The organic layers were collected and washed with brine, dried over $MgSO_4$ and evaporated to dryness. The obtained residue was purified by flash chromatography (EtOAc:hexane 1:9) to yield **12a** (0.816 g, 99%) as a syrup.

$[\alpha]_D^{20}$: +44.8 (*c* 1.41, $CHCl_3$).

^{1}H NMR (400 MHz, $CDCl_3$): δ_H = 6.98 (d, 2H, *J* = 8.5 Hz), 6.75 (d, 2H, *J* = 8.5 Hz,), 5.56 (ddd, 1H, *J* = 17.0, 10.5, 9.0 Hz), 5.49 (bs, 1H), 5.00 (dd, 1H, *J* = 10.5, 1.5 Hz), 4.93 (m, 1H), 2.96 (m, 1H), 2.73 (m, 1H), 2.57 (m, 2H), 2.31 (s, 3H), 2.30 (m, 1H), 1.65 (m, 1H), 1.52 (m, 1H).

^{13}C NMR (100.6 MHz, $CDCl_3$): δ_C = 196.9 and 196.8 (CO, splitted), 154.0, 141.1, 132.0, 130.4, 116.0, 115.1, 45.4, 40.8, 33.7, 30.7, 27.3.

MS (ES+): m/z (%) = 251.1 $[M+H]^+$.

HRMS (ES+): calcd for $C_{14}H_{19}O_2S$ 251.1106 $[M+H]^+$, found 251.1108 $[M+H]^+$.

FT-IR (ATR): 2920, 1689, 1662, 1514, 1221, 1133, 917, 774 ν_{max}/cm^{-1}.

^{1}H NMR (400 MHz, $CDCl_3$) compound 12a.

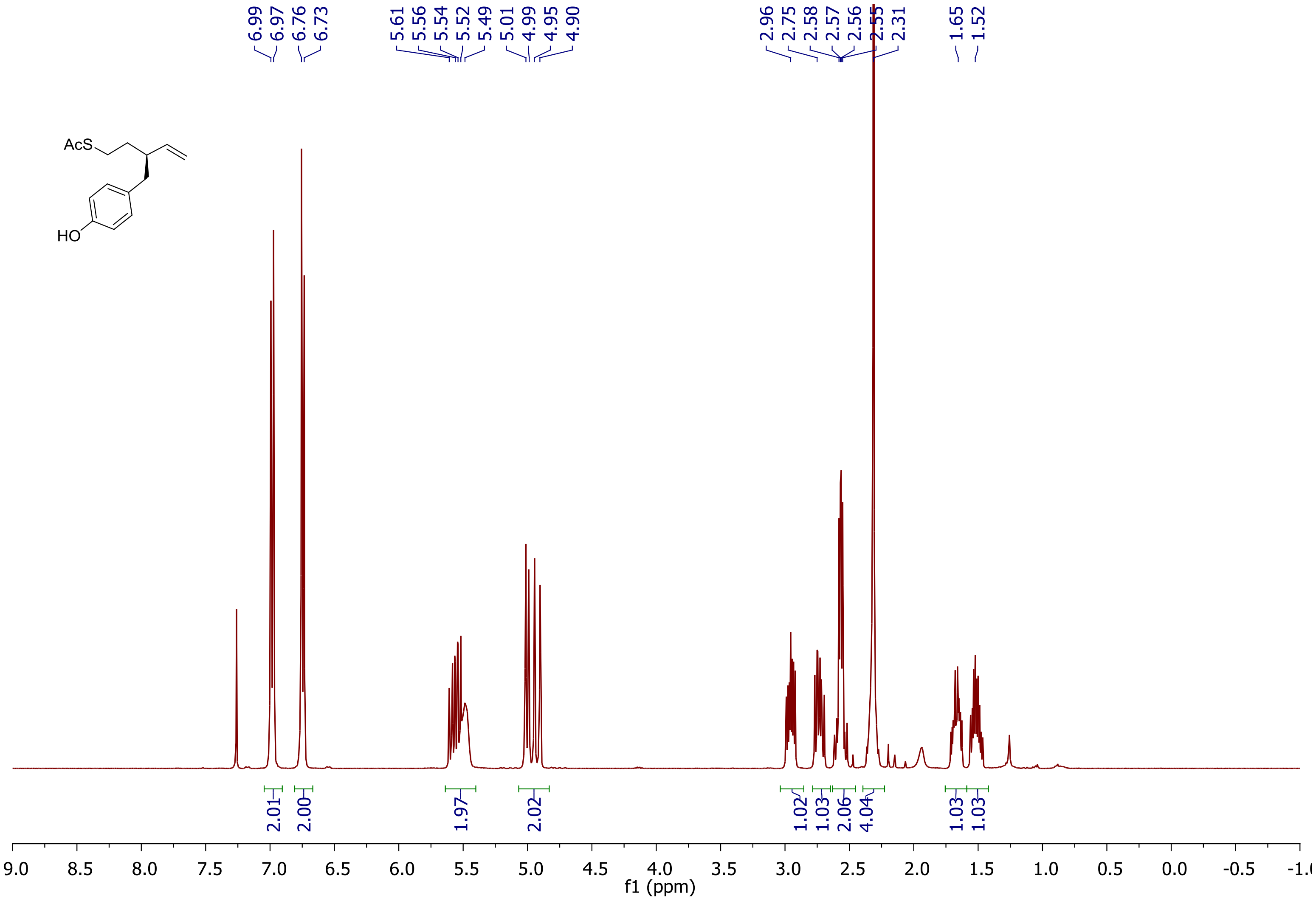

^{13}C NMR (100.6 MHz, $CDCl_3$) compound 12a.

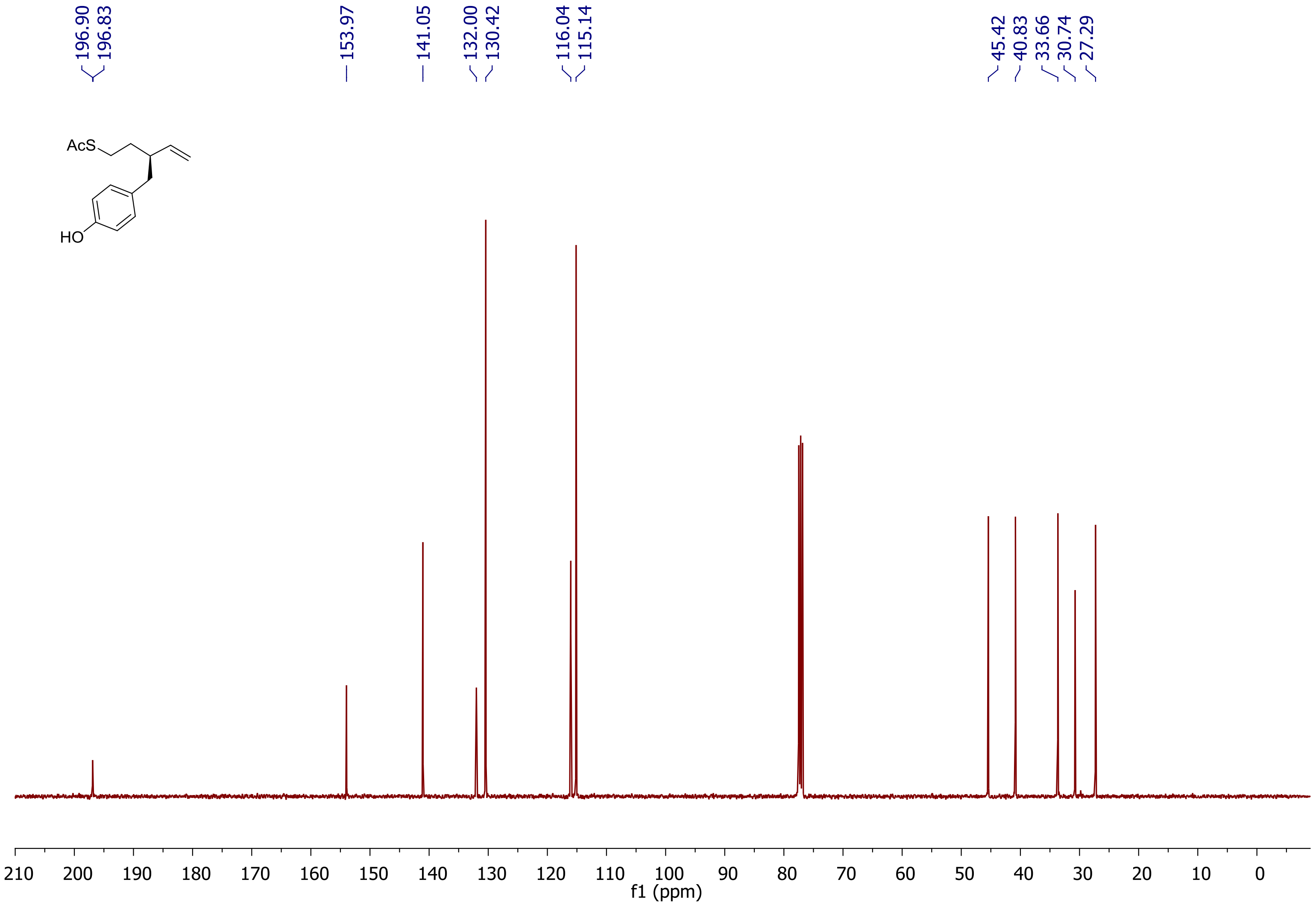

General procedure for the synthesis of compounds 13a and 13b.

To a solution of **12a** or **12b** (0.56 mmol) in dry and degassed MeOH (2.1 mL) under N_2 atmosphere was added DMPA (0.014 g, 0.056 mmol) and 1-hexanethiol (0.238 mL, 1.68 mmol). The reaction was stirred at room temperature under UV irradiation (365 nm) for 30 min. Then, the solvent was removed under vacuum and the crude purified by flash chromatography.

Compound 13a.

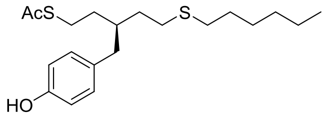

Compound **13a** was obtained from compound **12a** following the general procedure above as a syrup (yield: 92%) using EtOAc:hexane 1:9 as the solvent system for flash chromatography.

$[\alpha]_D^{20}$: -4.2 (*c* 0.71, $CHCl_3$).

^{1}H NMR (400 MHz, $CDCl_3$): δ_H = 7.01 (d, 2H, *J* = 8.5 Hz), 6.75 (d, 2H, *J* = 8.5 Hz), 4.94 (bs, 1H), 2.87 (m, 2H), 2.51 (m, 4H), 2.44 (m, 2H), 2.31 (s, 3H), 1.81 (m, 1H), 1.55 (m, 6H), 1.31 (m, 6H), 0.88 (t, 3H, *J* = 7.0 Hz).

^{13}C NMR (100.6 MHz, $CDCl_3$): δ_C = 196.3, 154.0, 132.5, 130.4, 115.3, 39.2, 38.8, 33.1, 33.0, 32.2, 31.6, 30.8, 29.8, 29.5, 28.8, 26.9, 22.7, 14.2.

MS (ES+): m/z (%) = 369.2 $[M+H]^+$.

HRMS (ES+): calcd for $C_{20}H_{33}O_2S_2$ 369.1922 $[M+H]^+$, found 369.1929 $[M+H]^+$.

FT-IR (ATR): 2922, 2854, 1689, 1514, 1214, 1130, 956, 823 ν_{max}/cm^{-1}.

^{1}H NMR (400 MHz, $CDCl_3$) compound 13a.

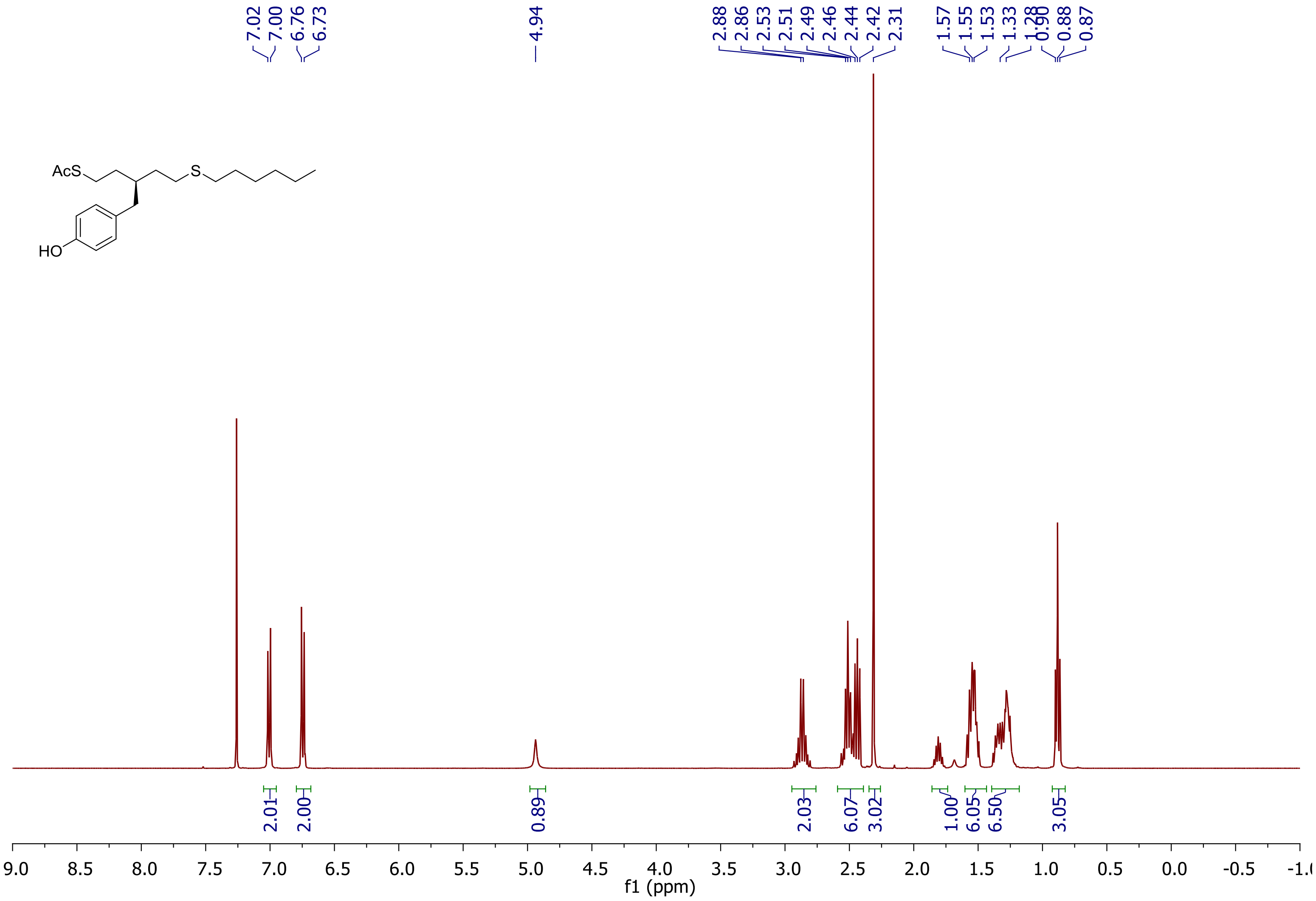

^{13}C NMR (100.6 MHz, $CDCl_3$) compound 13a.

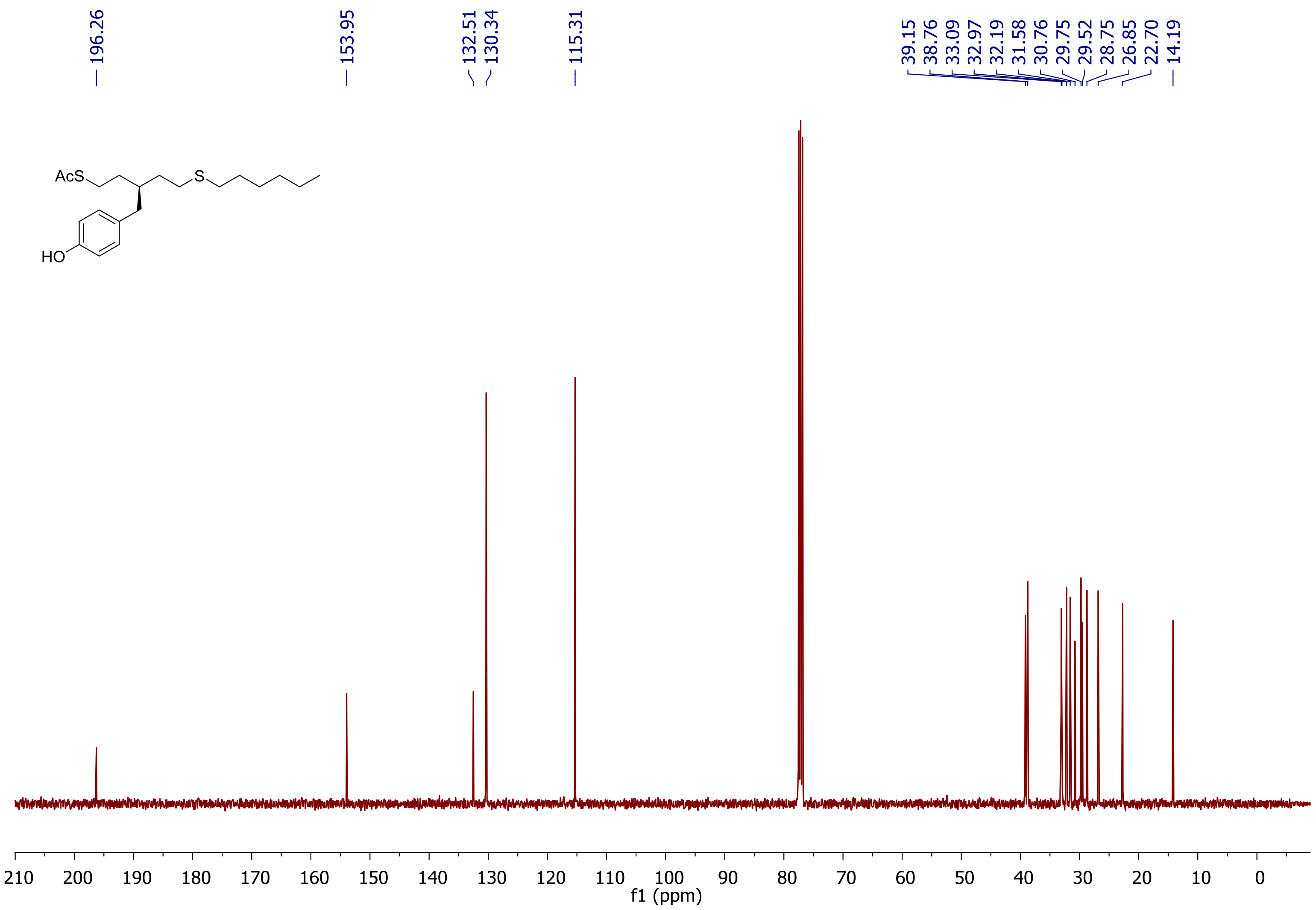

Compound 13b.

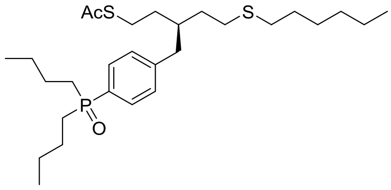

Compound **13b** was obtained from compound **12b** following the general procedure described before as syrup (yield: 74%) using CH_2Cl_2:MeOH 30:1 as the solvent system for flash chromatography.

$[\alpha]_D^{20}$: -17.9 (*c* 1.12, $CHCl_3$).

^{1}H NMR (400 MHz, $CDCl_3$): δ_H = 7.60 (dd, 2H, *J* = 10.5, 8.0 Hz), 7.28 (m, 2H), 2.86 (m, 2H), 2.64 (m, 2H), 2.52 (m, 2H), 2.44 (m, 2H), 2.31 (s, 3H), 1.87 (m, 5H), 1.56 (m, 8H), 1.34 (m, 12H), 0.87 (m, 9H).

^{13}C NMR (100.6 MHz, $CDCl_3$): δ_C = 195.8, 144.3, 130.7 (d, *J* = 9.0 Hz), 130.3 (d, *J* = 93.5 Hz), 129.6 (d, *J* = 11.5 Hz), 40.1, 38.4, 33.2, 33.0, 32.3, 31.6, 30.8, 29.9 (d, *J* = 68.5 Hz), 29.7, 29.5, 28.7, 26.7, 24.3 (d, *J* = 14.5 Hz), 23.7 (d, *J* = 4.0 Hz), 22.7, 14.2, 13.7.

^{31}P NMR (161.3 MHz, $CDCl_3$): δ_P = 40.4.

MS (ES+): m/z (%) = 369.2 $[M+H]^+$.

HRMS (ES+): calcd for $C_{28}H_{50}O_2PS_2$ 513.2990 $[M+H]^+$, found 513.3010 $[M+H]^+$.

FT-IR (ATR): 2954, 2929, 2860, 1691, 1169, 1111, 958 ν_{max}/cm^{-1}.

^{1}H NMR (400 MHz, $CDCl_3$) compound 13b.

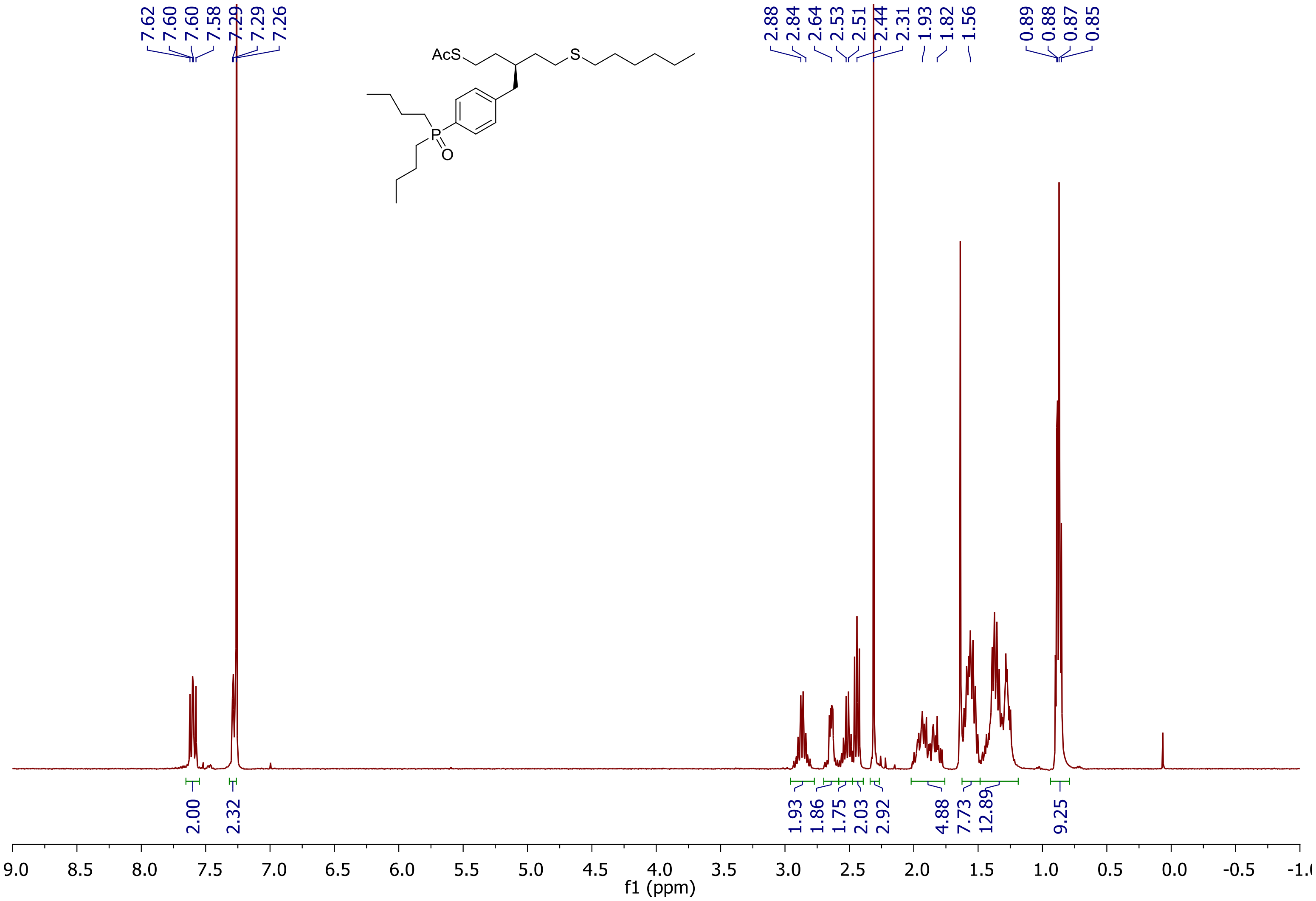

^{13}C NMR (100.6 MHz, $CDCl_3$) compound 13b.

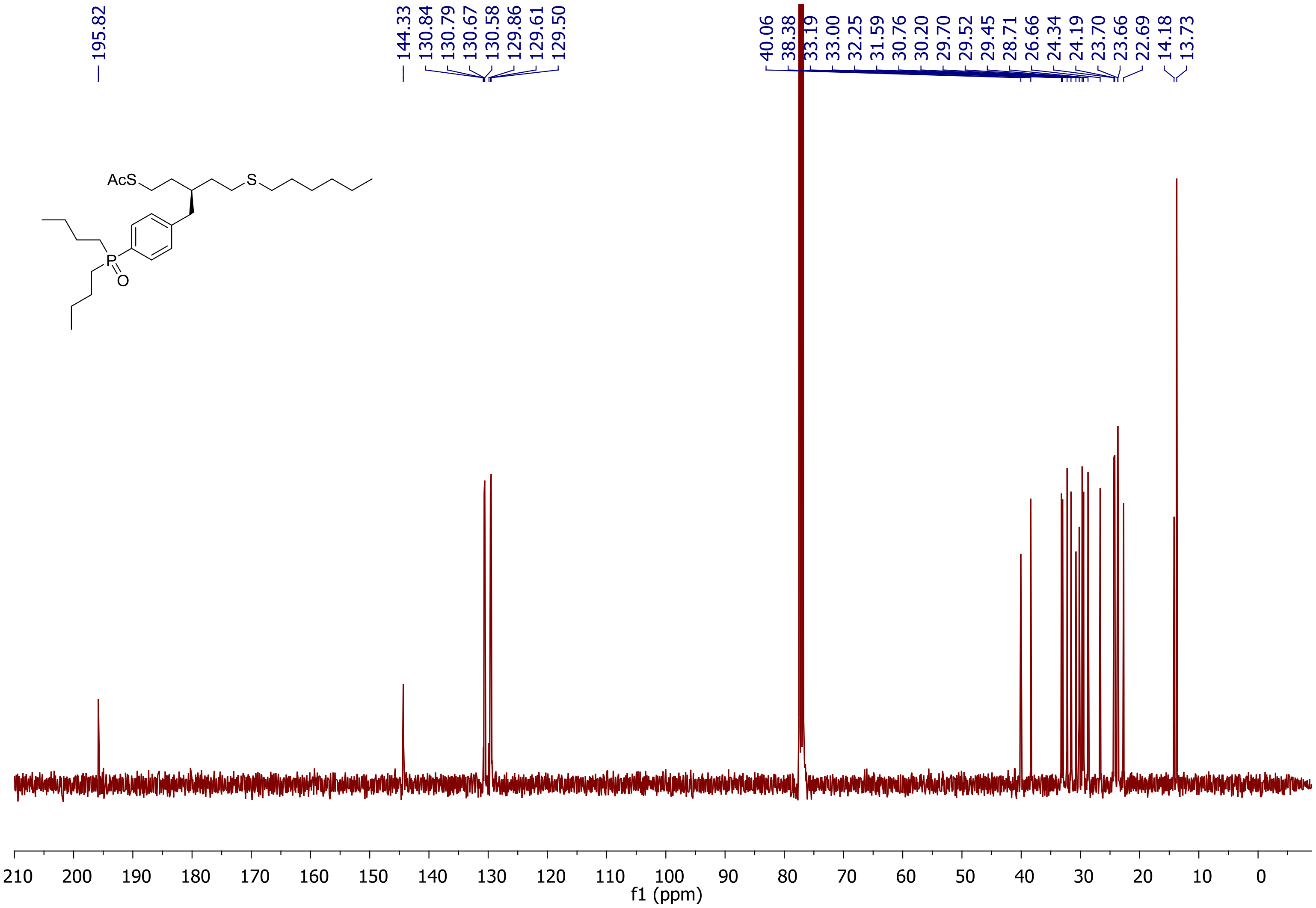

^{31}P NMR (161.3 MHz, $CDCl_3$) compound 13b.

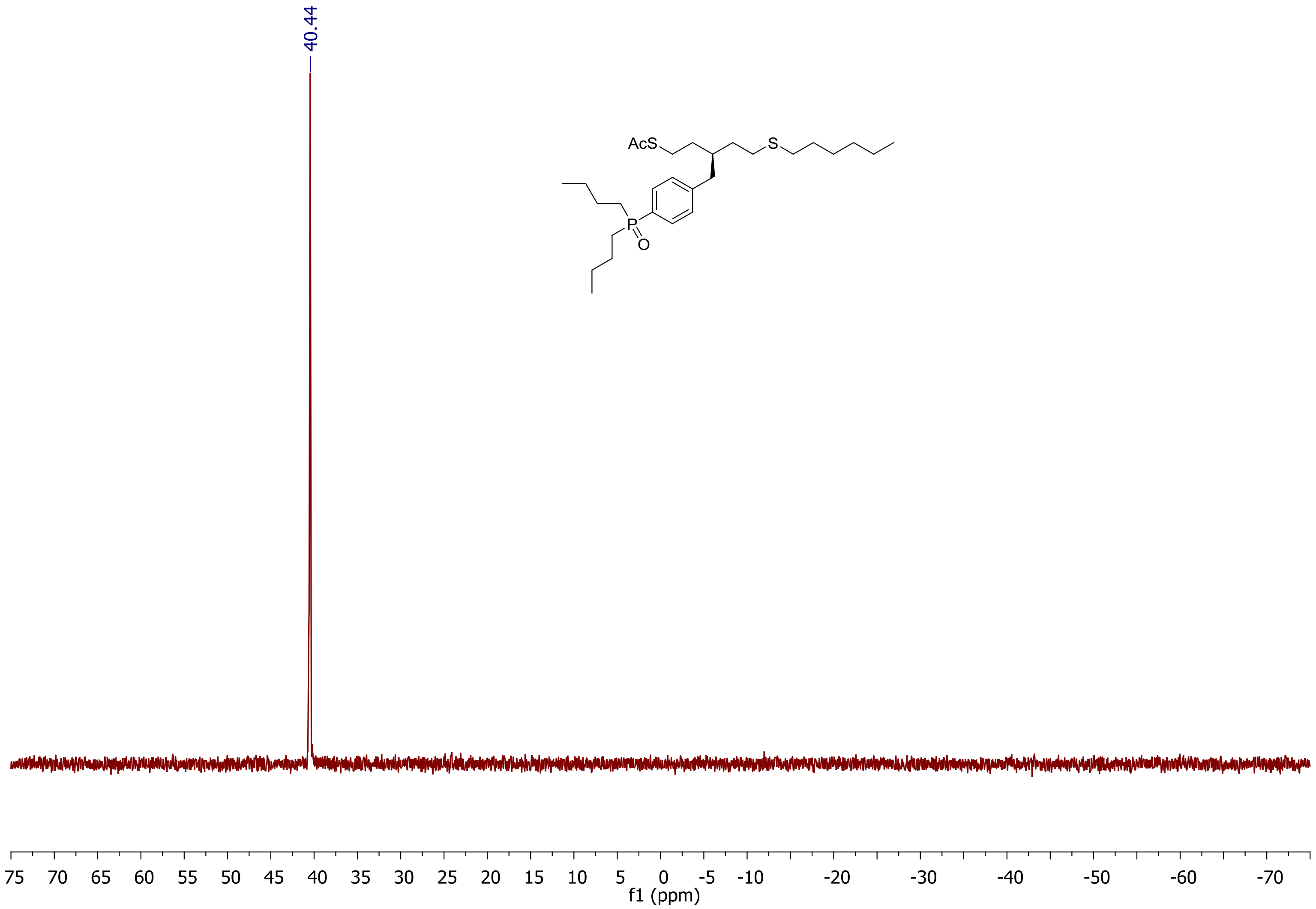

General procedure for the synthesis of oligomers 14-16 through thiol-ene coupling.

Method A: A solution of *S*-acetyl protected derivative (0.191 mmol) in MeOH (4 mL) was treated with 2 N NaOH solution (0.287 mL, 0.573 mmol). After stirring at room temperature for 30 min, the reaction was quenched with diluted HCl solution (pH = 3-4) and extracted with EtOAc (3x10 mL). The organic phase was dried over $MgSO_4$, evaporated and dried under high vacuum for 30 min. The obtained residue was dissolved in dry and degassed MeOH (1.1 mL) under N_2 atmosphere. DMPA (0.005 g, 0.019 mmol) and monomer **12a** or **12b** (0.048 g, 0.191 mmol) were added and the reaction was stirred at room temperature under UV irradiation (365 nm) for the specified time. Then, the solvent was removed under vacuum and the crude purified by flash chromatography.

Method B: A solution of **15b** (0.029 g, 0.024 mmol) in MeOH (2 mL) was treated with 2 N NaOH solution (0.048 mL, 0.095 mmol). After stirring at room temperature for 30 min, the reaction was quenched with 0.1 M HCl solution (pH = 3-4) and extracted with EtOAc (3x10 mL). The organic phase was dried over $MgSO_4$ and evaporated to dryness. The reaction yielded the corresponding disulfide so the crude was dissolved in dry CH_2Cl_2 (2 mL) under N_2 atmosphere, and dithiothreitol (0.006 g, 0.036 mmol) and TEA (0.005 mL, 0.036 mmol) were added. After 48 of stirring at room temperature, the reaction was diluted with CH_2Cl_2 (4 mL) and washed with 5% citric acid solution (2x2 mL), H_2O (2x2 mL) and brine (1x2 mL). The organic phase was dried over $MgSO_4$, evaporated and dried under high vacuum for 30 min. The obtained residue was dissolved in dry and degassed MeOH (0.3 mL) under N_2 atmosphere. DMPA (0.0005 g, 0.002 mmol) and **12b** (0.010 g, 0.018 mmol) were added and the reaction was stirred at room temperature under UV irradiation (365 nm) for 2 h. Then, the solvent was removed under vacuum and the crude purified by flash chromatography.

Compound 14a.

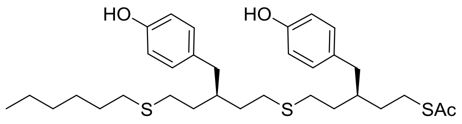

Compound **14a** was obtained from compound **13a** following the general procedure above (Method A, UV irradiation for 1.5 h) as a syrup (yield: 77%) using EtOAc:hexane 1:4 as the solvent system for flash chromatography.

$[\alpha]_D^{20}$: -2.2 (*c* 1.35, $CHCl_3$).

^{1}H NMR (400 MHz, $CDCl_3$): δ_H = 6.99 (m, 4H), 6.75 (m, 4H), 5.34 (bs, 1H), 5.22 (bs, 1H), 2.87 (m, 2H), 2.44 (m, 12H), 2.33 (s, 3H), 1.76 (m, 2H), 1.40 (m, 16H), 0.89 (t, 3H, *J* = 7.0 Hz).

^{13}C NMR (100.6 MHz, $CDCl_3$): δ_C = 196.9, 154.0, 154.0, 132.6, 132.5, 130.3, 115.4, 115.3, 39.3, 39.1, 38.9, 38.7, 33.4, 33.4, 33.0, 32.9, 32.3, 31.6, 30.8, 29.7, 29.3, 29.2, 28.8, 27.0, 22.7, 14.2.

MS (ES+): m/z (%) = 577.3 $[M+H]^+$.

HRMS (ES+): calcd for $C_{32}H_{49}O_3S_3$ 577.2844 $[M+H]^+$, found 577.2847 $[M+H]^+$.

FT-IR (ATR): 3391, 2922, 2854, 1688, 1662, 1513, 1445, 1219, 824, 773 ν_{max}/cm^{-1}.

^1H NMR (400 MHz, $CDCl_3$) compound 14a.

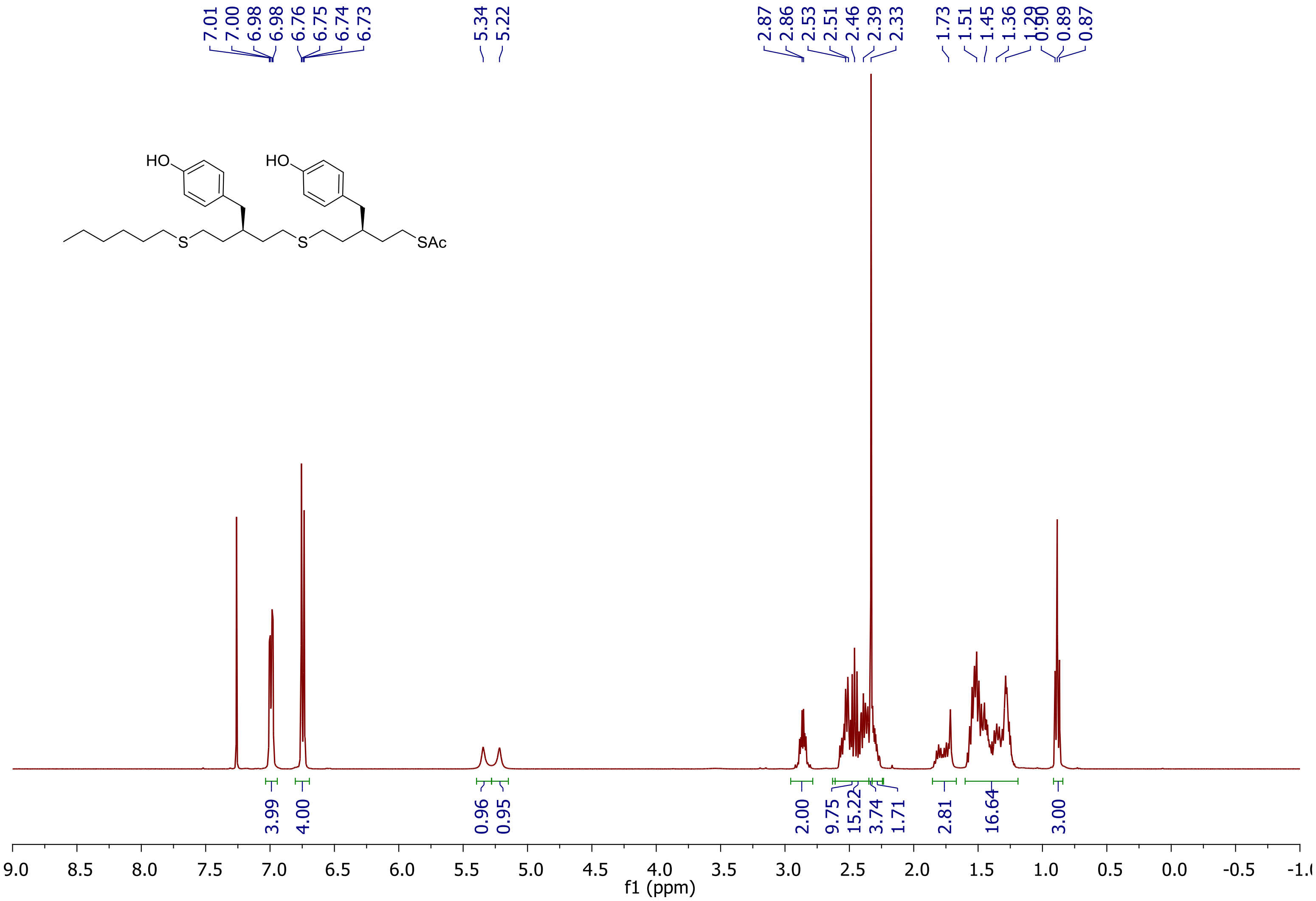

^{13}C NMR (100.6 MHz, CDCl$_3$) compound 14a.

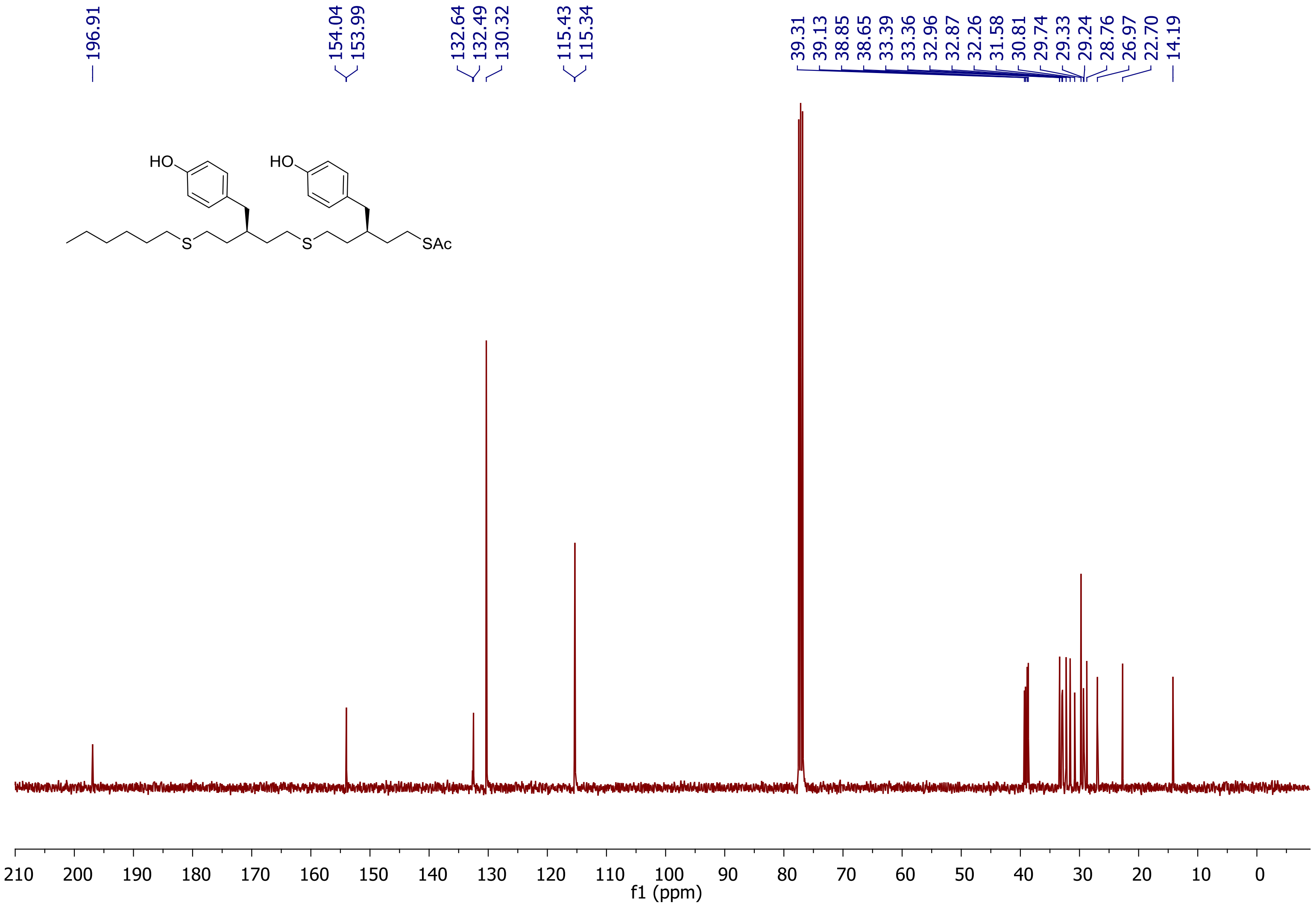

Compound 14b.

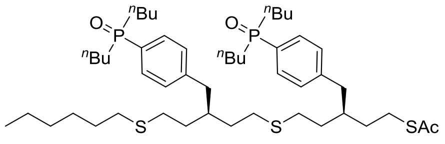

Compound **14b** was obtained from compound **13b** following the general procedure described before (Method A, UV irradiation for 1 h) as syrup (yield: 66%) using CH_2Cl_2:MeOH 25:1 as the solvent system for flash chromatography.

$[\alpha]_D^{20}$: -2.1 (*c* 0.97, $CHCl_3$).

^{1}H NMR (400 MHz, $CDCl_3$): δ_H = 7.60 (m, 4H), 7.26 (m, 4H), 2.83 (m, 2H), 2.50 (m, 12H), 2.30 (s, 3H), 1.91 (m, 10H), 1.54 (m, 14H), 1.31 (m, 18H), 0.86 (m, 15H).

^{13}C NMR (100.6 MHz, $CDCl_3$): δ_C = 195.8, 144.5, 144.2, 130.8, 130.7, 130.6, 130.6, 130.6, 129.8, 129.6, 129.5, 40.1, 40.0, 38.3, 38.2, 33.1, 33.1, 33.0, 32.9, 32.2, 31.6, 30.8, 30.1, 29.7, 29.6, 29.5, 29.5, 28.7, 26.6, 24.3, 23.7, 22.7, 14.2, 13.7.

^{31}P NMR (161.3 MHz, $CDCl_3$): δ_P = 40.4, 40.5.

MS (ES+): m/z (%) = 866.1[M+H]$^+$.

HRMS (ES+): calcd for $C_{48}H_{83}O_3P_2S_3$ 865.4980 [M+H]$^+$, found 865.5005 [M+H]$^+$.

FT-IR (ATR): 2955, 2926, 2859, 1689, 1453, 1166, 1111, 900, 795 ν_{max}/cm^{-1}.

^{1}H NMR (400 MHz, $CDCl_3$) compound 14b.

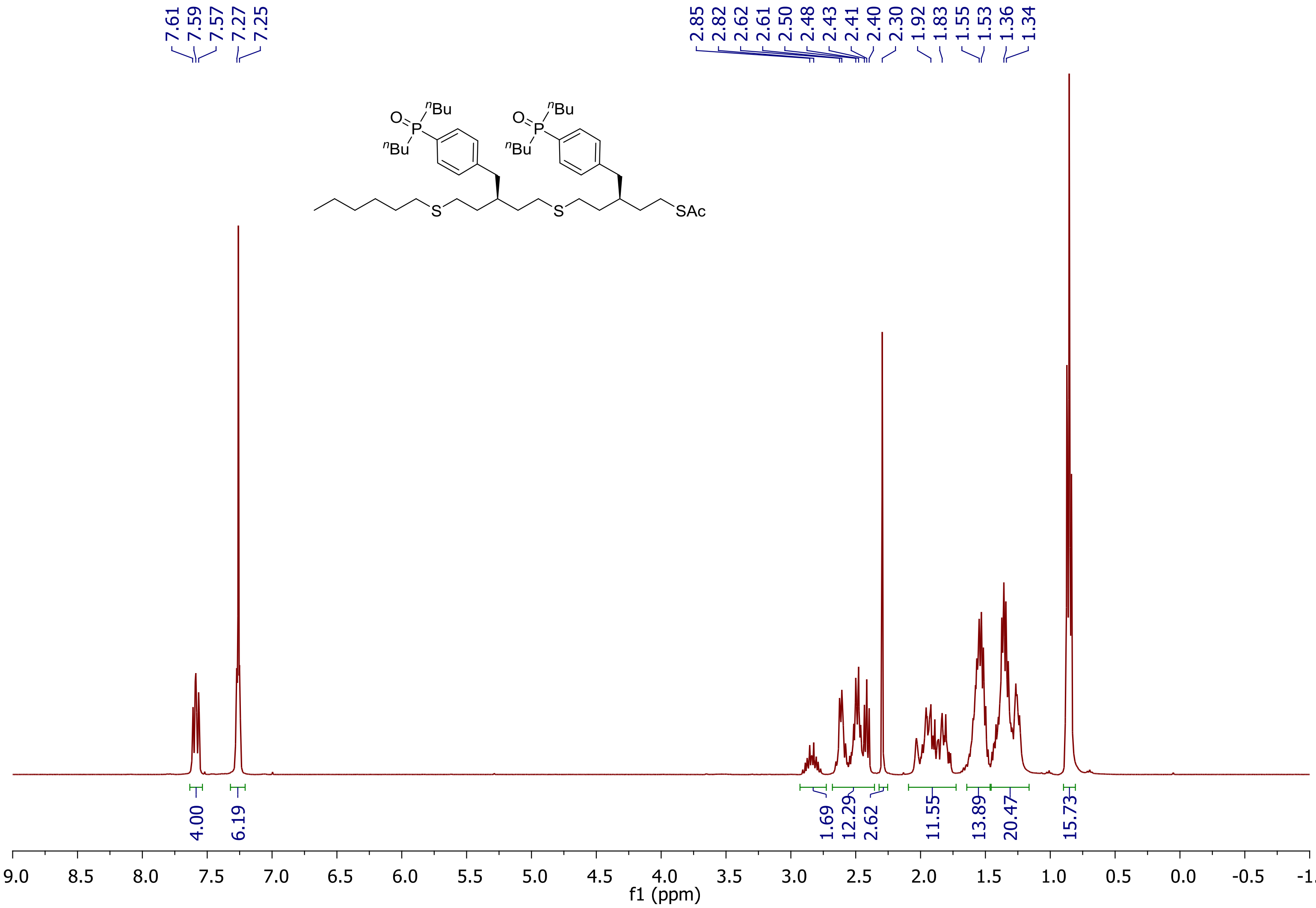

^{13}C NMR (100.6 MHz, CDCl$_3$) compound 14b.

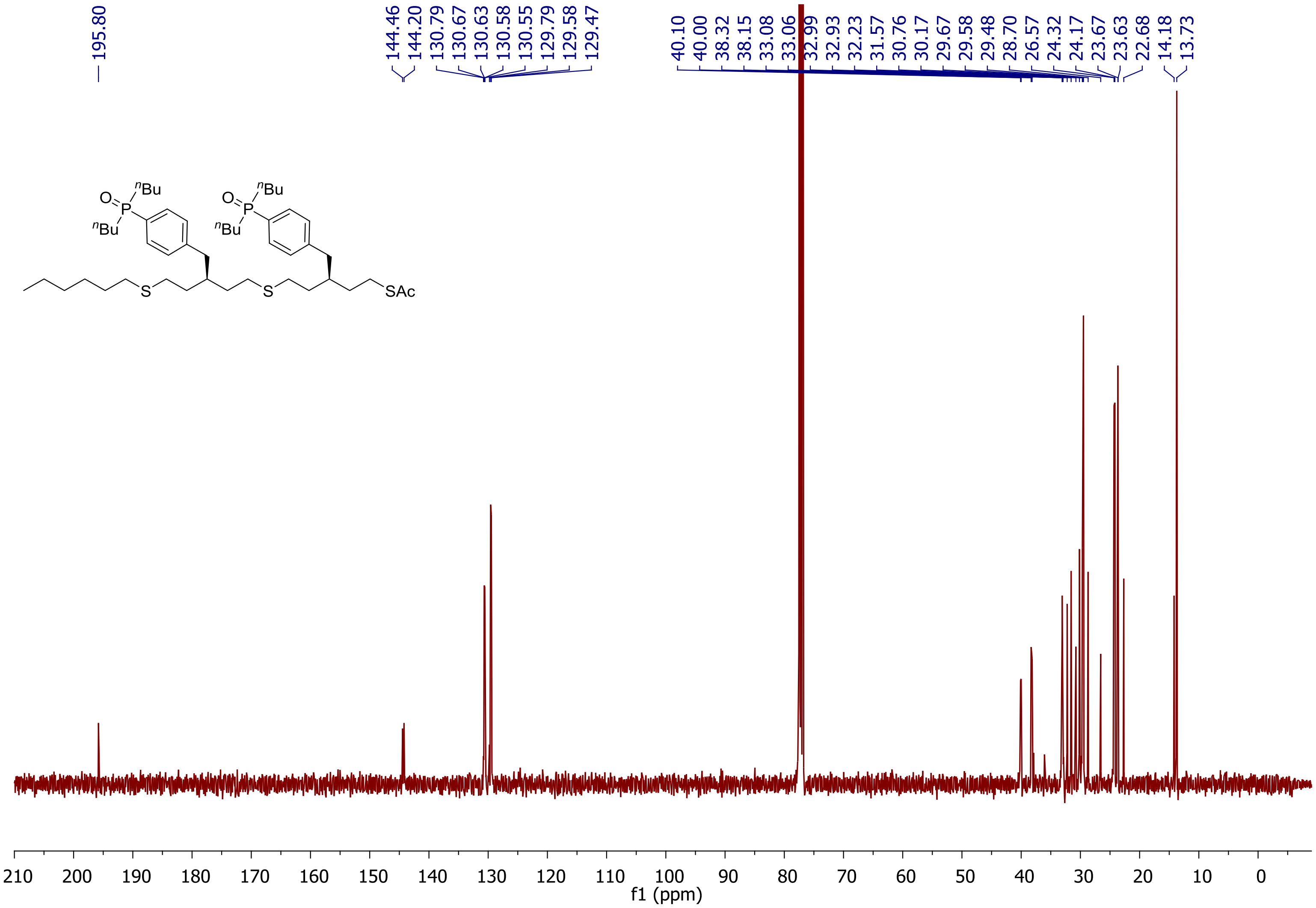

^{31}P NMR (161.3 MHz, $CDCl_3$) compound 14b.

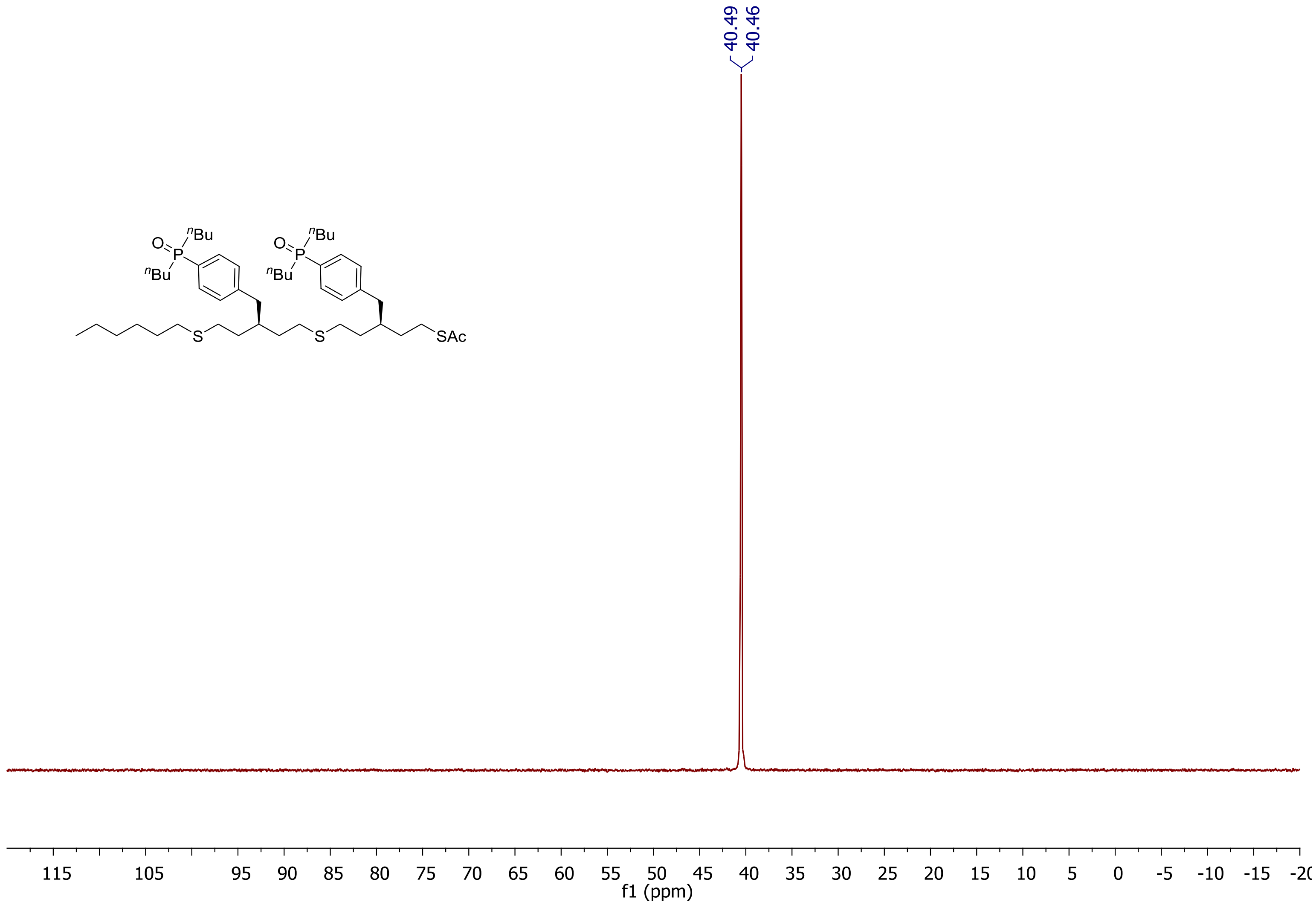

Compound 15a.

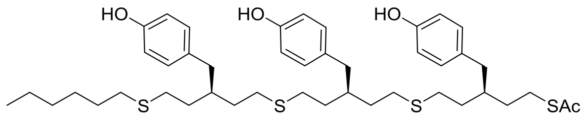

Compound **15a** was obtained from compound **14a** following the general procedure described before (Method A, UV irradiation for 2h) as syrup (yield: 88%) using EtOAc:hexane 1:2 as the solvent system for flash chromatography.

$[\alpha]_D^{20}$: -2.1 (*c* 0.97, $CHCl_3$).

^{1}H NMR (400 MHz, $CDCl_3$): δ_H = 6.98 (m, 6H), 6.74 (m, 6H), 5.95 (bs, 3H), 2.87 (m, 2H), 2.41 (m, 18H), 2.33 (s, 3H), 1.82 (m, 3H), 1.39 (m, 20H), 0.88 (t, 3H, *J* = 7.0 Hz).

^{13}C NMR (100.6 MHz, $CDCl_3$): δ_C = 197.5, 154.1, 154.0, 154.0, 132.6, 132.6, 132.4, 130.3, 130.3, 130.3, 115.4, 115.4, 115.4, 39.3, 39.3, 39.1, 38.8, 38.6, 38.6, 33.4, 33.3, 33.1, 33.0, 32.9, 32.9, 32.3, 31.6, 30.8, 29.7, 29.4, 29.4 29.2, 28.7, 27.0, 22.7, 14.2.

MS (ES+): m/z (%) = 785.4 $[M+H]^+$.

HRMS (ES+): calcd for $C_{44}H_{65}O_4S_4$ 785.3766 $[M+H]^+$, found 785.3768 $[M+H]^+$.

FT-IR (ATR): 3340, 2921, 1613, 1513, 1444, 1233, 825 ν_{max}/cm^{-1}.

^{1}H NMR (400 MHz, $CDCl_3$) compound 15a.

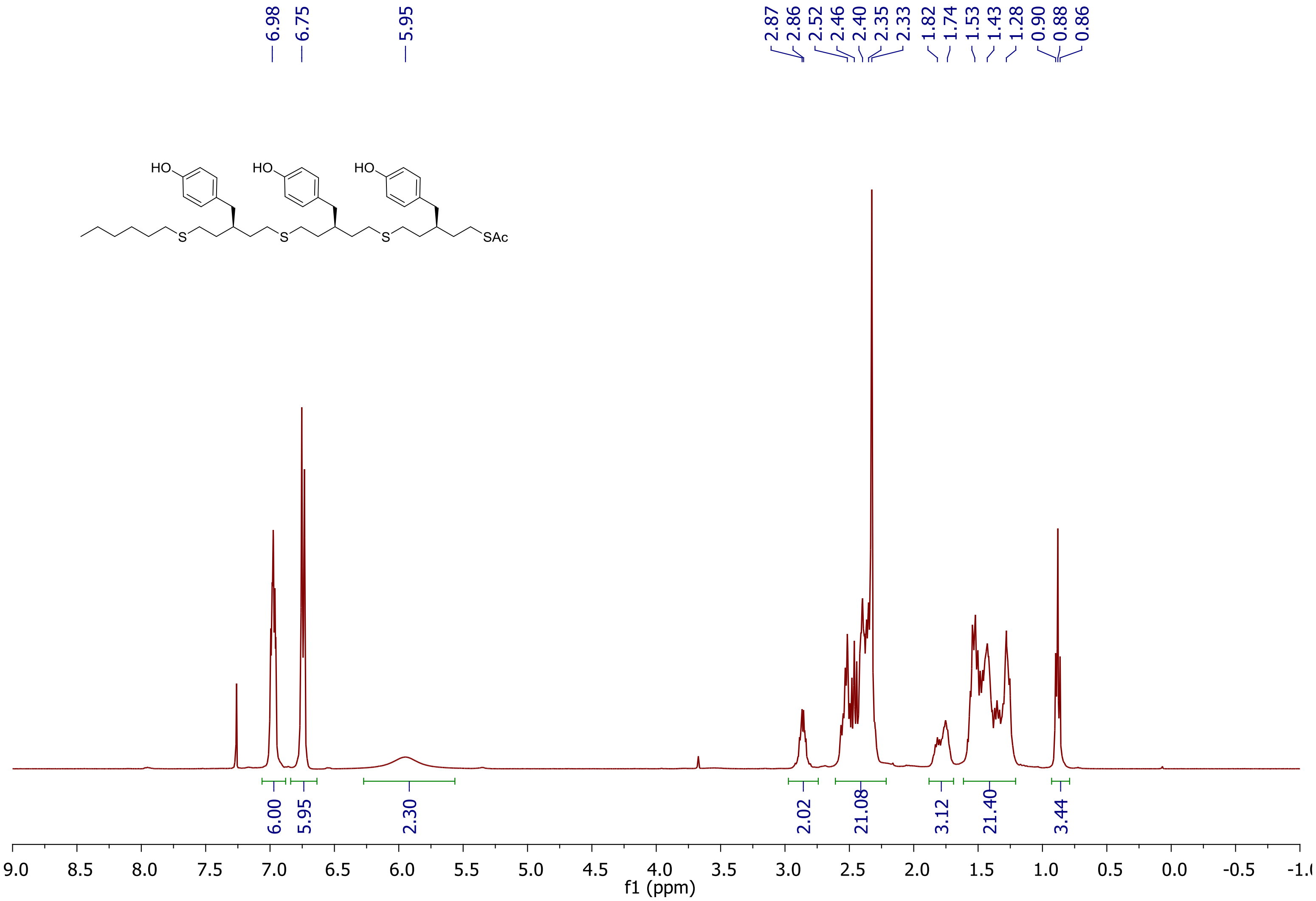

^{13}C NMR (100.6 MHz, $CDCl_3$) compound 15a.

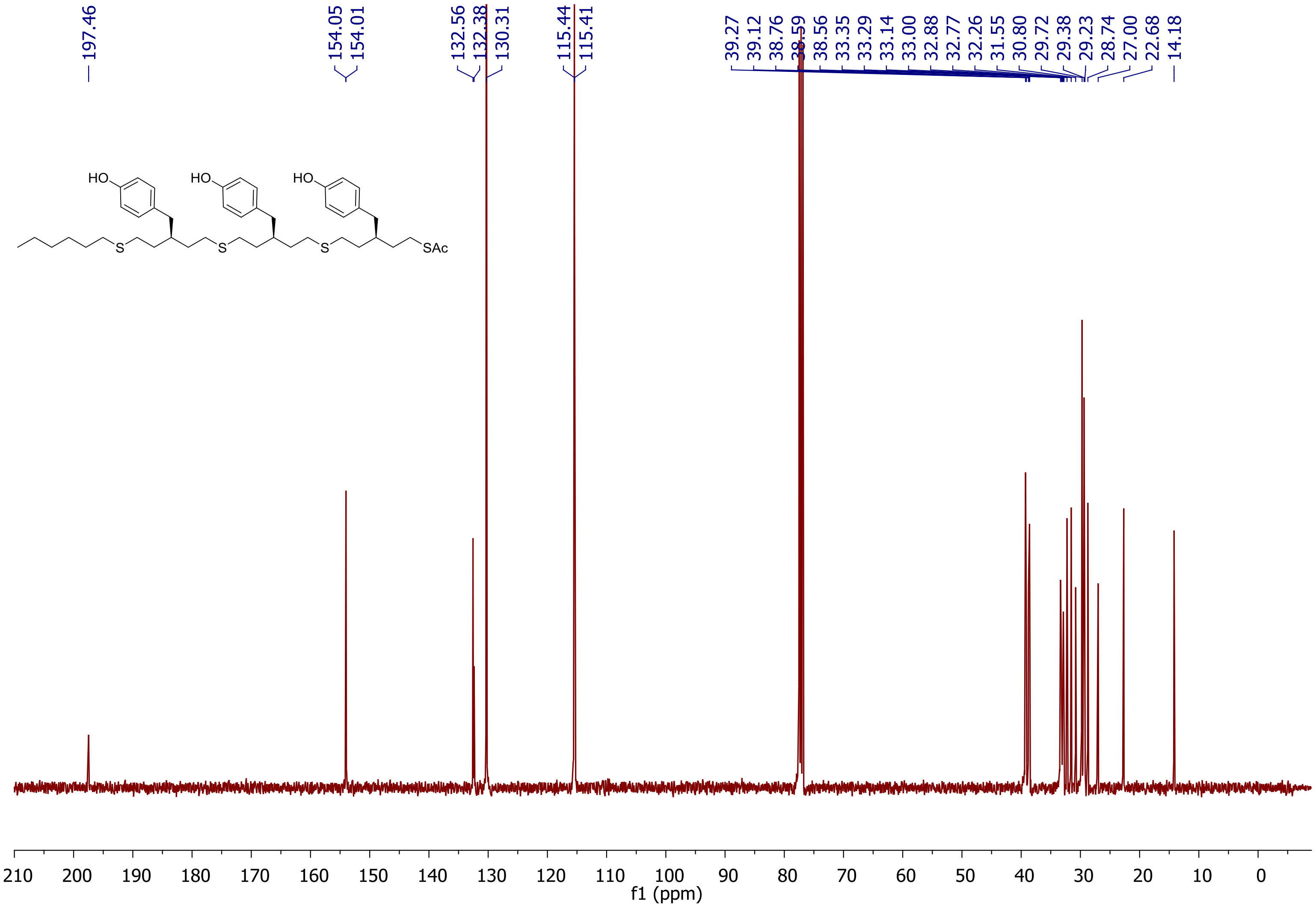

Compound 15b.

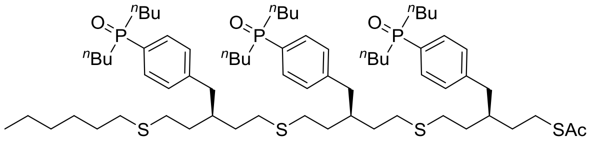

Compound **15b** was obtained from compound **14b** following the general procedure described before (Method A, UV irradiation for 2 h) as syrup (yield: 25%) using CH_2Cl_2:MeOH 20:1 as the solvent system for flash chromatography.

$[\alpha]_D^{20}$: +33.3 (*c* 0.07, $CHCl_3$).

^{1}H NMR (400 MHz, $CDCl_3$): δ_H = 7.60 (m, 6H), 7.27 (m, 6H), 2.84 (m, 2H), 2.69-2.39 (m, 18H), 2.29 (s, 3H), 1.89 (m, 15H), 1.55 (m, 20H), 1.33 (m, 20H), 0.86 (m, 21H).

^{13}C NMR (100.6 MHz, $CDCl_3$): δ_C = 195.8, 144.5, 144.4, 144.4, 144.4, 144.2, 144.2, 130.8, 130.8, 130.8, 130.7, 130.7, 130.6, 130.6, 129.9, 129.9, 129.6, 129.5, 129.5, 40.2, 40.1, 40.0, 38.4, 38.2, 33.2, 33.1, 33.1, 33.0, 32.3, 31.6, 30.8, 30.2, 29.7, 29.6, 29.6, 29.6, 29.5, 29.5, 29.5, 28.7, 26.6, 24.3, 23.7, 22.7, 14.2, 13.7.

^{31}P NMR (161.3 MHz, $CDCl_3$): δ_P = 40.7, 40.6, 40.6.

MS (ES+): m/z (%) = 1217.6 $[M+H]^+$.

HRMS (ES+): calcd for $C_{68}H_{116}O_4P_3S_4$ 1217.6969 $[M+H]^+$, found 1217.5552 $[M+H]^+$.

FT-IR (ATR): 2930, 2851, 1688, 1462, 1166, 1105, 901, 750 ν_{max}/cm^{-1}.

^{1}H NMR (400 MHz, $CDCl_3$) compound 15b.

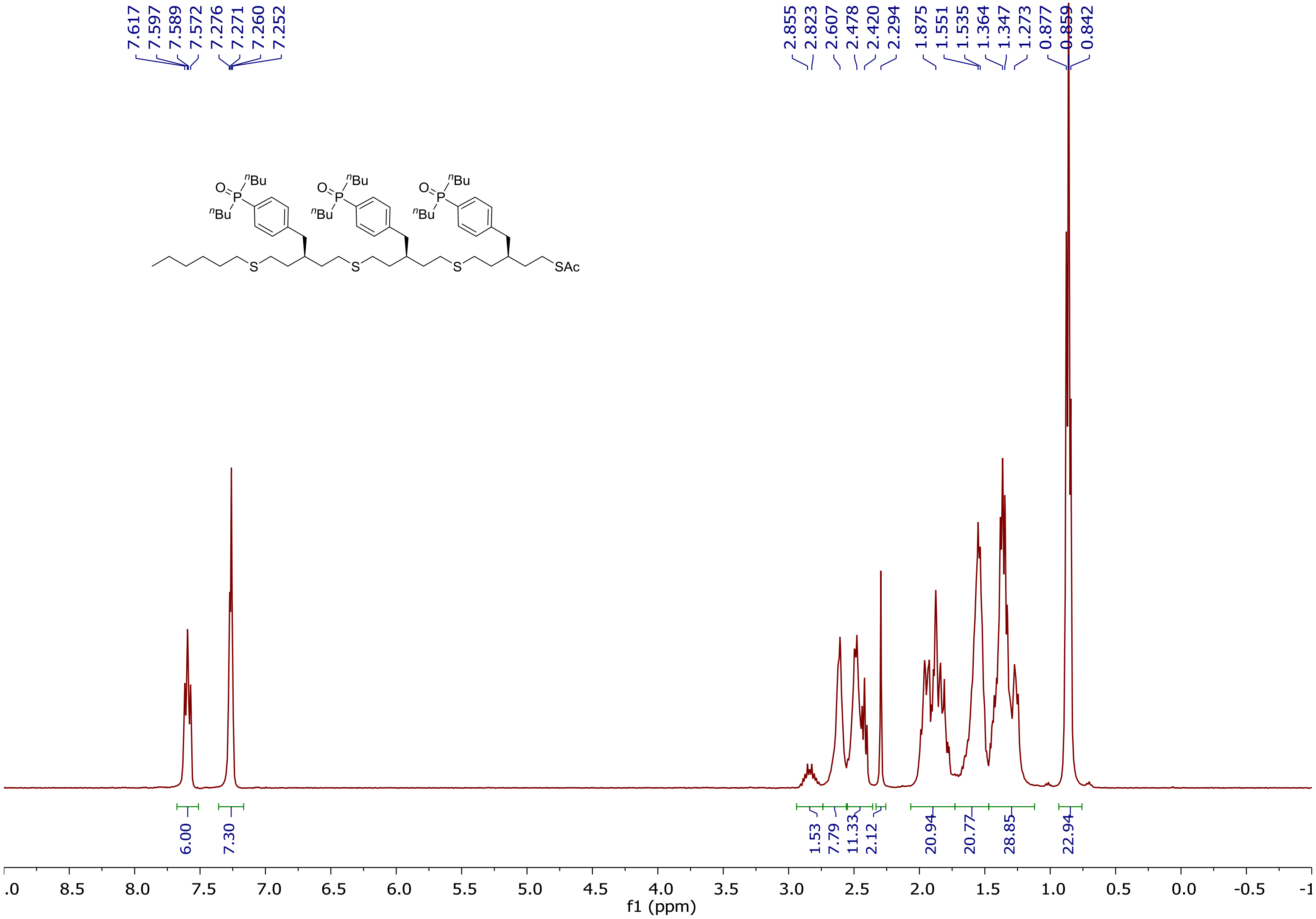

^{13}C NMR (100.6 MHz, $CDCl_3$) compound 15b.

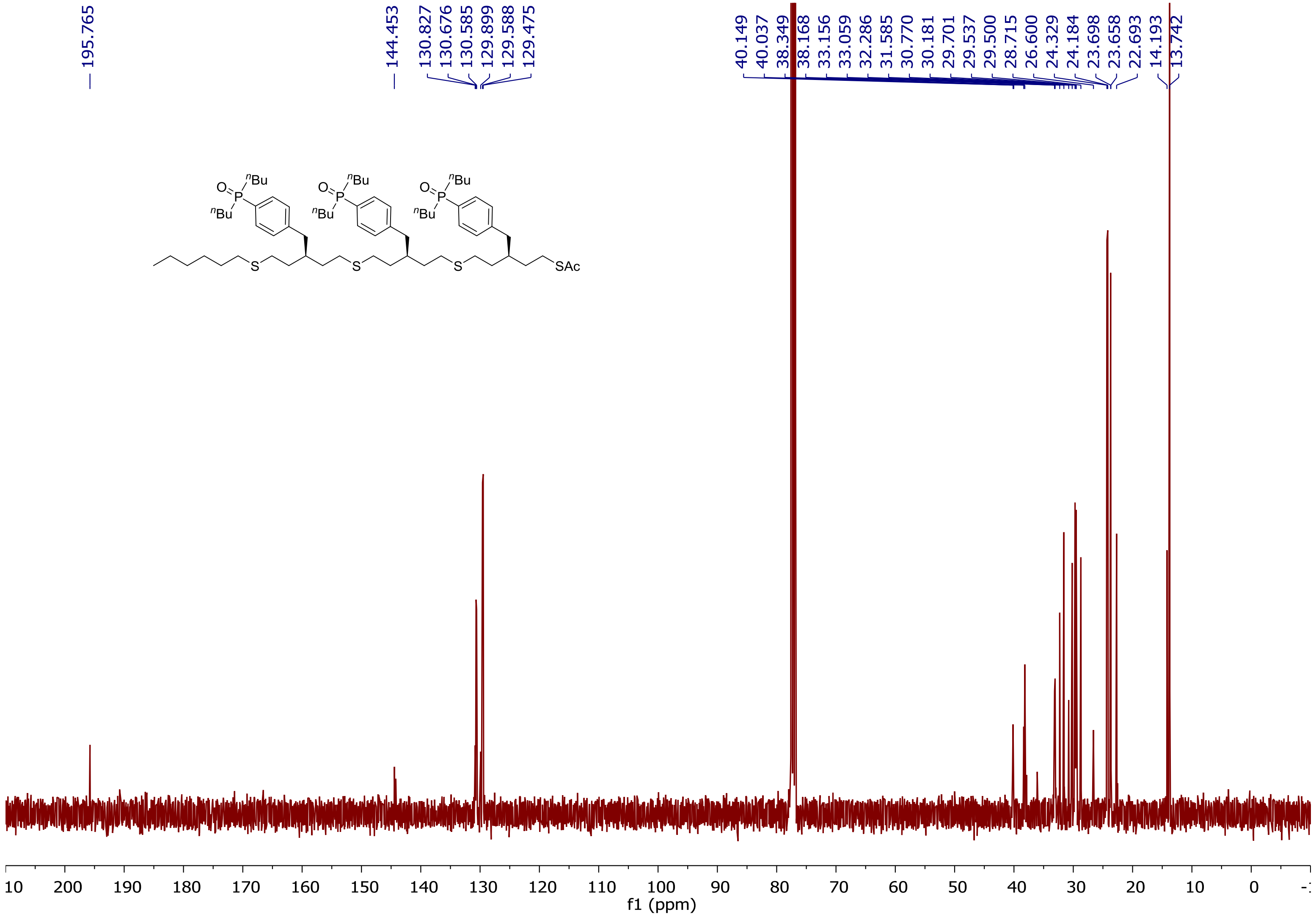

^{31}P NMR (161.3 MHz, $CDCl_3$) compound 15b.

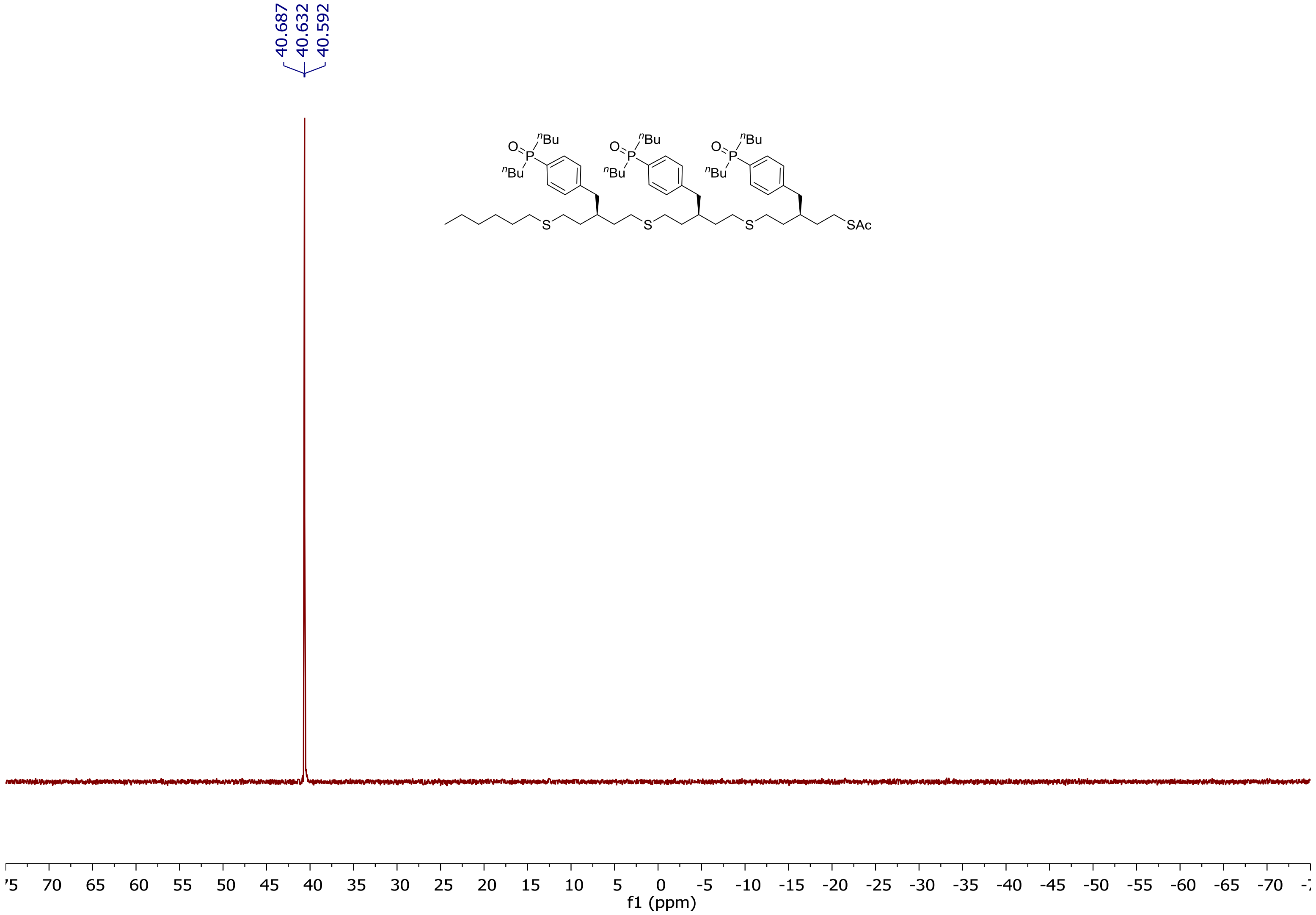

Compound 16a.

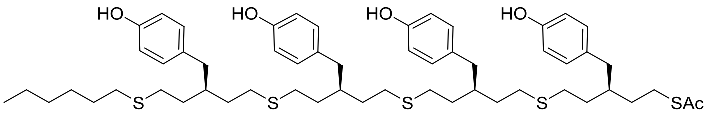

Compound **16a** was obtained from compound **15a** following the general procedure described before (Method A, UV irradiation for 2h) as a white solid (yield: 66%) using EtOAc:hexane 1:1.5 as the solvent system for flash chromatography.

Melting point: 67-69 ^{o}C.

$[\alpha]_D^{20}$: +1.7 (*c* 0.24, $CHCl_3$).

^{1}H NMR (400 MHz, $CDCl_3$): δ_H = 6.98 (m, 8H), 6.74 (m, 8H), 5.38 (bs, 4H), 2.87 (m, 2H), 2.41 (m, 24H), 2.33 (s, 3H), 1.78 (m, 4H), 1.67-1.26 (m, 22H), 0.89 (t, 3H, *J* = 7.0 Hz).

^{13}C NMR (100.6 MHz, $CDCl_3$): δ_C = 197.3, 154.1, 154.0, 154.0, 154.0, 132.7, 132.6, 132.5, 132.5, 130.3, 115.5, 115.5, 115.4, 39.3, 39.2, 38.8, 38.6, 38.6, 38.6, 33.4, 33.3, 33.1, 33.0, 32.9, 32.9, 32.3, 31.6, 30.8, 29.8, 29.8, 29.5, 29.4, 29.3, 28.8, 27.0, 22.7, 14.2.

MS (ES+): m/z (%) = 993.5 $[M+H]^+$.

HRMS (ES+): calcd for $C_{56}H_{81}O_5S_5$ 993.4688 $[M+H]^+$, found 993.4686 $[M+H]^+$.

FT-IR (ATR): 3331, 2919, 2851, 1613, 1512, 1444, 1220, 1171, 821, 736 ν_{max}/cm^{-1}.

^{1}H NMR (400 MHz, $CDCl_3$) compound 16a.

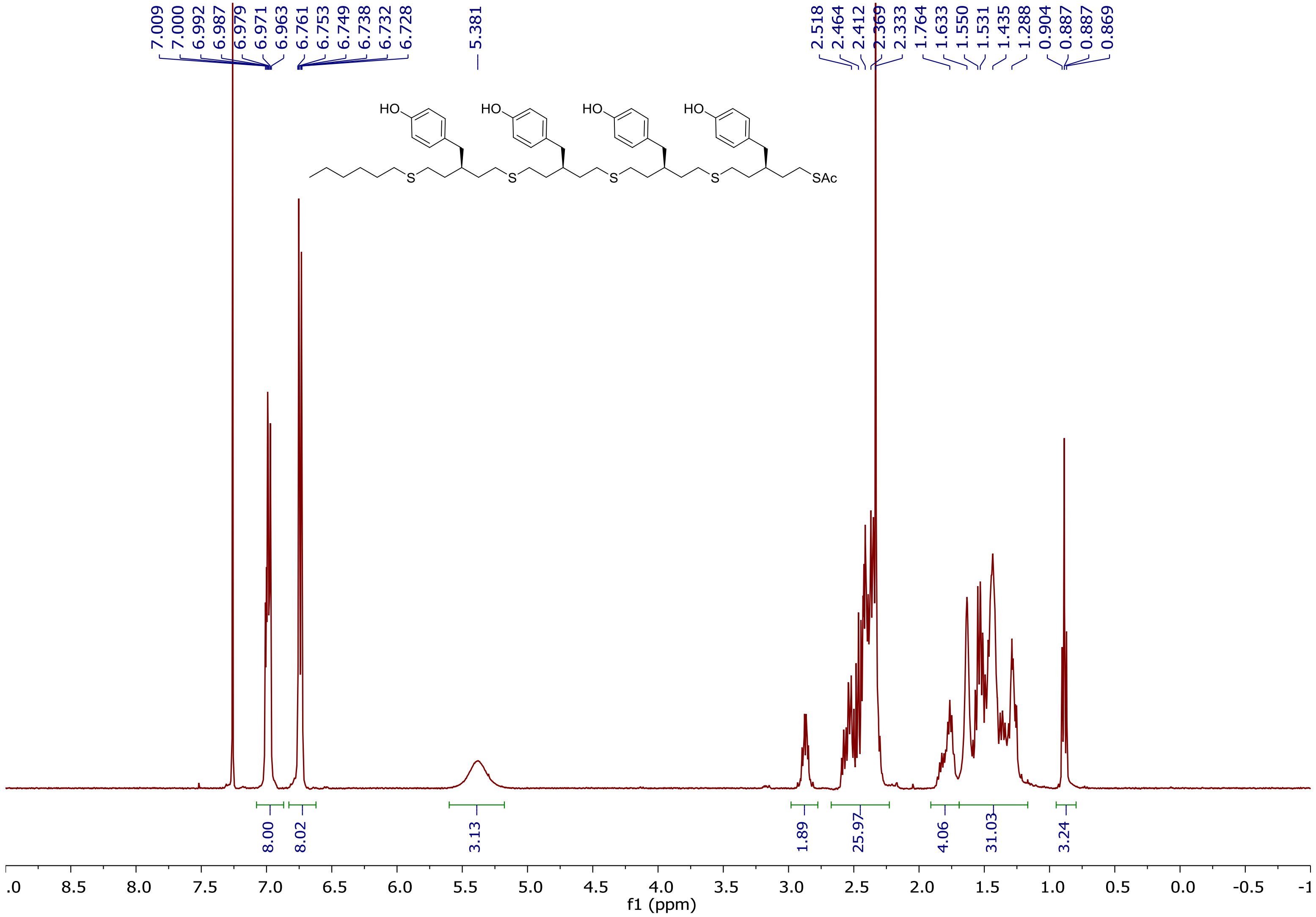

^{13}C NMR (100.6 MHz, $CDCl_3$) compound 16a.

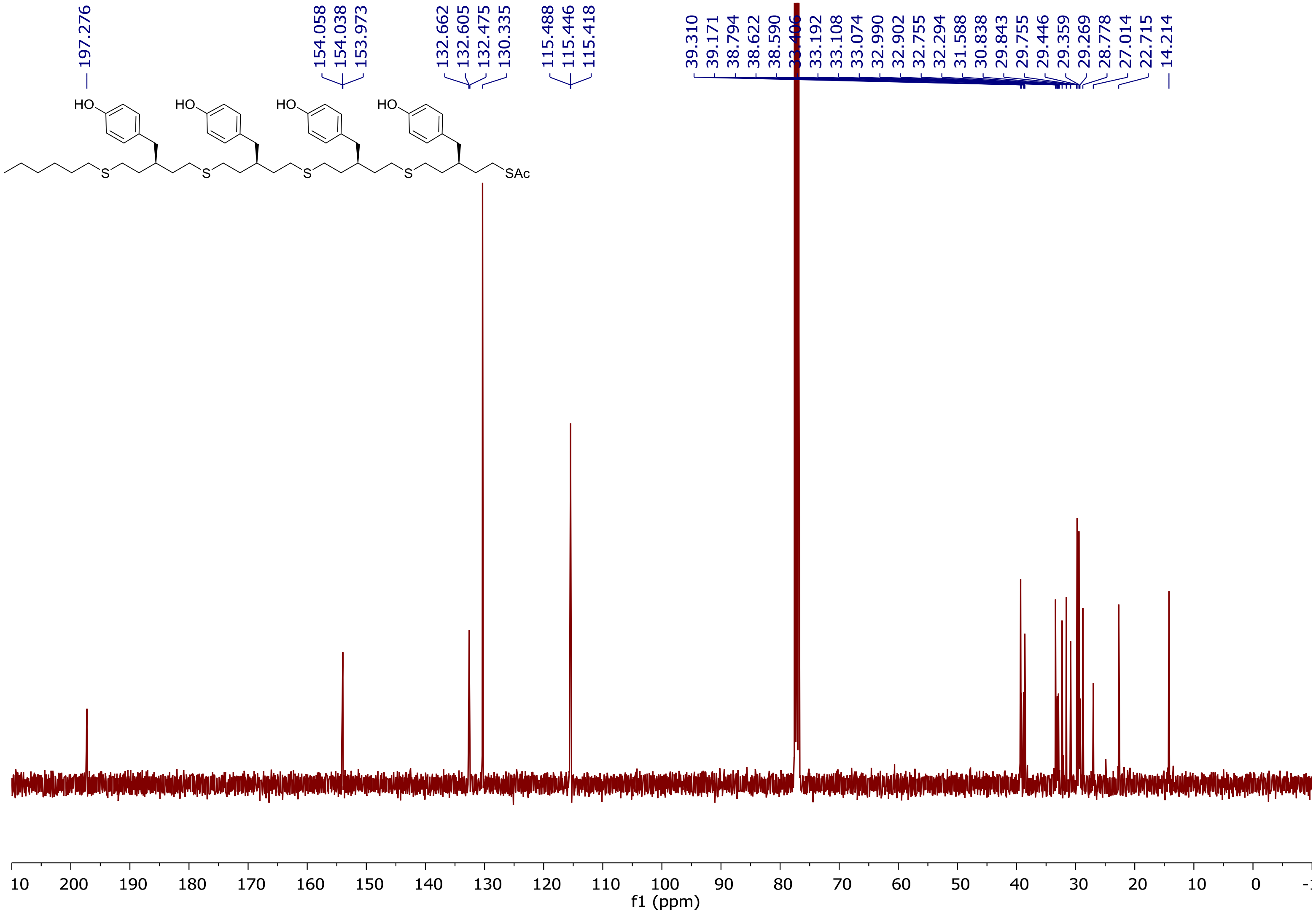

Compound 16b.

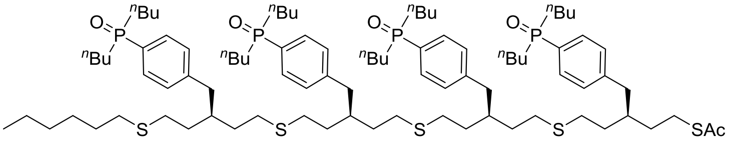

Compound **16b** was obtained from compound **15b** following the general procedure described before (Method B, UV irradiation for 2 h) as a syrup (yield: 19%) using CH_2Cl_2:MeOH 15:1 as the solvent system for flash chromatography.

$[\alpha]_D^{20}$: +2.4 (*c* 0.19, $CHCl_3$).

^{1}H NMR (400 MHz, $CDCl_3$): δ_H = 7.63 (m, 8H), 7.29 (m, 2H), 2.87 (m, 2H), 2.76-2.42 (m, 24H), 2.33 (s, 3H), 1.91 (m, 20H), 1.58 (m, 26H), 1.33 (m, 30H), 0.89 (m, 21H).

^{13}C NMR (100.6 MHz, $CDCl_3$): δ_C = 195.8, 144.5, 144.4, 144.3, 143.8, 130.8, 130.7, 130.7, 130.6, 130.6 130.1, 130.1, 130.0, 130.0, 129.6, 129.5, 40.2, 38.4, 38.2, 37.8, 37.7, 33.2, 33.1, 33.0, 32.9, 32.3, 31.6, 31.5, 30.8, 30.1, 29.9, 29.7, 29.7, 29.6, 29.5, 28.7, 24.3 (d, *J* = 14.5 Hz), 23.7 (d, *J* = 4.0 Hz), 22.7, 14.2, 13.8.

^{31}P NMR (161.3 MHz, $CDCl_3$): δ_P = 40.6, 40.6, 40.6, 40.5.

MS (ES+): m/z (%) = 1569.9 $[M+H]^+$.

HRMS (ES+): calcd for $C_{88}H_{149}O_5P_4S_5$ 1569.8959 $[M+H]^+$, found 1569.9033 $[M+H]^+$.

FT-IR (ATR): 2955, 2926, 2860, 1456, 1405, 1166, 1112, 902, 797 ν_{max}/cm^{-1}.

^{1}H NMR (400 MHz, $CDCl_3$) compound 16b.

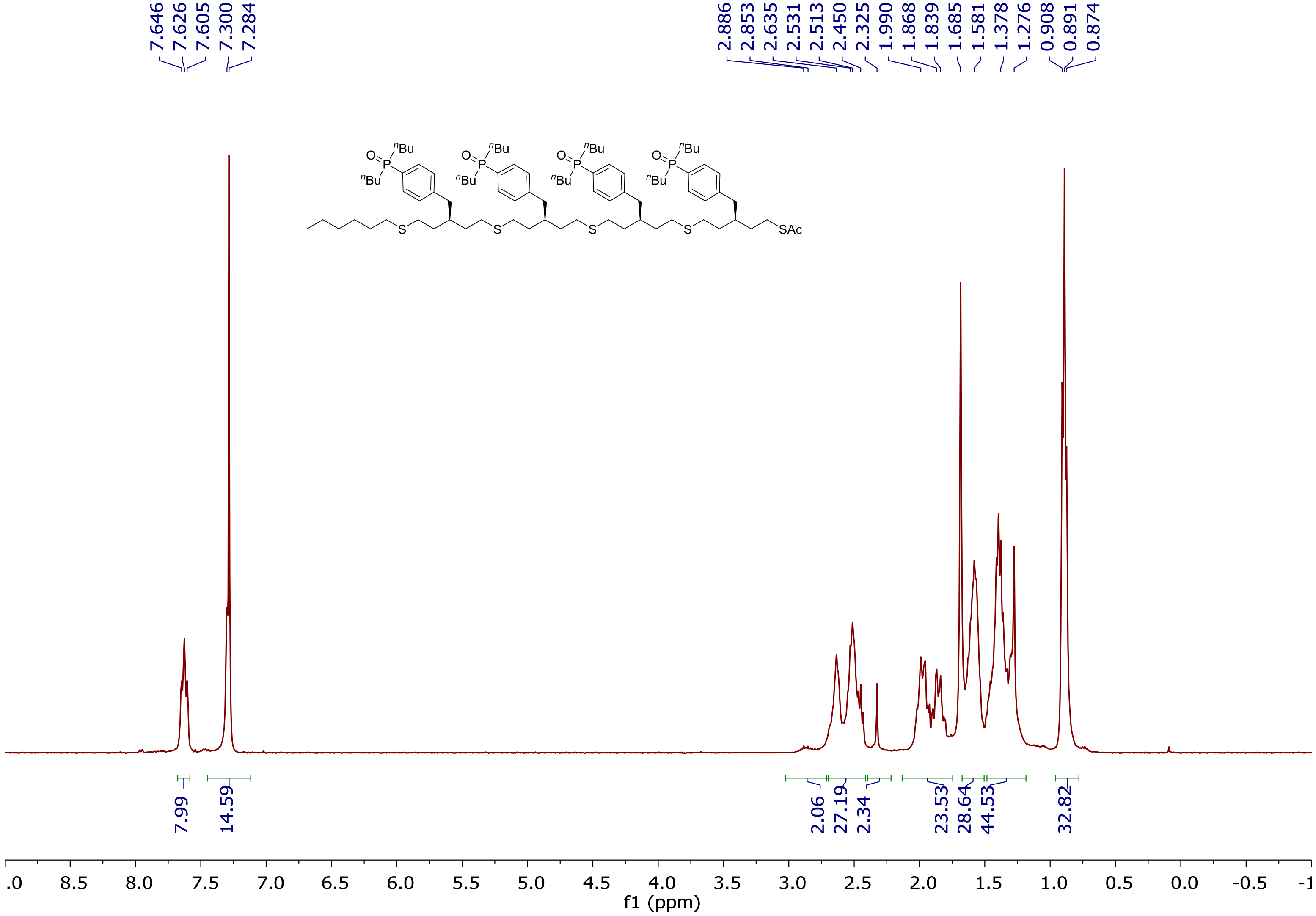

^{13}C NMR (100.6 MHz, $CDCl_3$) compound 16b.

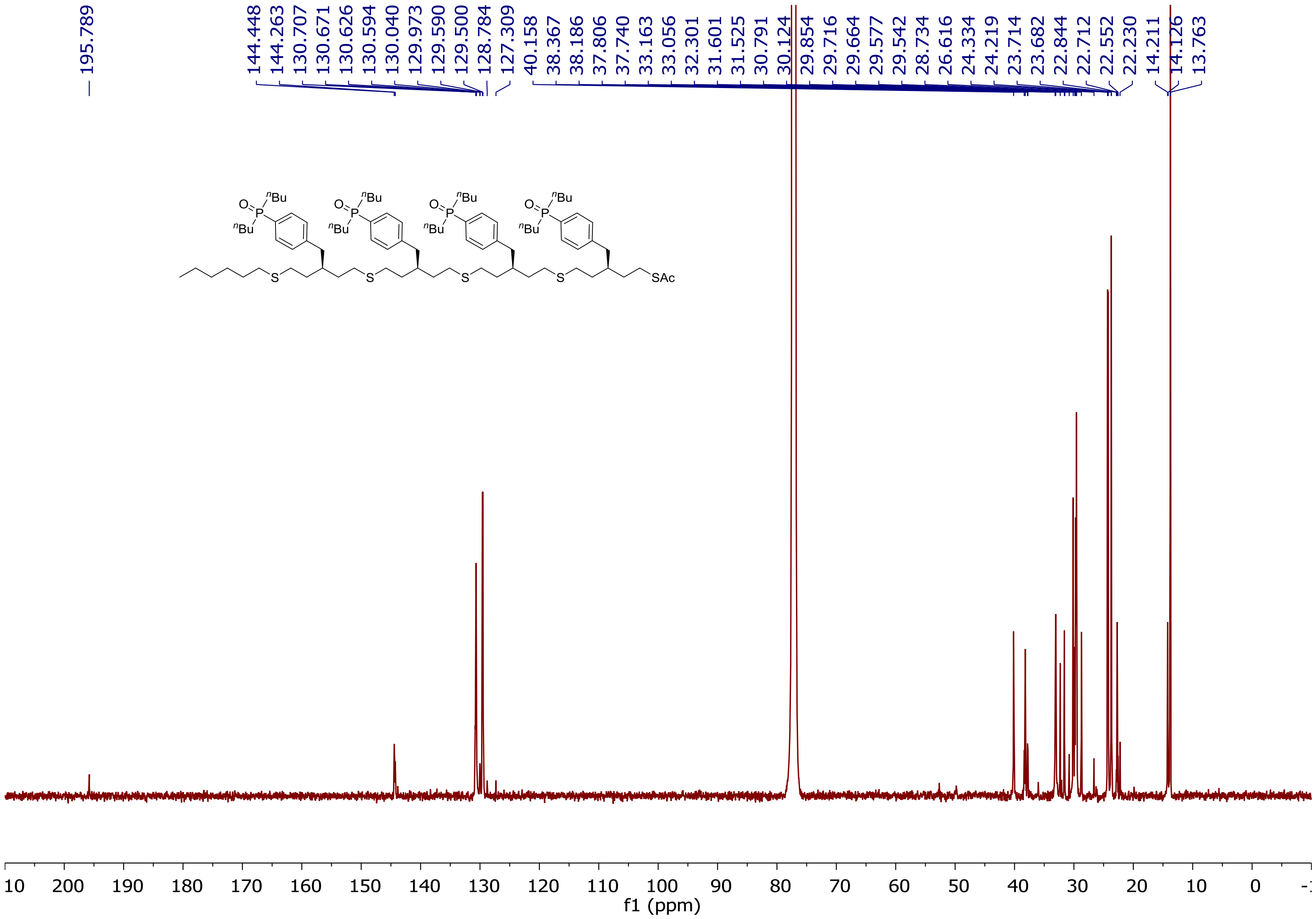

^{31}P NMR (161.3 MHz, $CDCl_3$) compound 16b.

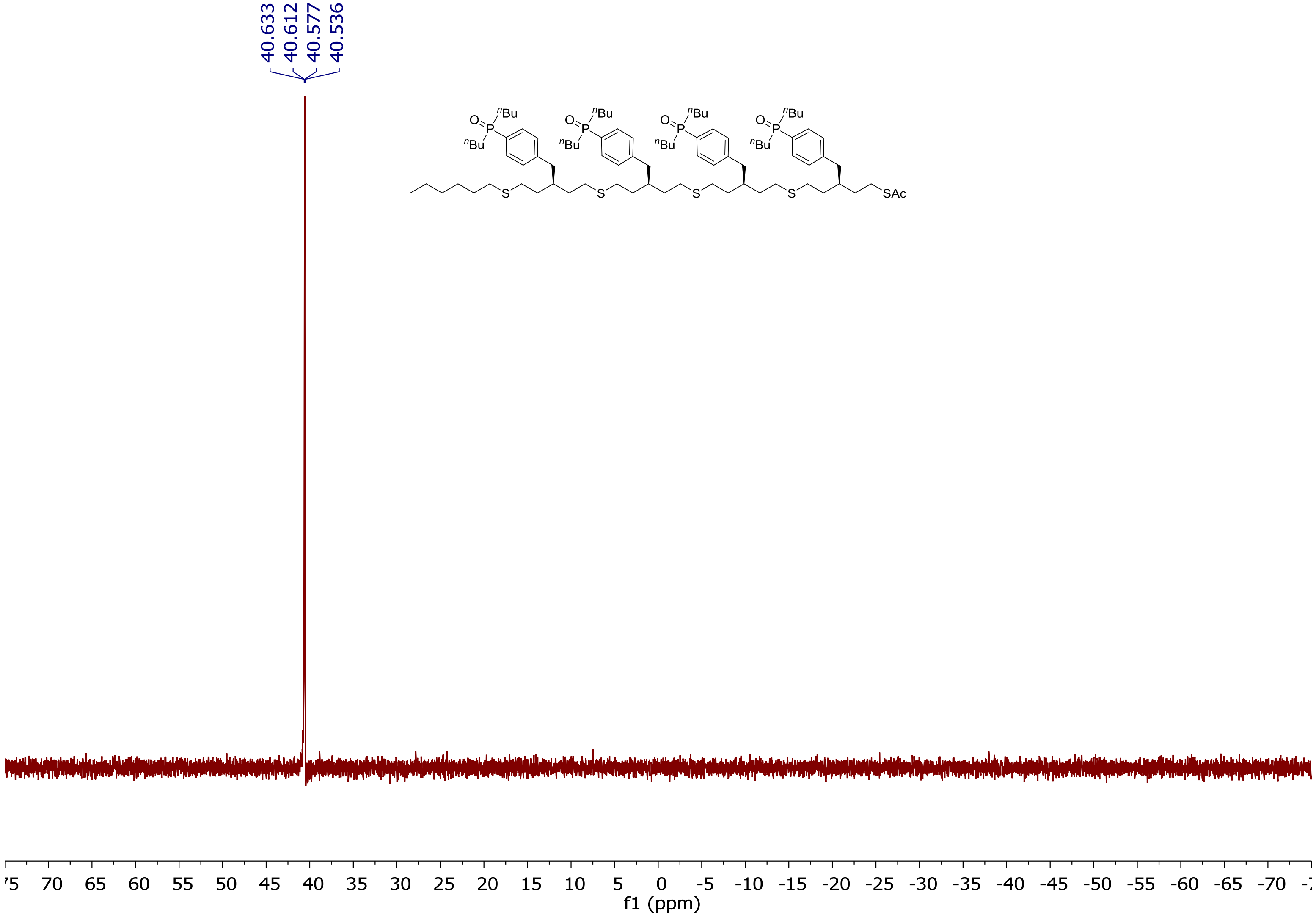

Supplement: Supplementary file 1 [file SC-008-C6SC02995G-s001.pdf]
